# Identification of Novel Molecular Panel as Potential Biomarkers of PAN-Gastrointestinal Cancer Screening: Bioinformatics and Experimental Analysis

**DOI:** 10.3390/biology14070803

**Published:** 2025-07-02

**Authors:** Fatemeh Hajibabaie, Parisa Mohamadynejad, Laleh Shariati, Kamran Safavi, Navid Abedpoor

**Affiliations:** 1 Department of Biology, ShK.C., Islamic Azad University, ShahreKord 88137-33395, Iran; fatemeh.hajibabaie@iau.ir; 2 Department of Biomaterials, Nanotechnology and Tissue Engineering, School of Advanced Technologies in Medicine, Isfahan University of Medical Sciences, Isfahan 81746-73461, Iran; shariati_l59@yahoo.com; 3 Biosensor Research Center, Isfahan University of Medical Sciences, Isfahan 81746-73461, Iran; 4 Department of Agricultural Biotechnology and Engineering, Institute of Agriculture, Water, Food and Nutraceutical, Isf.C., Islamic Azad University, Isfahan 81551-39998, Iran; kamran.safavi@iau.ac.ir; 5 Research Center of Medicinal Plants, Isf.C., Islamic Azad University, Isfahan 81551-39998, Iran; navid.abedpoor@iau.ac.ir; 6 Department of Sports Physiology, Isf.C., Islamic Azad University, Isfahan 81551-39998, Iran

**Keywords:** PAN-gastrointestinal cancers, bioinformatics, *AURKA*, *CEP55*, *DTL*, *TTK*

## Abstract

PAN-gastrointestinal cancers (PAN-GI cancers), including the oral, esophageal, gastric, hepatocellular, pancreatic=, and colorectal cancers, are the leading cause of cancer-related mortality. Despite recent advances in identifying the molecular mechanisms driving these malignancies, the high incidence and recurrence of the PAN-gastrointestinal cancers and the low survival rates of patients indicate the need to introduce biomarkers for early diagnosis to improve diagnostic and therapeutic approaches. In the present study, using integrated transcriptomics, RNA-Seq and microarray data, from the TCGA and GEO databases, respectively, were combined to discover and validate a global biomarker panel for PAN-gastrointestinal cancers. In order to validate the bioinformatics data, the expression levels of genes in the molecular panel were evaluated using real-time quantitative polymerase chain reaction (qPCR) in tumor tissues of 21 patients with early diagnosis of gastric cancer and colorectal cancer (Stage I and II). By examining the transcriptomic profiles of six types of PAN-gastrointestinal cancers, a network of closely related hub genes (*n* = 167) with biomarker potential (*p* value < 0.05) was identified. Also, using ROC curve analysis and the Youden index, a molecular panel consisting of *AURKA*, *CEP55*, *DTL*, and *TTK* was presented (95% confidence interval and *p* value < 0.05), which showed exceptional sensitivity and specificity in differentiating malignant tissue from normal tissue (AUC > 80%). The diagnostic efficacy of these markers was confirmed by further validation using qPCR in colorectal and gastric tumor samples (*p* value < 0.05). In conclusion, a novel molecular signature panel including the *AURKA*, *CEP55*, *DTL*, and *TTK* genes could improve early cancer detection and diagnostic accuracy, and it may contribute to the treatment outcomes of PAN-gastrointestinal cancer patients.

## Simple Summary

Gastrointestinal cancers, including malignancies of the oral cavity, esophagus, stomach, pancreas, liver, and colon, remain significant contributors to cancer-related mortality due to late detection and limited therapy options. This research employed a novel, integrated transcriptomics methodology by analyzing data from public repositories (TCGA and GEO) to uncover the common molecular characteristics of these tumors. The researchers first identified frequently differentially expressed genes and constructed protein–protein interaction networks, pinpointing 167 overlapping “hub” genes. Subsequent enrichment analysis refined this list to 16 promising candidate biomarkers. Four genes—*AURKA*, *CEP55*, *DTL*, and *TTK*—demonstrated significant diagnostic potential. Receiver operating characteristic (ROC) curve studies indicated that these four genes, used either individually or in combination, could effectively differentiate malignant from normal tissues, achieving area under the curve (AUC) values exceeding 0.80. Ultimately, experimental validation through quantitative PCR in gastric and colorectal cancer tissue specimens confirmed that the transcript levels of *AURKA*, *CEP55*, *DTL*, and *TTK* are markedly elevated in tumor tissues compared to normal tissues. This work highlights the effectiveness of utilizing an integrated bioinformatics and laboratory validation approach to develop universal genetic biomarkers for the early diagnosis of PAN-gastrointestinal malignancies. These biomarkers may enhance diagnostic accuracy, enable timely interventions, and ultimately improve patient outcomes.

## 1. Introduction

The gastrointestinal (GI) system consists of the gastrointestinal tract (GIT), including the esophagus, stomach, and intestines, and auxiliary organs such as the liver, gallbladder, and pancreas [1]. The incidence and mortality of malignancies in these areas are anticipated to escalate markedly, with forecasts indicating a 58% increase in cases and a 73% increase in deaths by 2040, amounting to 7.5 million and 5.6 million, respectively. The prevalence of gastrointestinal carcinomas and associated mortality, which account for approximately half of all cancer-related deaths, has highlighted the need for improved diagnostic and therapeutic strategies [2,3]. GI cancer encompasses malignancies originating in the oral cavity, esophagus, stomach, liver and bile ducts, gallbladder, pancreas, small intestine, colon, and rectum. Each of these malignancies exhibits specific biological changes and clinical features that may be due to distinct cellular and tissue origins [4,5]. Although each gastrointestinal malignancy has distinctive biological traits stemming from different cellular and tissue origins, growing evidence suggests shared molecular features among these tumors. Notwithstanding progress in traditional therapies, including surgery, chemotherapy, and radiation, the prognosis for several gastrointestinal malignancies continues to be unfavorable, highlighting the urgent need for enhanced diagnostic and therapeutic approaches.

PAN-gastrointestinal cancers, including malignancies of the esophagus, stomach, pancreas, liver, and colon, represent a significant global health burden due to their high incidence and mortality rates. Although advances have been made in gold-standard treatments such as surgery, chemotherapy, and radiotherapy, the poor prognosis of many PAN-gastrointestinal cancers hampers treatment, suggesting an urgent need for innovative approaches to understand the molecular mechanisms better, improve diagnostic accuracy, and identify practical therapeutic targets. However, it is undeniable that there are significant similarities among gastrointestinal malignancies, and it is crucial to comprehend these illnesses as a unified entity.

A comprehensive analysis of gastrointestinal malignancies reveals a convergent molecular and clinical landscape, underscoring the need for a unified approach to comprehend the underlying biology of these cancers. This analysis has resulted in the analysis of PAN-GI cancers, which aims to discern shared molecular markers and pathways underpinning tumorigenesis [6]. In recent decades, significant advancements have been made in understanding the molecular processes of PAN-gastrointestinal cancers’ formation. As genomic analysis in PAN-gastrointestinal cancers has advanced, advances in genomic analysis have enabled transcriptome-based subtyping of PAN-gastrointestinal cancers [7,8]. The process of categorizing PAN-GI cancers based on transcriptome analysis identifies the genetic characteristics linked to tumor growth and incorporates several elements that lead to tumor heterogeneity [3,9].

The rising incidence of GI cancers, along with increasing recurrence and metastasis rates, has led to poor survival outcomes. A deeper understanding of PAN-gastrointestinal cancers’ molecular mechanisms is crucial for identifying etiological factors, improving diagnostics, and developing innovative treatment strategies [10,11,12,13]. In this regard, integrating omics data is vital for revealing concealed information about cancer and identifying the master biomarkers with high sensitivity and specificity in primary cancer occurrences in pan-cancer investigations.

This study introduces an integrative transcriptomic analysis approach to identify novel diagnostic biomarkers for PAN-gastrointestinal cancers. By applying an innovative ROC curve and Youden index-based scoring method, we sought to identify a biomarker panel with high sensitivity and specificity for the diagnosis of PAN-gastrointestinal cancers. Significant transcriptome irregularities identified through bioinformatics analysis were confirmed using real-time PCR in the most prevalent gastrointestinal tumors, specifically gastric and colorectal cancers. This research provides new insights into gastrointestinal cancer biology and promotes improved diagnostic approaches.

## 2. Materials and Methods

### 2.1. Study Design

Our study’s subject matter was limited to six gastrointestinal tumor types from TCGA data and microarray datasets: head and neck, esophageal, stomach, pancreatic, liver, and colorectal malignancies. The number of raw RNA-Seq and microarray data from GEO and TCGA, comprising tumor and normal samples, for the 6 GI malignancies are shown in Table 1.

In the process of selecting samples for our study on head and neck regions, we strategically focused on key anatomical areas within the oral cavity and tongue, guided by the available clinical data. The distribution of samples revealed a predominance in the tongue, NOS category, with 140 cases, underscoring its significance in our analysis. This was followed by overlapping lesions of the lips, oral cavity, and pharynx, which accounted for 83 cases; and the mouth, with 23 cases. Additionally, we included samples from the floor of the mouth (57 cases), ventral surface of the tongue (1 case), base of the tongue (26 cases), and border of the tongue (1 case). These selections were made carefully, balancing both the anatomical relevance of each region and the frequency of data available, to ensure a comprehensive and representative dataset for our study.

A novel computational technique was developed using pooled data, gene expression data, and PPI networks to determine which genes interact the most. To further understand PAN-gastrointestinal cancers, we conducted several studies that included analyzing differences in gene expression between GI malignancy samples and non-GI samples at the in silico and in vitro wet laboratory levels.

### 2.2. Ethical Code

The criteria for this study were in accordance with the Declaration of Helsinki and were approved by the research project’s Ethics Committees of the Islamic Azad University—Shahrekord Branch (IR.IAU.SHK.REC.1402.145).

### 2.3. Integrative Analysis of Microarray and RNA-Seq Data: A Pathway to Biomarker Discovery in Gastrointestinal Malignancies

#### 2.3.1. Microarray

The PAN-gastrointestinal cancers microarray dataset was obtained from the GEO database, which can be accessed at https://www.ncbi.nlm.nih.gov/geo (accessed on 27 June 2025), using the keyword phrases “GSE138206-Oral Squamous Cell Carcinoma”, “GSE75241-Esophageal Cancer”, “GSE54129-Gastric Cancer”, “GSE132956-Pancreatic Ductal Adenocarcinoma”, “GSE164760- Hepatocellular Carcinoma”, and “GSE110224-Colorectal Adenocarcinoma”. Microarray sample datasets were evaluated using R programming language packages (version 4.4.3) and the Bioconductor tool (version 3.21) to discover transcripts exhibiting differential expression (DEGs). MAS5/RMA methods were used in data standardization. The datasets were analyzed using a *t*-test, and genes with significant differential expression at a *p* value < 0.05 significance level were identified. A heatmap graphic was created using the ggplot program (Version: 3.5.2) for genes with a *p* value < 0.05.

Moreover, to identify the key regulatory genes in the pathogenesis of PAN-gastrointestinal cancers, we used a logarithm-transformed logFC ± 1 threshold to group over- or underexpressed genes. Next, the STRING 11.5 database was used to construct a network of protein–protein interactions (PPIs), with hub nodes from each study dataset. As specified, we emphasized medium confidence 0.4 and identified hub genes using CytoScape 3.6.0 to display network metrics (degree, betweenness centrality, and closeness centrality). CytoScape is an open-source computational biology platform designed to visualize biological interaction networks based on various network parameters. Also, this approach facilitates the integration of gene expression profiles and other statistical datasets, with advanced functionalities available through a wide range of plugins [14].

The genetic network of hub nodes was designed using network diameters, eigenvector centrality, and modularity class to optimize the visualization of large graphs. PPIs were determined through the Gephi 9.2.0 platform, a sophisticated software platform designed to visualize and analyze complex networks [15]. The Enrich-KG database was used to perform gene set enrichment analysis to discover the crucial molecular signaling pathways and gene ontology processes associated with hub genes with substantial differential expression in colorectal cancer [16]. Then, the genetic network, which includes these central genes, was created using Gephi software 9.2.0.

#### 2.3.2. RNA-Seq

Transcriptome RNA-sequencing datasets were downloaded from “https://portal.gdc.cancer.gov/ (accessed on 27 June 2025)” in FPKM units using the R programming language [17,18]. Transcriptome (RNA-Seq) data for each cancer were downloaded in raw format using the TCGAbiolinks package [19,20]. Also, clinical data were downloaded from the TCGA database, previously published at http://cancergenome.nih.gov/ (accessed on 27 June 2025). Then, genes with relatively minor or negligible expression were removed using CPM (count per million) metrics, supported by the edgeR package [21]. Furthermore, data normalization was performed using the Limma and TMM approach [22,23]. Expression data for each gene and sample were logarithmically transformed using the Limma package, based on a factor of 2 (Voom method), and were archived as a normalized expression matrix. Based on clinical information, samples from each cancer in the PAN-gastrointestinal tract cancers were classified into normal and cancerous groups. The expression difference between cancer and normal samples was assessed with a linear model approach for all transcripts. Finally, the criteria |logFC| > 1 and *p* value < 0.05 were applied to select genes that had expression changes between tumor and normal samples. Venn diagrams were used to identify commonality genes in the list of malignancy genes [24]. The analysis focused on the cellular, molecular, and signaling pathways associated with these hub genes, employing data enrichment algorithms on the Enrich-R server. The genetic interaction network was constructed using the String 11.5 server and evaluated using the CytoScape program. The scoring process employed network metrics such as degree, betweenness centrality, and closeness centrality [25]. The genetic interaction network was completed using the Gephi software to identify potential markers for molecular tests and recommend therapeutic targets for drug discovery.

In all six gastrointestinal cancer subtypes, AUC cut-off values greater than 0.8 were considered to distinguish tumor tissue from normal tissue. Finally, the expression of the selected potential genes was also investigated by real-time PCR at the experimental level.

### 2.4. Participating in the Experimental Wet Lab

To confirm the results of the bioinformatics analysis, tissue samples were obtained from 21 patients with early diagnosis of gastric cancer or colorectal cancer, through endoscopy and colonoscopy, respectively. The sample size was estimated with an effect size of 0.8, an α error probability of 0.05, and a power of 0.8, using G*Power 3.1 [26]. The clinical information of the patients is presented in Table 2.

### 2.5. RNA Isolation, cDNA Synthesis, and Optimizing qPCR Performance

To extract total RNA from the tissue biopsies, tissues were washed three times with PBS to remove blood, contamination, and necrotic cells. Subsequently, total RNA was extracted using TRIzol^®^ reagent (Sigma-Aldrich, St. Louis, MO, USA), following the manufacturer’s instructions. To remove any potential DNA contamination during RNA extraction, DNase I treatment (Sinnaclon, Mashhad, Iran) was performed. Then, cDNA was synthesized for all samples using a kit (BioFact™, Daejeon, Republic of Korea), according to the original method. Hub gene-specific primers were designed using BIACON designer software 7 and the Oligo7 program 7.60 and confirmed by the NCBI Primer-BLAST tool (https://www.ncbi.nlm.nih.gov/tools/primer-blast (accessed on 27 June 2025)) (Table 3). To evaluate the relative expression of genes, RT-qPCR was performed using SYBR Green PCR Master Mix (ADDBIO Inc., Seoul, Republic of Korea), 10 pmol/µL of each primer, and 50 ng of cDNA in a final volume of 20 µL for each reaction. The *B2M* gene was used as a housekeeping gene to normalize the expression levels. Each reaction was repeated three times to ensure accuracy, and the results were evaluated using the ΔCt method.

### 2.6. Statistical Analysis

The normality of the data was assessed using the Shapiro–Wilk test. Data analysis was conducted using *t*-tests, employing GraphPad Prism statistical software (version 10.3, GraphPad, San Diego, CA, USA). Statistical significance was considered at a *p* value < 0.05, with the mean ± standard deviation (SD). In addition, the present study utilized receiver operating characteristic (ROC) curve analysis to evaluate PAN-gastrointestinal cancer-related critical diagnostic indicators.

## 3. Results

### 3.1. Integrated Biomarker Discovery in PAN-Gastrointestinal Cancers

#### 3.1.1. Pinpointing Key Drivers of Gastrointestinal Malignancy Through Integrated Microarray Data Analysis

**Figure 1:**
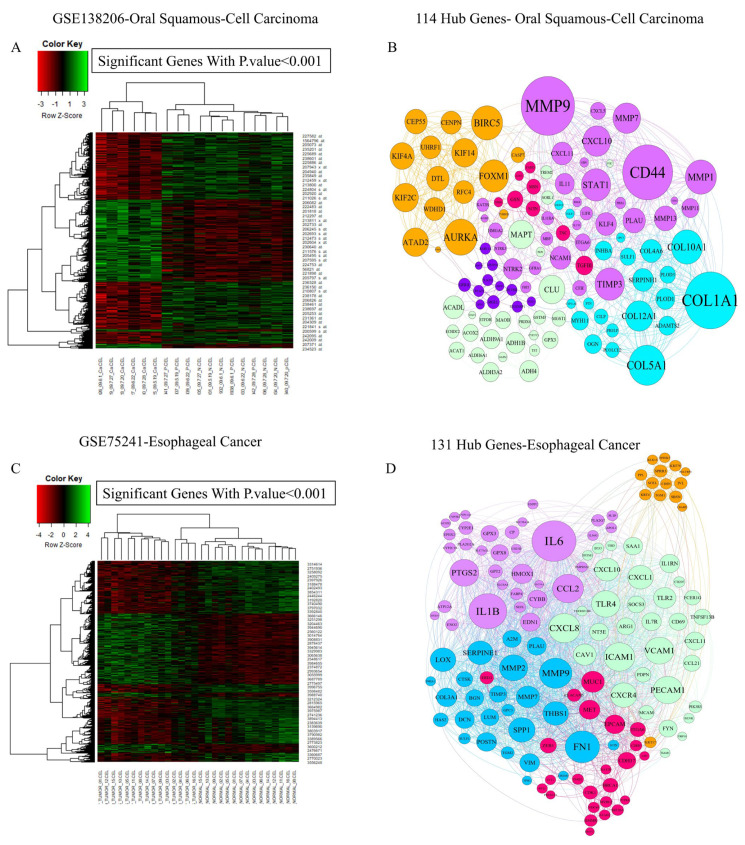

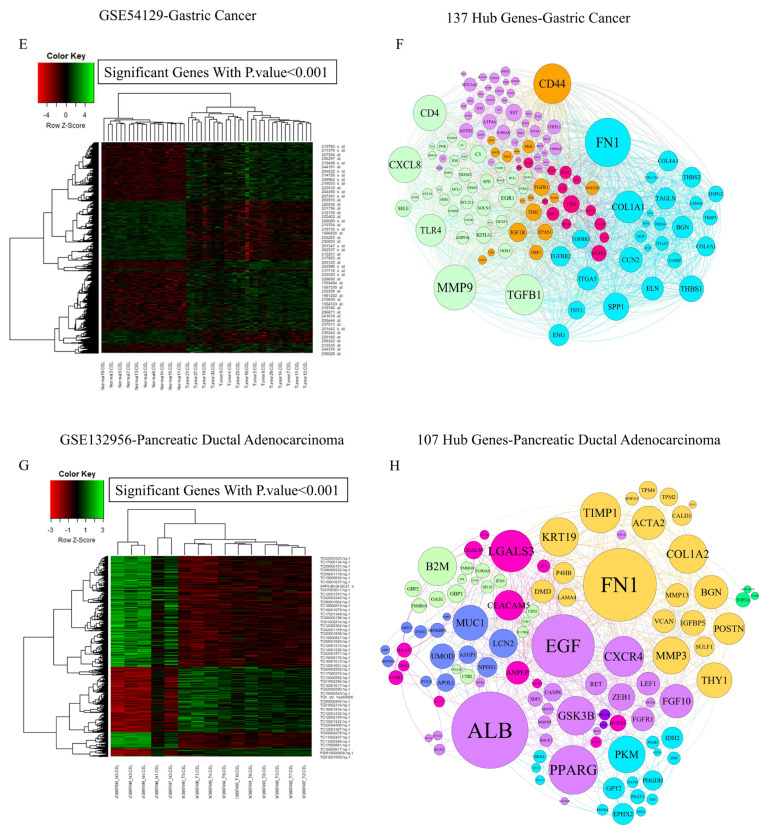

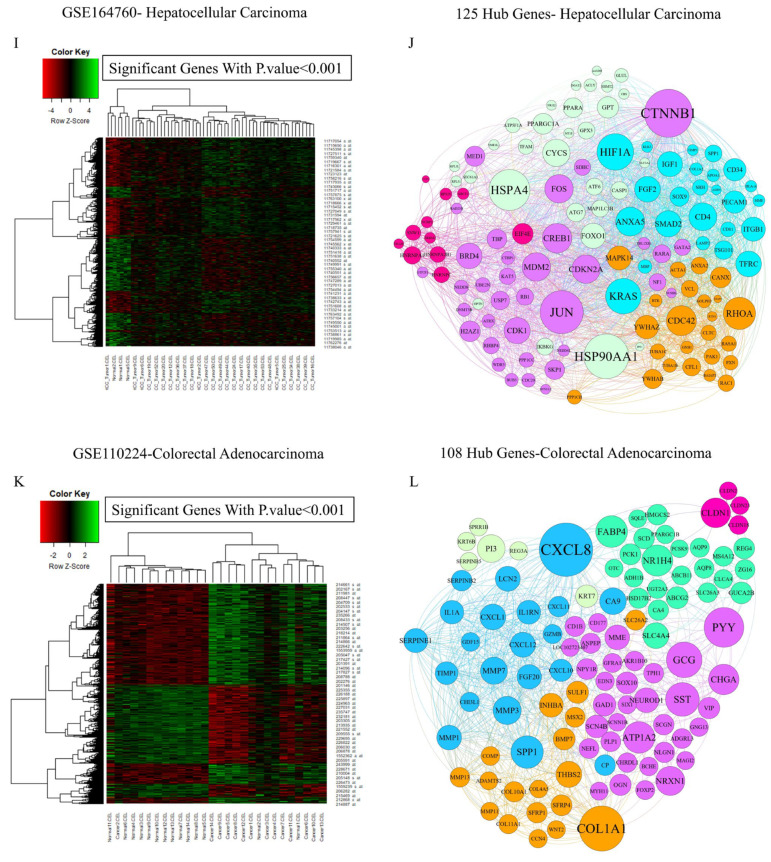

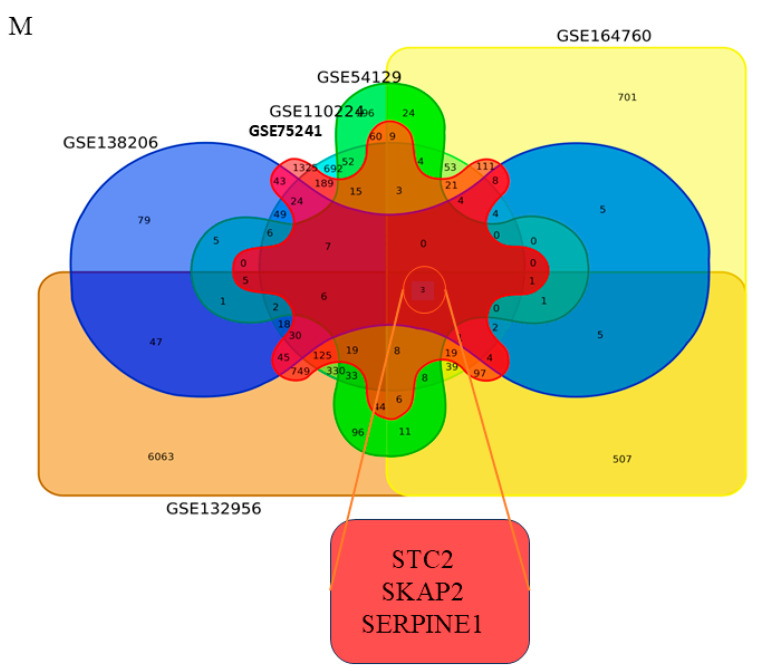
**Microarray platform identification of hub genes:** (**A**–**L**) The heatmap diagrams illustrate the depiction of significant differentially expressed genes (DEGs) in PAN-gastrointestinal cancers using the microarray dataset. The filtering of DEGs was based on a *p* value threshold of less than 0.05. Networks of protein–protein interactions (PPIs) were constructed and discovered in the contribution of hub genes in forming and advancing PAN-gastrointestinal cancers. The networks were analyzed based on network diameters. (**M**) The Venn diagram bioinformatics tool was employed to identify shared genes that were present in the microarray datasets. This analysis revealed a total of 3 genes that were common among the important markers, as determined by adj. *p* values.

Microarray datasets of oral squamous-cell carcinoma (GSE138206) (Figure 1A,B), esophageal cancer (GSE75241) (Figure 1C,D), gastric cancer (GSE54129) (Figure 1E,F), pancreatic ductal adenocarcinoma (GSE132956) (Figure 1G,H), hepatocellular carcinoma (GSE164760) (Figure 1I,J), and colorectal adenocarcinoma (GSE110224) (Figure 1K,L) were analyzed to detect the primary molecular mechanisms of malignancies in GI cancers. The heatmap plot shows genes with significant differential expression in these cancers (Figure 1A–L). A heatmap displays the values of a primary variable of interest across two axial variables as a grid of colored squares. The axial variables are divided into ranges like a bar chart or histogram, and the color of each cell indicates the value of the primary variable within the corresponding cell range. On the other hand, we designed the PPI networks of hub genes in the pathogenesis of GI cancers separately (Figure 1A–L). Data analysis of the microarray datasets discovered three master genes with significant differential gene expression, including *STC2*, *SKAP2*, and *SERPINE1*, based on the Venn diagram (Figure 1M).

#### 3.1.2. Pinpointing Key Drivers of Gastrointestinal Malignancy Through Integrated TCGA RNA-Seq Data Analysis

**Figure 2:**
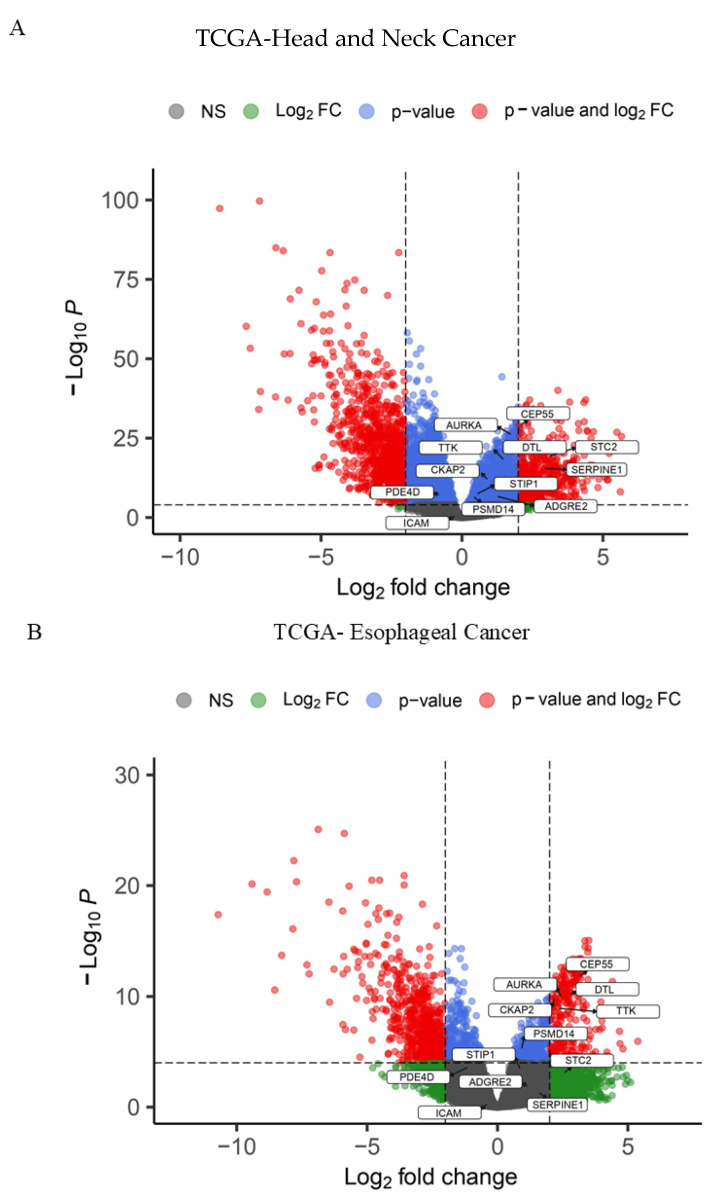

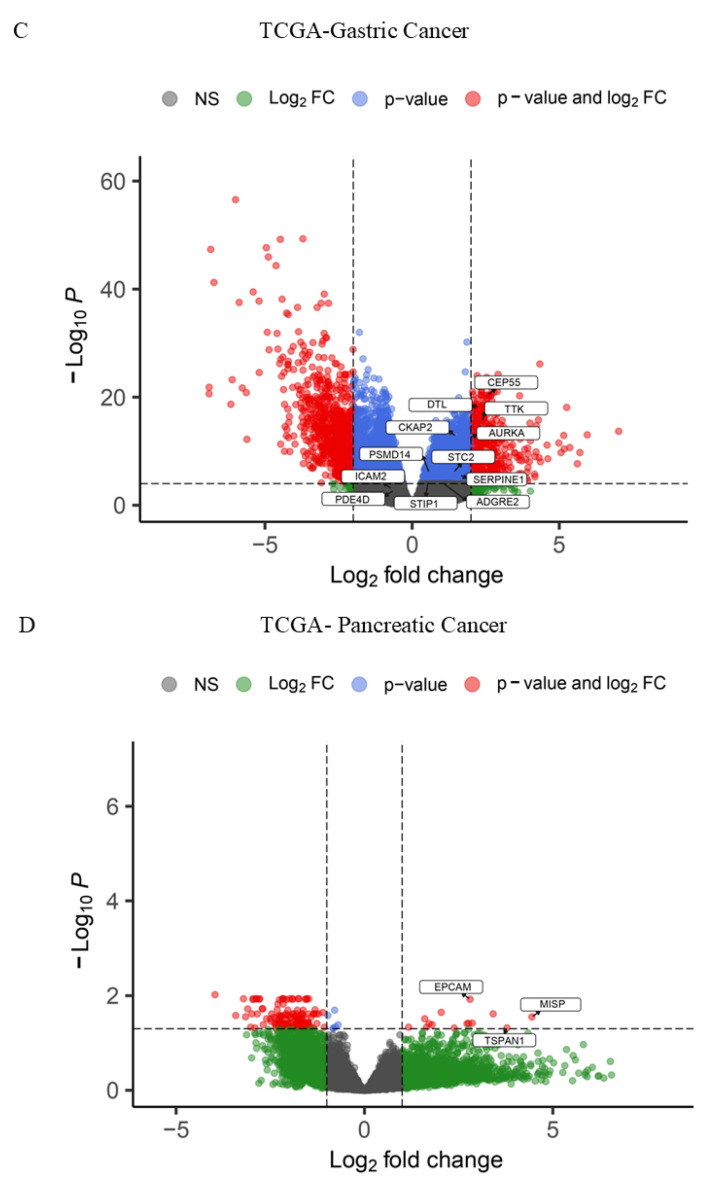

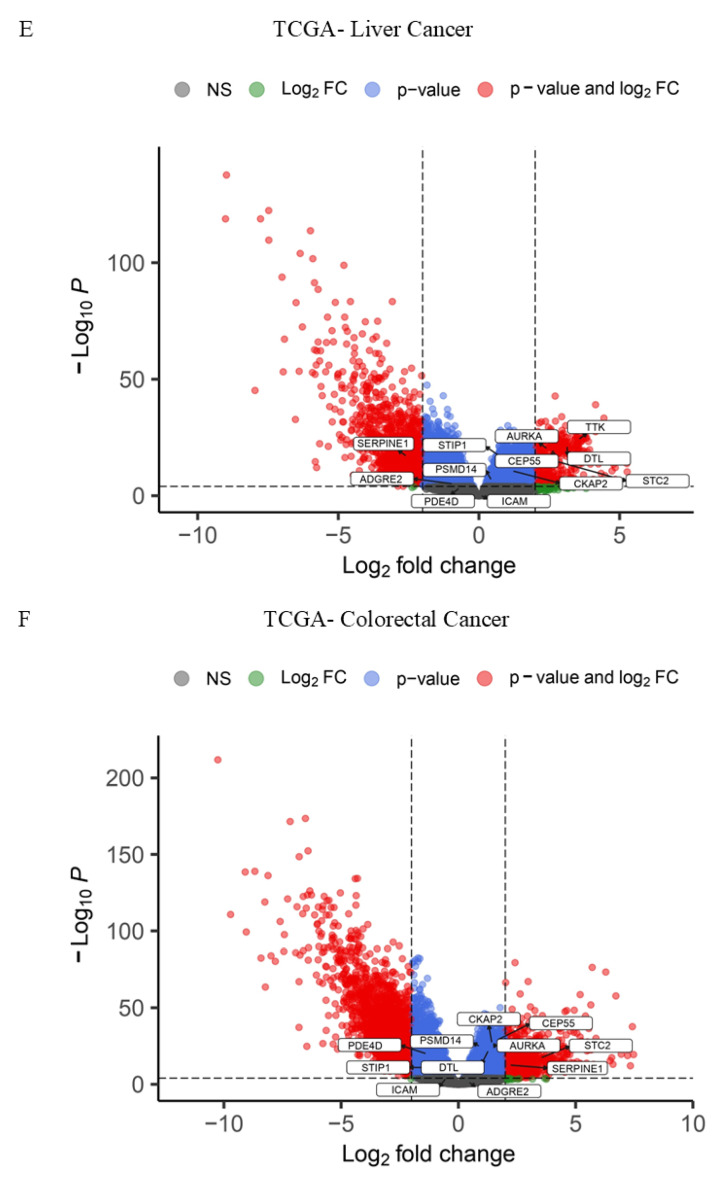
**RNA-Seq platform identification of hub genes:** (**A**–**F**) The volcano plots were applied to visualize the most significant differentially expressed gene (DEG) list in PAN-gastrointestinal cancers based on RNA-Seq data. The graphic considers the logarithmic fold change (logFC) of the genes. The data describe the use of volcano plots to illustrate the differences in gene expression between PAN-GI cancers and normal tissue, using data from The Cancer Genome Atlas (TCGA). Genes were deemed acceptable by evaluating their logFC and adj. *p* value, which helped discover genes that may play a role in tumorigenesis processes. There is a correlation between variations in gene expression patterns and the prognosis of persons with gastrointestinal cancers.

Due to limitations in microarray chips to identify switchable key genes in PAN-gastrointestinal cancers, we were unable to accurately identify the key genes that can be switched on or off in some gastrointestinal cancers; hence, we analyzed RNA-Seq data from the TCGA database to find corresponding switchable master genes in six common types of PAN-gastrointestinal cancers. Volcano plots designed based on RNA-Seq data analysis in the R programming language show the pattern of significant differential gene expressions as master genes (Figure 2A–F).

**Figure 3:**
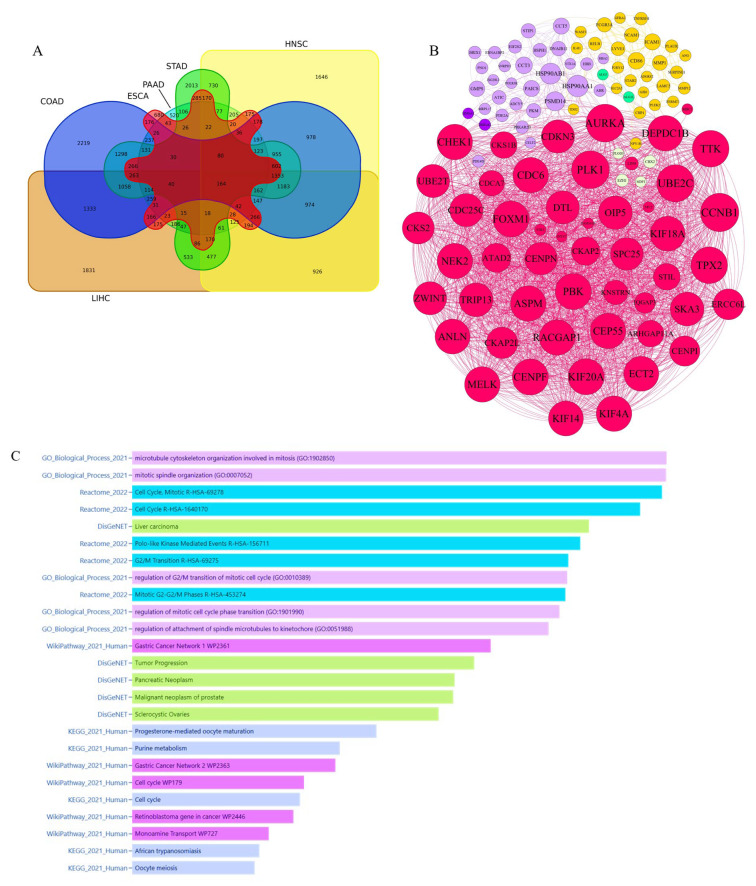
**Cross-platform identification of hub genes based on microarray and RNA-Seq analyses:** (**A**) The Venn diagram bioinformatics tool was employed to identify shared genes that were present in the RNA-Seq datasets. This analysis revealed a total of 164 genes that were common among the important markers, as determined by adj. *p* values. (**B**) The network of interactions between the merged hub genes was generated by employing Gephi software 0.9.2, which applied network parameters, an eigenvector, and a modularity class. Our bioinformatics analysis discovered major biomarkers inside this network that might be important indications for prognosis, diagnosis, and monitoring. (**C**) An enrichment analysis of 167 common hub genes identified through RNA-Seq and microarray aanalyses revealed that the microtubule cytoskeleton organization is involved in mitosis, polo-like kinase-mediated events, G2/M transition, the cell cycle, tumor progression, malignant neoplasms, purine metabolism, retinoblastoma genes in cancer, and monoamine transport. These molecular signaling pathways are predominantly associated with PAN-gastrointestinal cancers’ pathogenesis.

Based on RNA-Seq data analysis of PAN-gastrointestinal cancers, 164 common hub genes were discovered. These common TCGA RNA-Seq data analysis genes were specified based on the Venn diagram (Figure 3A).

It is worth mentioning that, at this point, a thorough literature review evaluation of these hub genes was conducted systematically. Among them, 16 genes were identified as robust markers based on previous studies. An analysis of the available literature, data mining, and hub gene scoring led to the identification of a molecular panel of 16 genes as a discrimination index between cancer tissue and control tissue: *PSMD14*, *STC2*, *PDE4D*, *AURKA*, *LAMC2*, *CEP55*, *RELB*, *ICAM1*, *STIP1*, *DTL*, *TTK*, *ALG1L*, *TDO2*, *ADGRE2*, *CKAP2*, and *SERPINE1*.

#### 3.1.3. Integrative Analysis of Microarray and RNA-Seq Data Reveals Key Pathways and a 16-Gene Biomarker Panel for Gastrointestinal Cancers

Finally, the gene lists from microarrays and RNA-Seq data were merged, and a genetic interaction network was designed (Figure 3B). On the other hand, enrichment analysis of 167 common hub genes revealed that the dominant molecular signaling pathways associated with PAN-gastrointestinal cancers’ pathogenesis include microtubule cytoskeleton organization in mitosis, polo-like kinase-mediated events, G2/M transition, the cell cycle, tumor progression, malignant neoplasms, purine metabolism, retinoblastoma genes in cancer, and monoamine transport (Figure 3C). Based on the literature review, data mining, and scoring of hub genes, a molecular panel including 16 genes—*PSMD14*, *STC2*, *PDE4D*, *AURKA*, *LAMC2*, *CEP55*, *RELB*, *ICAM1*, *STIP1*, *DTL*, *TTK*, *ALG1L*, *TDO2*, *ADGRE2*, *CKAP2*, and *SERPINE1*—with high potential scores was identified (Figure 3B). 

### 3.2. Diagnostic Performance and ROC Curve Analysis

**Figure 4:**
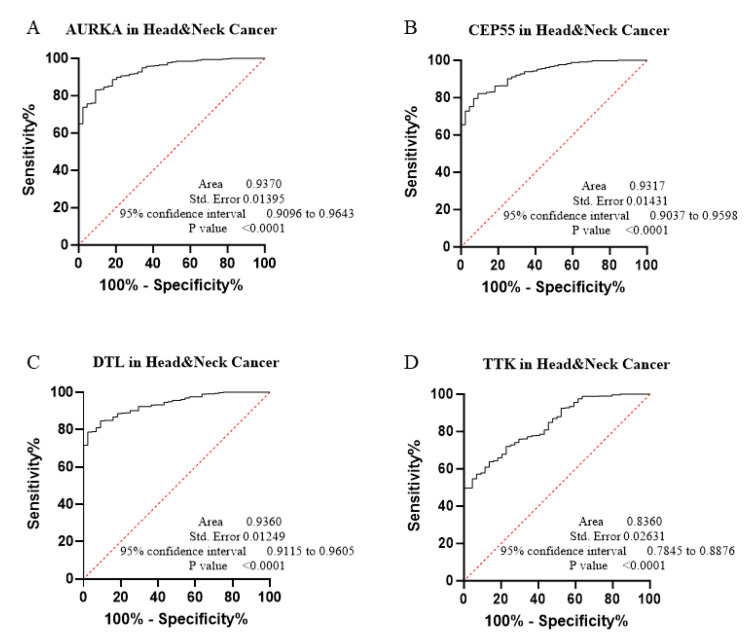
(**A**–**D**) Applying bioinformatics and ROC analysis, we assessed 4 master genes that could possess biomarker properties in HNSC.

**Figure 5:**
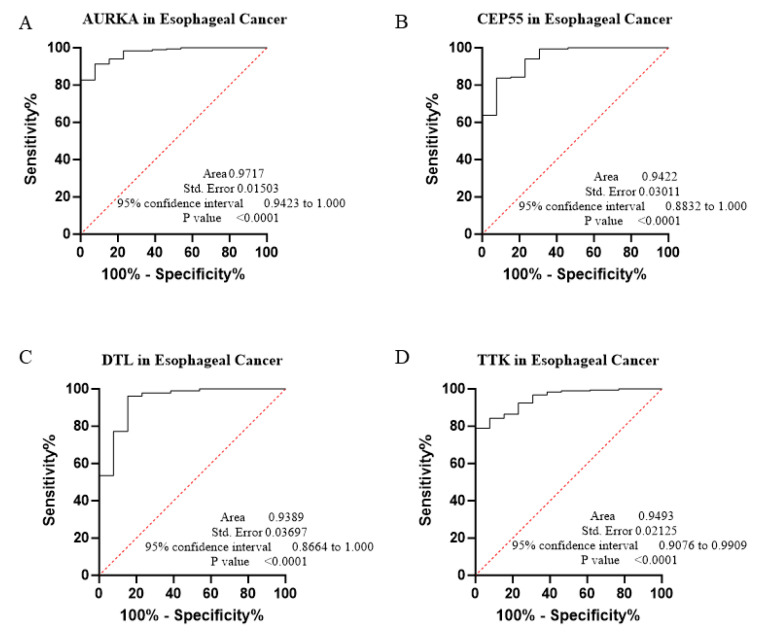
(**A**–**D**) We applied bioinformatics and ROC estimation to evaluate 4 master genes that may exhibit biomarker characteristics in ESCA.

**Figure 6:**
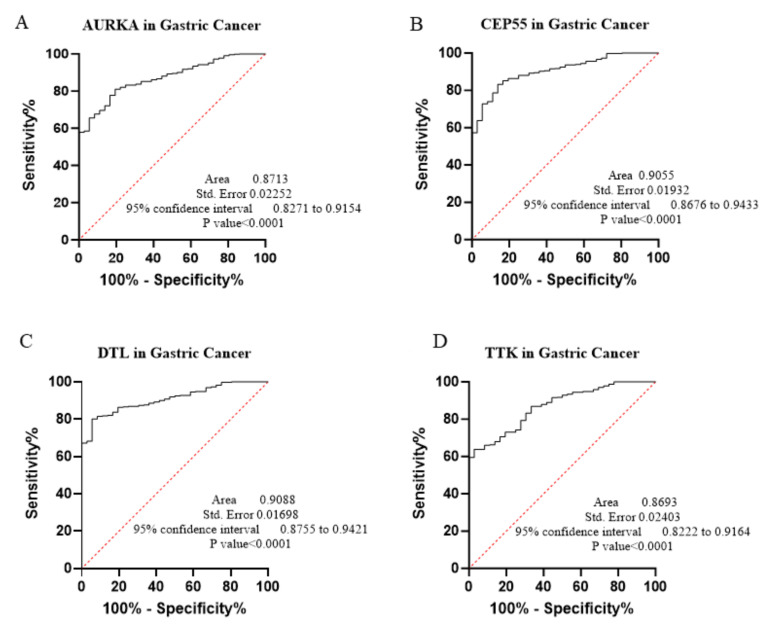
(**A**–**D**) We applied bioinformatics and ROC modeling to assess 4 key genes that may reflect biomarker properties in STAD.

**Figure 7:**
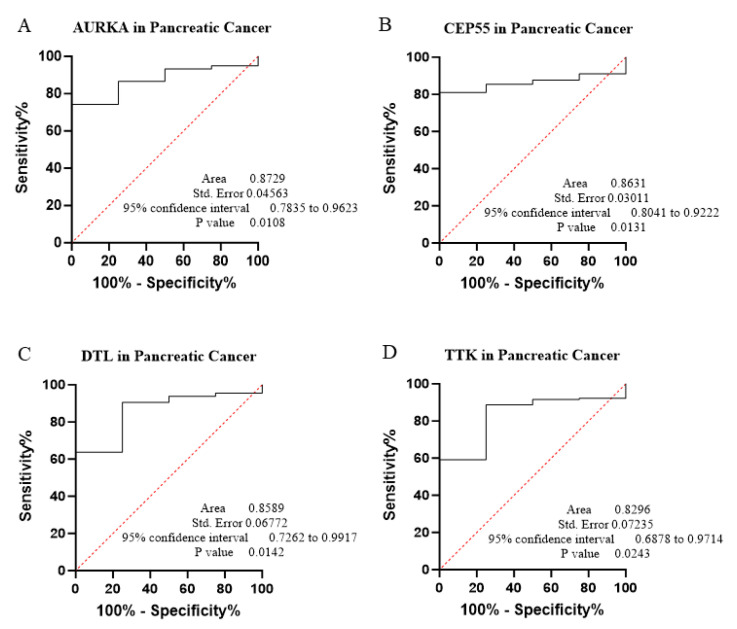
(**A**–**D**) This project applied bioinformatics and ROC statistical methods to evaluate 4 specific genes that may serve as biomarkers in PAAD.

**Figure 8:**
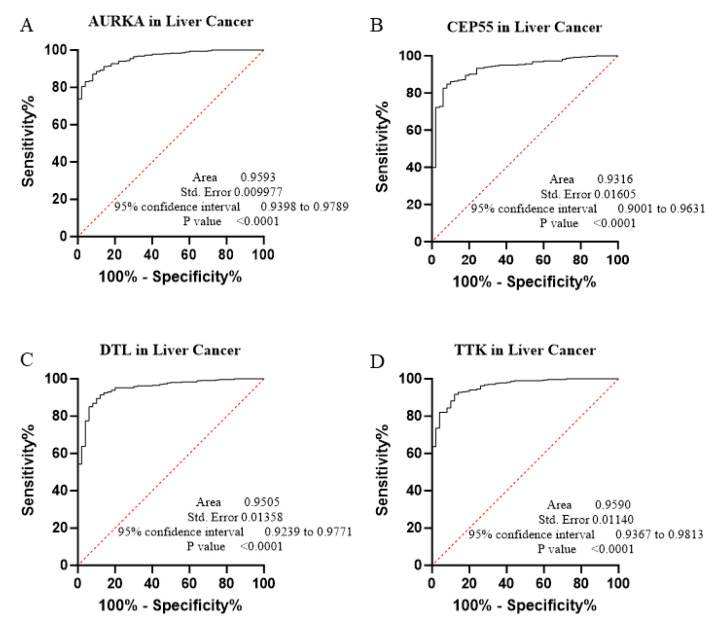
(**A**–**D**) This study implemented bioinformatics and ROC methodologies to assess the potential of 4 particular genes as biomarkers in LIHC.

**Figure 9:**
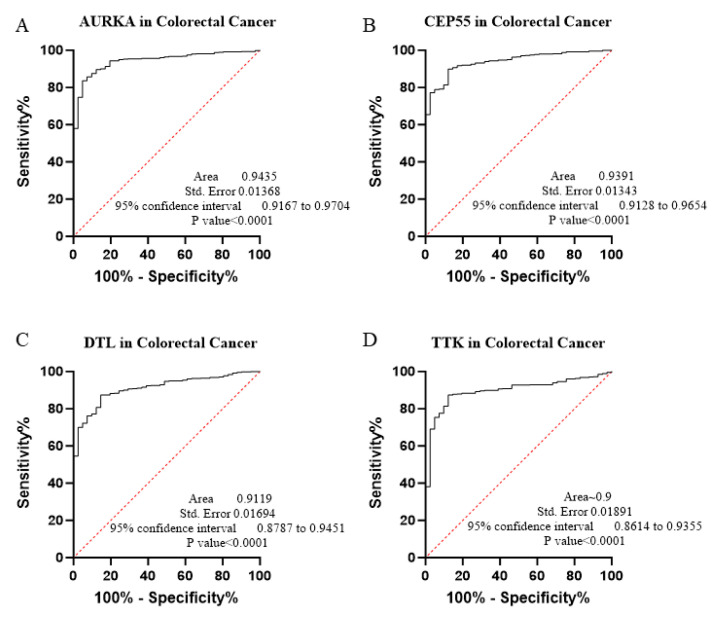
(**A**–**D**) This research applied bioinformatics and the ROC approach to establish the possibility of 4 specific genes as biomarkers in COAD.

**Figure 10:**
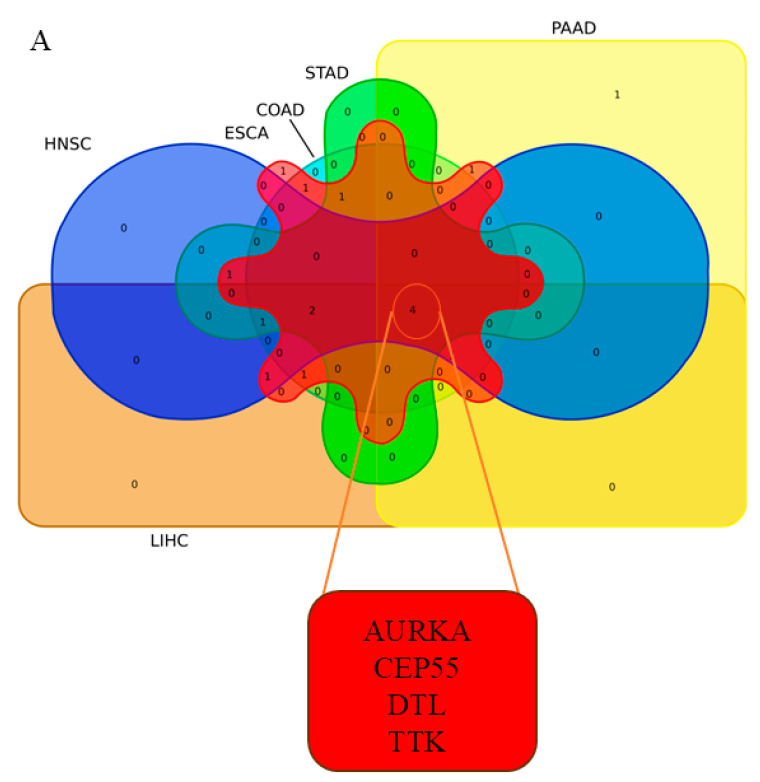

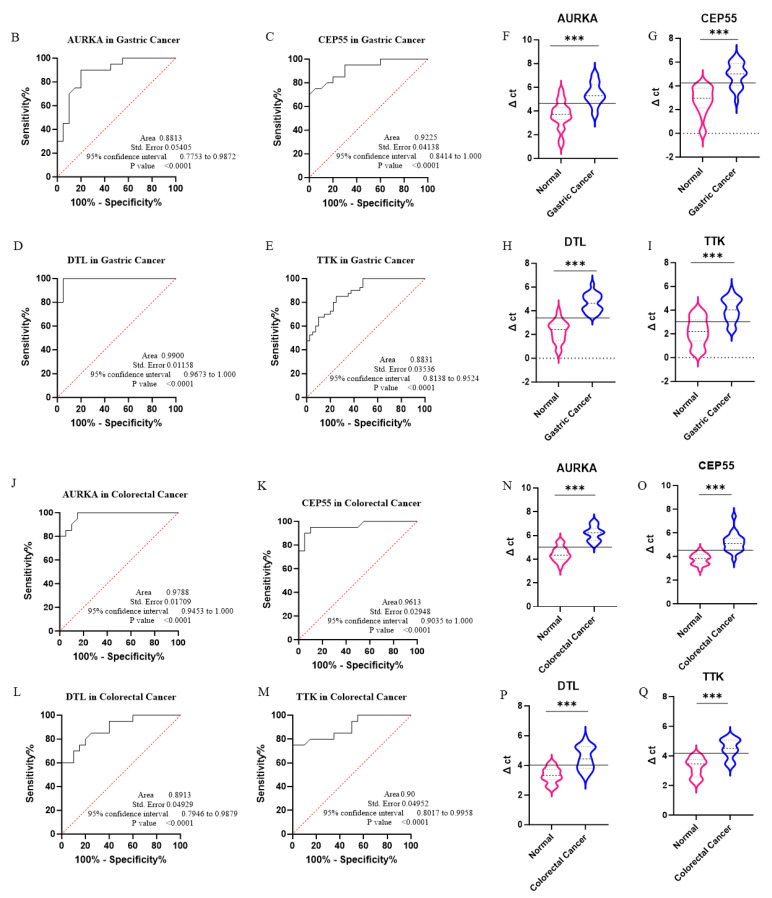
(**A**). The Venn diagram bioinformatics tool was used to discover common genes that were rough across the RNA-Seq and microarray datasets, revealing 4 common genes from significant markers based on the AUC of the ROC curve and the Youden index. (**B**–**Q**) Assessment of the gene expression profiling as biomarkers in gastric and colorectal cancer for cancer screening. Using bioinformatics and ROC analysis, we evaluated four master genes that may have biomarker characteristics. Thus, we chose *AURKA*, *CEP55*, *DTL*, and *TTK* as molecular profiles that may correctly detect colorectal and gastric cancers before symptoms manifest. The ROC analysis revealed that these genes exhibit good diagnostic accuracy, as shown by the AUC values beyond 0.80, suggesting their potential as dependable biomarkers. *** indicated that the *p* value < 0.001.

The core contribution of this project is to introduce the best global biomarker panel for the detection of PAN-GI cancers based on sensitivity and specificity, using the area under the receiver operating characteristic (ROC) curves ≥ 80% and *p* value < 0.05 with 95% confidence interval (CI), along with the Youden score (Figure 4, Figure 5, Figure 6, Figure 7, Figure 8, Figure 9 and Figure 10 and Table 4). The results showed that dysregulation of the genes *PSMD14*, *STC2*, *PDE4D*, *AURKA*, *LAMC2*, *CEP55*, *REALB*, *ICAM1*, *STIP1*, *DTL*, *TTK*, *ALG1L*, *TDO2*, *ADGRE2*, *CKAP2*, and *SERPINE1* had a moderate-to-high effect on distinguishing tumor tissue from healthy tissue, as shown in Figure 4, Figure 5, Figure 6, Figure 7, Figure 8, Figure 9 and Figure 10, Supplementary Figures S1–S12, and Table 4.

The ROC curves in six different forms of gastrointestinal malignancies, as shown in Figure 4, Figure 5, Figure 6, Figure 7, Figure 8, Figure 9 and Figure 10, provide a complete and comprehensive evaluation of gastrointestinal tumor biomarkers. Accordingly, common biomarkers, including *AURKA*, *CEP55*, *DTL,* and *TTK,* were identified as the best biomarkers for diagnosing PAN-gastrointestinal cancers (Figure 10A).

In the next step, in order to confirm the results of the bioinformatics analyses, the expression of the main candidate genes as potential biomarkers at the transcriptional level was evaluated experimentally in the most common gastrointestinal malignancies: gastric and colorectal cancer tissues.

### 3.3. Experimental Validation via qPCR

Based on bioinformatics data and ROC analysis, we evaluated the expression levels of a molecular panel including four major genes—*AURKA*, *CEP55*, *DTL*, and *TTK*—as potential biomarkers using real-time quantitative polymerase chain reaction (qPCR) (Table 5). The violin plot showed that the expression levels of the *AURKA*, *CEP55*, *DTL*, and *TTK* genes were significantly increased in colorectal and gastric tumor tissues (*p* value < 0.05, Figure 10B–I). Also, the sensitivity and specificity of the expression changes of the *AURKA*, *CEP55*, *DTL*, and *TTK* genes for distinguishing colorectal and gastric adenocarcinoma from normal tissue were evaluated by ROC analysis (Figure 10J–N and Table 5). These results indicate that the upregulation of *AURKA*, *CEP55*, *DTL*, and *TTK* in tumor tissues compared to normal tissues is statistically significant, and that these genes can serve as potential diagnostic markers with high accuracy in colorectal and gastric adenocarcinoma (AUC > 0.80).

## 4. Discussion

Despite advances in understanding the cancer-related molecular pathways, PAN-gastrointestinal cancers still remain among the most important causes of cancer-related mortality worldwide, and their frequent occurrence, high recurrence rates, and short survival rates require improved diagnostic and therapeutic approaches [27]. Since the survival outlook of advanced PAN-gastrointestinal cancers is less optimistic compared to early-stage PAN-gastrointestinal cancers, the identification of novel biomarkers for early detection is crucial. In the present study, a genetic interaction network was identified using integrative transcriptomics by integrating microarray and RNA-Seq data of six different types of gastrointestinal tumors from the GEO and TCGA databases, respectively. Also, by enrichment analysis of 167 common hub genes related to PAN-gastrointestinal cancers, and by identifying important molecular pathways involved in the occurrence of these cancers, a molecular panel consisting of the *AURKA*, *CEP55*, *DTL*, and *TTK* genes was constructed by using ROC curve analysis and the Youden index as biomarkers for PAN-gastrointestinal cancers, validated using real-time quantitative PCR (qPCR) in patient tissue samples. The findings demonstrated significant overexpression of these genes in tumor tissue compared to normal tissue (*p* < 0.001). ROC analysis showed that these genes have high sensitivity and specificity (AUC > 0.80) in differentiating tumor tissue from normal tissue, and that they can be used as reliable biomarkers for early cancer diagnosis (Stage I and II) based on experimental validation. Emerging studies have noted that several biological biomarkers, such as programmed death-ligand 1 (*PD-L1*) [28,29,30], mismatch repair deficiency/microsatellite instability (*dMMR/MSI-H*) [31], Rho GTPase-activating protein 26 (*ARHGAP26*) [31], human epidermal growth factor receptor 2 (*HER2*) [32], neurotrophic tropomyosin receptor kinase (*NTRK*) [33], lymphocyte-activation gene 3 (*LAG3*) [34], T-cell immunoglobulin and mucin-domain containing-3 (*TIM3*) [35], claudin-18 (*CLDN18*) [36], and fibroblast growth factor receptor (*FGFR*) [37], can be used to prognose, diagnose, monitor, and manage PAN-gastrointestinal cancers.

*TTK* (threonine tyrosine kinase) is an imperative enzyme in the spindle assembly checkpoint that manages the growth of tumor cells in several species. Numerous studies have shown that *TTK* expression is abnormally increased in various types of human malignancies, such as triple-negative breast cancer, pancreatic cancer, and hepatocellular carcinoma, and contributes to cancer promotion and, in some cases, poor prognosis [38,39,40,41]. However, the expression level of *TTK* and its prognostic significance in PAN-gastrointestinal cancers have not been sufficiently studied. A comprehensive analysis of the present study on gastrointestinal cancer types using multiple databases showed that *TTK* mRNA expression was increased in patients, which was consistent with previous results. Also, these results were experimentally confirmed in gastric and colorectal tumor tissues, and ROC curve analysis emphasized the potential of *TTK* mRNA expression as a diagnostic biomarker to distinguish cancerous tissue from normal tissue. The clinicopathological features and ROC curve analysis of *TTK* expression demonstrated significant associations between *TTK* mRNA expression and both tumor metastasis and high TNM staging of endometrial cancer patients. Furthermore, Du et al. demonstrated a correlation between elevated *TTK* mRNA expression and a worse prognosis, indicating that *TTK* has the potential to serve as a biomarker for unfavorable prognosis in patients with endometrial cancer based on Kaplan–Meier analysis [42]. Recently published studies and our findings provide strong evidence that *TTK* is a biomarker for predicting outcomes in several cancer types [43]. *TTK* gene expression is increased in colon tumor tissues compared to normal tissue, which is associated with increased proliferation of cancer cells through the activation of PKCα/ERK1/2 and inhibition of differentiation through inactivation of the PI3K/Akt pathway [44].

Suppression of *TTK* in pancreatic ductal adenocarcinoma cell lines significantly reduces cell migration and increases cell death by inducing apoptosis, indicating its significant role in pancreatic carcinogenesis [45]. Also, increased expression of *TTK* has been observed in various types of lung cancer, which leads to the stimulation of cell migration and the process of epithelial–mesenchymal transition (EMT), ultimately leading to increased metastatic capabilities and facilitating tumor expansion [46,47]. In addition, increased transcription of *TTK* in non-small-cell lung cancer (NSCLC) is associated with a poorer prognosis [48], and suppression of *TTK* prevents the growth, movement, and tumor formation in NSCLC. In addition, increased expression of *TTK* in hepatocellular carcinoma tissue compared to normal tissue has also been reported. More importantly, functional studies have also confirmed the carcinogenic activity of *TTK*, which is strongly associated with senescence and autophagy in tumor cells [49]. Chen et al., in 2019 [50], showed that the expression of *TTK* at the mRNA and protein levels increases in prostate cancer, and by knockdown of the *TTK* gene, the proliferation, invasion, and migration of cancer cells are inhibited and cell apoptosis is induced. Further studies in vivo have shown that *TTK* knockout inhibits tumorigenesis in mice by inhibiting the expression of *CDK2* and *CCNE1* [50].

*CEP55* (Centrosomal Protein 55) is a key player in cytokinesis, the final step of cell division, and regulates the physical separation of daughter cells. Its role in tumorigenesis has garnered researchers’ attention due to its involvement in various cellular processes that contribute to the development and progression of cancer. Dysfunction or overexpression of *CEP55* can lead to cytokinesis failure. This unsuccessful cytokinesis can lead to chromosomal instability, aneuploidy, the formation of multinucleated cells, and increased probability of oncogenic mutations, and it is associated with tumor formation [51]. Another role of *CEP55* is the regulation of cell proliferation and survival through activating the AKT/PI3K signaling pathway, a critical pathway for cell proliferation and survival. Overexpression of *CEP55* can lead to uncontrolled cell division and survival, contributing to cancer progression. On the other hand, *CEP55* may interact with components of the p53 pathway, which is pivotal for cell-cycle arrest and apoptosis in response to DNA damage. Dysregulation of this interaction can impair normal cellular responses to genomic stress, leading to tumorigenesis. Some studies suggest that *CEP55* is involved in maintaining cancer stem cell properties. Cancer stem cells have the ability to self-renew and give rise to heterogeneous tumor cell populations, contributing to tumor heterogeneity, resistance to therapy, and relapse [52]. The clinical significance of *CEP55* as a potential biomarker is verified with the overexpression of *CEP55*, which has been observed in several cancer types, including breast, colorectal, liver, lung, and ovarian cancers [53,54,55,56]. Its expression levels are often correlated with poor prognosis, increased tumor size, and advanced tumor stage, making it a potential biomarker for cancer diagnosis and prognosis [55]. Inhibitors designed to disrupt its function could potentially limit tumor growth and prevent metastasis. *CEP55* can interact with several other oncogenes and tumor suppressors, including Aurora Kinase A, *PLK1*, and survivin, further modulating its impact on cell division and survival [57,58].

In conclusion, *CEP55* is a multifunctional protein that significantly contributes to tumorigenesis through its roles in cytokinesis, genomic stability, cell proliferation, EMT, and cancer stem cell maintenance. Its dysregulation is commonly associated with aggressive tumor behavior and poor clinical outcomes, making it an attractive target for cancer research and therapeutic intervention. The findings of KEGG and GO enrichment analyses show that *CEP55* is involved in cell division, DNA repair, and apoptosis, and loss of *CEP55* function can lead to fetal death at the end stages of pregnancy and the development of Meckel-like syndromes and MARCH syndromes [52,59,60]. Given that tumors often arise from abnormal differentiation resulting from genomic chromosomal instability during cell division [28,55,61], significant upregulation of *CEP55* is associated with tumor progression, invasion, and reduced survival [55,61,62,63,64]. For example, increased expression of *CEP55* promotes anchorage-free proliferation of hepatocellular carcinoma by stimulating PI3K/AKT activity [65]. Furthermore, upregulated expression of *CEP55* greatly facilitates the progression of endometrial cancer, and conversely, decreased expression of *CEP55* inhibits the proliferation and accelerates cell death [66]. These findings show that *CEP55* could be promising as a viable therapeutic target for cancer treatment. However, relevant studies are limited to a small number of cancer types, and there is currently little understanding of the therapeutic effects and processes of *CEP55* in different tumor types [52,67].

On the other hand, defects in the cytokinesis process during the early stages of tumor proliferation may lead to the appearance of aneuploid cells, which subsequently turn into unstable and potentially cancerous cells. The *CEP55* protein is phosphorylated during mitosis and changes from the centrosome to the midbody in the advanced stages of mitosis. Our findings indicate that elevated levels of *CEP55* have a substantial impact on the pathological stage of tumors in gastric and colorectal malignancies. In the middle-aged group and above (>41 years), patients with BRCA, HNSC, LUAD, PAAD, and UCEC tumors showed a significant increase in the levels of *CEP55*. Using Kaplan–Meier survival analysis, a significant correlation was observed between elevated expression of *CEP55* in ACC, KIRC, PPAD, KIRP, LGG, LIHC, and MESO and worse prognosis in terms of overall survival (OS) and disease-free survival (DFS). Our results expand the range of findings from prior documentation [63,68,69]. Evidence indicates that *CEP55* changes in tumor patients mostly consist of non-synonymous modifications, such as mutations, amplified values, and deep deletions. The prevalence of *CEP55* mutations was highest in uterine endometrial carcinoma (4.91%, 26/529), and then in diffuse large B-cell lymphoma (2.08%, 1/48). Amplification was most common in uterine carcinosarcoma (3.51%, 2/57), followed by uterine endometrial carcinoma (0.76%, 4/529). Deep deletions were most frequent in prostate adenocarcinoma (2.23%, 11/494), followed by diffuse large B-cell lymphoma (2.08%, 1/48). These findings suggest that the relationship between *CEP55* variations and tumor progression should be carefully examined [52]. Likewise, in breast carcinoma, suppressing the expression of *CEP55* by knockdown strategies significantly reduced cell viability, growth, and migration. Conversely, increasing the expression of *CEP55* significantly boosted breast cancer cell proliferation and migration.

Aurora kinase A (*AURKA*) is a serine/threonine kinase that regulates mitotic processes such as centrosome duplication, spindle assembly, and chromosome segregation. Examining the expression level of *AURKA* in different cancers indicates that this gene can act as an oncogene or a tumor suppressor gene [70,71]. Several early studies found that Aurora kinase A is overexpressed at the transcript and protein levels in various malignancies, including leukemia, prostate cancer, esophageal adenocarcinoma, and pancreatic cancer cell lines [72]. Its overexpression is linked to cancer development due to its role in inducing chromosomal instability, genomic instability, and aneuploidy. *AURKA* promotes tumorigenesis by disrupting the spindle assembly checkpoint, shortening the G2 phase, and activating oncogenic pathways, including p53 degradation and MYC stabilization, as well as invasion and metastasis via EMT [73]. Also, Lu H et al. suggested that increased expression of *AURKA* is involved in regulating cell proliferation and progression by affecting the unfolded protein response (UPR) [74]. On the other hand, Marta et al. showed that increased expression of *AURKA* was associated with improved overall survival (OS) and progression-free survival in patients with ovarian cancer (OV) [75], which could be caused by the strong association of *AURKA* with genes related to the regulation of the immune system [72]. Also, *AURKA* overexpression has been reported to be strongly associated with increased survival rates in colon cancer patients [76], while Chuang et al. showed that *AURKA* overexpression plays an important role in colorectal cancer progression [77].

*DTL* (Denticleless E3 Ubiquitin Protein Ligase Homolog), during the S phase and following DNA damage, serves as a switchable regulator of DNA replication, the cell cycle, and the DNA damage response (DDR) by acknowledging and degrading a range of substrates, including the DNA replication permitting factor *CDT1*, the cell-cycle suppressor *P21*, the histone methyltransferase *SET8*, and the checkpoint kinase *CHK1*. Dysregulation of *DTL* leads to uncontrolled DNA replication and cell-cycle progression and genomic instability, which can be followed by cell death or malignant development [78]. *DTL* overexpression has been reported to be associated with poor patient survival in many types of cancer [79,80,81,82,83,84]. Our bioinformatics research showed that *DTL* expression was increased in almost all GI cancers, which was also confirmed in the clinical pathological samples of our project on colorectal and gastric cancer. These results are consistent with the study of Yumei Tang et al., who reported a strong negative relationship between increased *DTL* expression and the survival time and status of patients with urothelial carcinoma [85]. The survival analysis of gastric cancer patients could not be conducted, due to insufficient follow-up data. The results suggest that the expression of *DTL* has the potential to be used as a diagnostic and prognostic indicator for various types of cancer. Given the significance of cancer immunity, ample research has extensively investigated the correlation between the immune status of tumors and the expression of *DTL*. The bioinformatics analysis revealed a strong correlation between the expression of *DTL* and the infiltration of immune cells. *DTL* has the potential to serve as a marker for assessing immune infiltration in various types of cancer. Moreover, the excessive expression of *DTL* is directly associated with the infiltration of CD3^+^T-cells in clinical samples obtained from patients with hepatocellular carcinoma, bladder urothelial carcinoma, and stomach adenocarcinoma. Furthermore, bioinformatics analysis revealed a strong positive correlation between *DTL* expression and the expression of the majority of immunological checkpoint (ICP) genes across all cancer types. Hence, elevated levels of *DTL* expression might serve as an indicator for favorable outcomes in immunotherapy, specifically targeting ICP genes. However, *DTL* inhibitors might serve as a viable alternative therapy. Therefore, the hypothesis is that *DTL* could forecast the immune cell infiltration and the response to immunotherapy in some cancer types [85]. Recent research by Changwu Wu and colleagues has merged multi-omics data from more than 10,000 samples spanning 33 tumor types to create a “cuproptosis activity score” and clarify the role of copper-dependent cell death in cancer [86]. In comparison, our investigation zeroed in on gastrointestinal cancers, employing a blended approach that incorporated differential expression analysis, PPI network construction, and ROC curve analysis to identify essential diagnostic biomarkers such as *AURKA*, *CEP55*, *DTL*, and *TTK*. We should clarify that, while both studies align in integrating transcriptomics datasets from TCGA and GEO, our method focused specifically on tumor-type-related characteristics and experimental validation in gastric and colorectal cancers. By examining the methodologies of the study on cuproptosis regulators, we recognize that including an activity score or functional metric can enhance our understanding of the influence of candidate genes on tumor biology. We should highlight that, although our research concentrated on a specific group of cancers (gastrointestinal malignancies), the pan-cancer methodologies presented in the paper on cuproptosis regulators provide a wider viewpoint. While the cuproptosis research takes a comprehensive look at cell death regulation across various cancer types, our targeted analysis enabled us to validate candidate markers experimentally through qPCR in patient samples, thereby establishing significant clinical relevance for gastrointestinal cancers. We propose that upcoming research might benefit from employing a dual approach, merging our focused experimental validations with expansive pan-cancer data integration methods akin to those used in the cuproptosis investigation to reveal the intricate regulatory networks influencing tumorigenesis. AURKA is an essential serine/threonine kinase that regulates the G2/M transition, centrosome maturation, and spindle assembly. Dysregulation or overexpression of AURKA leads to centrosome amplification and faulty spindle formation, which, in turn, result in chromosomal mis-segregation during mitosis, contributing to aneuploidy—a key characteristic of cancer development. TTK is a critical spindle assembly checkpoint (SAC) kinase that assesses kinetochore–microtubule attachments to ensure accurate chromosome segregation. Overexpression or dysfunction of TTK reduces the accuracy of the SAC, allowing cells with misaligned chromosomes to proceed through mitosis. This situation significantly heightens genomic instability and encourages malignant transformation. Our enrichment analysis, which emphasizes the G2/M transition and relevant pathways, indicated that the dysregulation of these kinases directly drives chromosomal instability in GI cancers.

In addition to its role in mitotic control, *AURKA* influences the tumor microenvironment by affecting pathways essential for EMT—a critical process for cancer invasion and metastasis. Increased *AURKA* activity has been linked to changes in cytoskeletal dynamics and cell–cell adhesion factors, facilitating the transition from an epithelial to a mesenchymal phenotype. This shift not only enhances cellular motility but also contributes to apoptosis resistance, characteristics central to aggressive tumor behavior. Likewise, dysregulation of *TTK* is associated with the strengthening of EMT markers, suggesting that both kinases work together to enhance metastatic potential in GI malignancies. The KEGG and GO enrichment analyses (Figure 3C) highlight pathways related to cell-cycle regulation, including the G2/M transition and microtubule cytoskeleton organization. Mechanistically, disruptions in these pathways can impede normal mitosis and promote the buildup of genetic aberrations. Our study discusses how the overexpression of *AURKA* and *TTK* may drive rapid cell proliferation while facilitating the structural and functional changes (e.g., centrosome dysfunction, spindle anomalies) underpinning chromosomal instability and EMT. These changes foster a cellular environment that is conducive to tumor initiation and progression across various GI cancer types. By integrating these mechanistic insights, our manuscript presents a more thorough explanation of how dysregulation of *AURKA* and *TTK* interacts with critical cell-cycle and EMT processes to enhance GI carcinogenesis, linking the enriched pathways directly to our biomarker panel. We believe that this expanded discussion elucidates the biological relevance of our findings and supports the future targeting of these kinases in diagnostic and therapeutic approaches.

The present research introduces a novel gene panel that differentiates malignant tissues from normal tissues in various gastrointestinal cancers, with high diagnostic accuracy (AUC > 0.80), achieved by integrating extensive transcriptomics data from public repositories with stringent quantitative PCR validation, especially in gastric and colorectal samples. This method differs from traditional protein-based biomarkers such as PD-L1, which is evaluated using immunohistochemistry utilizing the combined positive score and is hindered by inter-observer variability and restricted early diagnostic sensitivity. This method differs from HER2, which is expressed in a limited patient population and mostly informs targeted therapy. Although commercial assays such as Oncotype DX GI offer standardized multiparametric evaluations, they tend to be costly and time-intensive. Consequently, our transcript-derived panel presents a promising, rapid, and economical alternative for early cancer detection and personalized treatment, albeit requiring further validation in additional gastrointestinal malignancies and larger cohorts.

## 5. Limitations and Future Perspectives

Despite the significant findings of this study, several limitations should be noted. Firstly, the integrated transcriptomics approach, while powerful, is dependent on the quality and consistency of the data from publicly available databases like TCGA and GEO. Variability in sample collection, processing, and data annotation across different studies might introduce biases, potentially affecting the reproducibility of the results. Additionally, this study’s focus on transcriptomics data does not account for other layers of biological regulation, such as proteomics, epigenomics, and metabolomics, which could provide a more comprehensive understanding of the molecular mechanisms driving PAN-gastrointestinal cancers. Our research emphasized early-stage gastric and colorectal cancer specimens to mitigate some factors, recognizing that more studies with bigger and more heterogeneous cohorts are essential to comprehensively resolve these concerns. Our suggested method for clinical adoption focuses on RT-qPCR, given its superior sensitivity, quantitative results, and proven use in molecular diagnostics. RT-qPCR provides a reliable preliminary screening method for evaluating the gene expression levels of our potential biomarkers. We acknowledge that immunohistochemistry (IHC) is a commonly used instrument in clinical practice for the validation of tissue-based biomarkers. However, it should be highlighted that we encountered constraints in the use of IHC assays in our present investigation. These constraints prevented us from directly connecting our qPCR results with IHC expression patterns at this time. Thus, our research utilized RT-qPCR as the principal technique for technical confirmation. For future clinical application, we propose a dual-platform process in which RT-qPCR functions as the primary screening method. Upon the completion of further validation efforts to rectify our existing shortcomings with IHC—specifically via the creation and standardization of reliable antibody reagents and protocols—complementary IHC analysis may be used to facilitate in situ validation of the biomarker panel. This integrated methodology will improve cancer detection by merging the quantitative accuracy of RT-qPCR with the geographical and morphological insights provided by IHC. Another limitation is the relatively small number of biomarkers validated for clinical application. While the identified panel of 16 genes showed promise, this study did not explore the full spectrum of potential biomarkers across all PAN-gastrointestinal cancer subtypes, nor did it extensively validate these markers in diverse populations. This could limit the generalizability of the findings. Furthermore, this study primarily focused on early diagnosis and did not extensively explore the prognostic or therapeutic implications of these biomarkers, which are equally crucial for improving patient outcomes. Future studies should address these limitations by integrating multi-omics approaches, including proteomics, epigenomics, and metabolomics, to provide a more holistic view of PAN-gastrointestinal cancers’ biology. This would help in identifying additional biomarkers that could improve diagnostic accuracy and potentially uncover new therapeutic targets. Furthermore, expanding the validation of the identified biomarkers in larger, independent cohorts, including those from diverse ethnic and geographic backgrounds, is essential to enhance the generalizability of the findings. We recognize that the integration of multi-omics information from public archives such as TCGA and GEO may originate from disparate research, platforms, and batches, potentially introducing undesirable variances. Despite the use of normalizing techniques like the TMM (trimmed mean of M-values) method in conjunction with the Voom transformation inside the Limma package to mitigate technical variability, residual batch effects may still affect our findings. We emphasize methodologies such as Combat, which have effectively addressed batch-to-batch variability in integrated transcriptomic analyses, and we propose that subsequent iterations of our study may integrate these specialized batch correction pipelines to further validate the robustness of our findings. Furthermore, we should underscore that during the data integration pipeline, stringent quality control filtering was executed, and genes with low expression levels (assessed by CPM measures) were eliminated to mitigate the influence of technical noise. This further background elucidates that, while batch effects are a fundamental problem, our data processing pipeline was designed to mitigate their impact. The current qPCR validation included 21 patient samples (gastric and colorectal malignancies), constituting a pilot validation cohort. Despite the constrained sample size, our findings were corroborated by comprehensive bioinformatics analysis conducted on extensive public databases. Each experiment was conducted in technical duplicates, and stringent quality control techniques were used (e.g., stability evaluation of the housekeeping gene and incorporation of negative controls) to guarantee the data’s dependability. We advocate for collaborative multi-center research to include more gastrointestinal cancer types (e.g., hepatocellular carcinoma and esophageal cancer) and to validate our preliminary results in larger, stage-stratified cohorts. This will eventually augment the statistical power and clinical significance of our biomarker panel.

In addition, future research should explore the potential of these biomarkers for prognostic and therapeutic applications. Investigating how these biomarkers correlate with patient outcomes, treatment responses, and resistance mechanisms could pave the way for more personalized approaches to PAN-gastrointestinal cancers’ management. Lastly, given the emerging role of non-coding RNAs, immune infiltration, and the tumor microenvironment in cancer progression, integrating these aspects into biomarker discovery and validation could further enhance the predictive power and clinical utility of these biomarkers in PAN-gastrointestinal cancers.

## 6. Conclusions

In conclusion, this study marks a significant step forward in the early diagnosis of PAN-gastrointestinal cancers through the identification and validation of a universal biomarker panel using integrated transcriptomics. With AUC values exceeding 0.80, these biomarkers present a promising approach for improving early cancer detection and enhancing diagnostic accuracy, ultimately contributing to better patient outcomes in PAN-gastrointestinal cancers.

## Figures and Tables

**Table 1:** Details on six prevalent PAN-gastrointestinal cancers derived from the TCGA and GEO databases.

	HNSC *		ESCA *		STAD *		PAAD *		LIHC *		COAD *	
**Quantity of specimens**	Normal	Cancer	Normal	Cancer	Normal	Cancer	Normal	Cancer	Normal	Cancer	Normal	Cancer
**GEO**	12	6	16	16	20	30	5	10	6	53	14	14
**TCGA**	32	297	13	185	36	412	4	179	51	373	41	483

* HNSC: head and neck squamous-cell carcinoma, ESCA: esophageal carcinoma, STAD: stomach adenocarcinoma, PAAD: pancreatic adenocarcinoma, LIHC: liver hepatocellular carcinoma, COAD: colorectal adenocarcinoma.

**Table 2:** Clinical information on individuals with colorectal cancer (CRC) and gastric cancer (GC) referred to Isfahan medical centers.

Characteristic	Colorectal Adenocarcinoma (n = 20)	Gastric Adenocarcinoma (n = 20)	*p* Value
**Age**			
<50	4 (19.0%)	3 (14.3%)	0.693
≥50	17 (81.0%)	18 (85.7%)	
**Gender**			
Male	15 (71.4%)	12 (57.1%)	0.476
Female	6 (28.6%)	9 (42.9%)	
**TNM Stage**			
Stage I	11 (52.4%)	10 (47.6%)	0.827
Stage II	10 (47.6%)	11 (52.4%)	
**Tumor Size (cm)**			
<5			NA

**Table 3:** The sequence of primers used in the study.

Gene	Sequence of Primers	Annealing Temperature
*AURKA*	Forward: 5′-GCAACCAGTGTACCTCATCCTG-3′	60 °C
	Reverse: 5′-AAGTCTTCCAAAGCCCACTGCC-3′	
*CEP55*	Forward: 5′-TCGACCGTCAACATGTGCAGCA-3′	60 °C
	Reverse: 5′- GGCTCTGTGATGGCAAACTCATG-3′	
*DTL*	Forward: 5′-CCAGCCTTAGTCCAGATGACCA-3′	60 °C
	Reverse: 5′-GAGAATGACCCAGGAGCACAGT-3′	
*TTK*	Forward: 5′-CCGAGATTTGGTTGTGCCTGGA-3′	60 °C
	Reverse: 5′-CATCTGACACCAGAGGTTCCTTG-3′	
*B2M*	Forward: 5′-CCACTGAAAAAGATGAGTATGCCT-3′	60 °C
	Reverse: 5′-CCAATCCAAATGCGGCATCTTCA-3′	

**Table 4:** Diagnostic performance metrics of candidate biomarkers in PAN-GI cancers.

Gene	Cancer Type	AUC *	95% CI **	*p* Value	Sensitivity (%)	Specificity (%)	Cut-Off Value
*AURKA*	HNSC	0.9370	0.9096–0.9643	<0.0001	83.14	90.91	4.266
	ESCA	0.9717	0.9423–1.000	<0.0001	91.35	92.31	3.991
	STAD	0.8713	0.8271–0.9154	<0.0001	81.07	80.56	4.312
	PAAD	0.8729	0.7835–0.9623	<0.0108	74.30	100.00	3.008
	LIHC	0.9593	0.9398–0.9789	<0.0001	78.34	84.00	7.474
	COAD	0.9435	0.9167–0.9704	<0.0001	83.64	95.12	5.010
*CEP55*	HNSC	0.9317	0.9037–0.9598	<0.0001	82.18	90.91	4.851
	ESCA	0.9422	0.8832–1.000	<0.0001	83.78	92.31	4.622
	STAD	0.9055	0.8676–0.9433	<0.0001	83.25	86.11	4.230
	PAAD	0.8631	0.8041–0.9222	<0.0131	81.01	100.00	2.952
	LIHC	0.9316	0.9001–0.9631	<0.0001	84.76	92.00	0.8585
	COAD	0.9391	0.9128–0.9654	<0.0001	89.86	87.80	4.374
*DTL*	HNSC	0.9360	0.9115–0.9605	<0.0001	78.74	97.73	4.068
	ESCA	0.9389	0.8664–1.000	<0.0001	96.22	84.62	3.420
	STAD	0.9088	0.8755–0.9421	<0.0001	80.10	94.44	3.701
	PAAD	0.8589	0.7262–0.9917	<0.0142	81.01	100.00	2.952
	LIHC	0.9505	0.9239–0.9771	<0.0001	78.34	84.00	7.474
	COAD	0.9119	0.8787–0.9451	<0.0001	87.37	85.37	3.943
*TTK*	HNSC	0.8360	0.7845–0.8876	<0.0001	57.09	93.18	4.303
	ESCA	0.9493	0.9076–0.9909	<0.0001	78.92	100.00	4.173
	STAD	0.8693	0.8222–0.9164	<0.0001	63.83	97.22	3.984
	PAAD	0.8296	0.6878–0.9714	<0.0243	88.83	75.00	1.444
	LIHC	0.9590	0.9367–0.9813	<0.0001	91.71	88.00	0.7411
	COAD	0.9000	0.8614–0.9355	<0.0001	87.37	87.80	3.773

* AUC: area under the ROC curve, indicating overall diagnostic accuracy; ** 95% CI: 95% confidence interval for the AUC; *p* value: significance level of the ROC analysis; Sensitivity: ability to correctly identify cancer cases; Specificity: ability to correctly identify non-cancer (normal) cases; Cut-Off Value: the threshold used for the diagnostic test.

**Table 5:** Diagnostic performance metrics of candidate biomarkers in colorectal and gastric adenocarcinoma.

Gene	Cancer Type	AUC *	95% CI **	*p* Value	Youden Index Cut-Off
*AURKA*	Colorectal AD	0.9788	0.9453–1.000	<0.0001	≥5.055
	Gastric AD	0.8813	0.7753–0.9872	0.0001	≥4.355
*CEP55*	Colorectal AD	0.9613	0.9035–1.000	<0.0001	≥4.305
	Gastric AD	0.9225	0.8414–1.000	<0.0001	≥4.100
*DTL*	Colorectal AD	0.8913	0.7946–0.9879	<0.0001	≥3.705
	Gastric AD	0.9900	0.9673–1.000	<0.0001	≥3.525
*TTK*	Colorectal AD	~0.90	0.8017–0.9956	<0.0001	≥4.140
	Gastric AD	0.8831	0.8138–0.9524	0.0001	≥3.074

* AUC (area under the curve): indicates the overall diagnostic accuracy; values closer to 1.0 represent higher accuracy; ** 95% CI (confidence interval): provides the range within which the true AUC value is expected to lie with 95% confidence; *p* value: shows the statistical significance of the ROC analysis; Youden Index Cut-Off: the threshold value that maximizes the sum of sensitivity and specificity for distinguishing cancer from normal tissue.

## Data Availability

All of the raw data and the rest of the materials remain at the Islamic Azad University—Shahrekord Branch and are available upon request.

## Supplementary Materials

The following supporting information can be downloaded at https://www.mdpi.com/article/10.3390/biology14070803/s1, Figure S1–S6: The RNA-seq analysis of PAN GI-cancer transcriptomics data indicates a significant increase in the expression of PSMD14, STC2, PDE4D, AURKA, LAMC2, CEP55, RELB, ICAM1, STIP1, DTL, TTK, ALG1L, TDO2, ADGRE2, CKAP2, and SERPINE1 in the malignant samples. Figure S7–S12: This study implemented bioinformatics and ROC methodology to assess the potential of 16 particular genes as biomarkers in PAN GI-cancers.

## References

[1] Chattopadhyay M., Gupta S. (2024). Drugs Affecting Gastrointestinal Functions. Essentials of Pharmacodynamics and Drug Action.

[2] Moravveji S.S., Khoshbakht S., Mokhtari M., Salimi M., Masoudi-Nejad A. (2022). Pan-cancer analysis of biological events on cell cycle instability in gastrointestinal cancers with integrative scoring method. Genomics.

[3] Liu Y., Sethi N.S., Hinoue T., Schneider B.G., Cherniack A.D., Sanchez-Vega F., Seoane J.A., Farshidfar F., Bowlby R., Islam M. (2018). Comparative molecular analysis of gastrointestinal adenocarcinomas. Cancer Cell.

[4] Berger A.C., Korkut A., Kanchi R.S., Hegde A.M., Lenoir W., Liu W., Liu Y., Fan H., Shen H., Ravikumar V. (2018). A comprehensive pan-cancer molecular study of gynecologic and breast cancers. Cancer Cell.

[5] Siegel R.L., Miller K.D., Jemal A. (2019). Cancer statistics, 2019. CA Cancer J. Clin..

[6] Bijlsma M.F., Sadanandam A., Tan P., Vermeulen L. (2017). Molecular subtypes in cancers of the gastrointestinal tract. Nat. Rev. Gastroenterol. Hepatol..

[7] Adam R.S., Blomberg I., Ten Hoorn S., Bijlsma M.F., Vermeulen L. (2021). The recurring features of molecular subtypes in distinct gastrointestinal malignancies—A systematic review. Crit. Rev. Oncol./Hematol..

[8] Ning Y., Lin K., Fang J., Ding Y., Zhang Z., Chen X., Zhao Q., Wang H., Wang F. (2023). Gastrointestinal pan-cancer landscape of tumor matrix heterogeneity identifies biologically distinct matrix stiffness subtypes predicting prognosis and chemotherapy efficacy. Comput. Struct. Biotechnol. J..

[9] Salem M.E., Puccini A., Xiu J., Raghavan D., Lenz H.J., Korn W.M., Shields A.F., Philip P.A., Marshall J.L., Goldberg R.M. (2018). Comparative molecular analyses of esophageal squamous cell carcinoma, esophageal adenocarcinoma, and gastric adenocarcinoma. Oncologist.

[10] Chakravarthy A., Furness A., Joshi K., Ghorani E., Ford K., Ward M.J., King E.V., Lechner M., Marafioti T., Quezada S.A. (2018). Pan-cancer deconvolution of tumour composition using DNA methylation. Nat. Commun..

[11] Yang Y., Zhang J., Chen Y., Xu R., Zhao Q., Guo W. (2020). MUC4, MUC16, and TTN genes mutation correlated with prognosis, and predicted tumor mutation burden and immunotherapy efficacy in gastric cancer and pan-cancer. Clin. Transl. Med..

[12] Cui Y., Guo W., Li Y., Shi J., Ma S., Guan F. (2021). Pan-cancer analysis identifies ESM1 as a novel oncogene for esophageal cancer. Esophagus.

[13] Li L., Zhang Y., Ren Y., Cheng Z., Zhang Y., Wang X., Zhao H., Lu H. (2022). Pan-cancer single-cell analysis reveals the core factors and pathway in specific cancer stem cells of upper gastrointestinal cancer. Front. Bioeng. Biotechnol..

[14] Majeed A., Mukhtar S. (2023). Protein–Protein Interaction Network Exploration Using Cytoscape. Protein-Protein Interactions: Methods and Protocols.

[15] Majeed S., Uzair M., Qamar U., Farooq A. Social Network Analysis Visualization Tools: A Comparative Review. Proceedings of the 2020 IEEE 23rd International Multitopic Conference (INMIC).

[16] Evangelista J.E., Xie Z., Marino G.B., Nguyen N., Clarke D.J., Ma’ayan A. (2023). Enrichr-KG: Bridging enrichment analysis across multiple libraries. Nucleic Acids Res..

[17] Mohamadynejad P., Moghanibashi M., Bagheri K. (2024). Identification of novel nuclear pore complex associated proteins in esophageal carcinoma by an integrated bioinformatics analysis. J. Biomol. Struct. Dyn..

[18] Khashei Varnamkhasti K., Moghanibashi M., Naeimi S. (2023). Genes whose expressions in the primary lung squamous cell carcinoma are able to accurately predict the progression of metastasis through lymphatic system, inferred from a bioinformatics analyses. Sci. Rep..

[19] Colaprico A., Silva T.C., Olsen C., Garofano L., Cava C., Garolini D., Sabedot T.S., Malta T.M., Pagnotta S.M., Castiglioni I. (2016). TCGAbiolinks: An R/Bioconductor package for integrative analysis of TCGA data. Nucleic Acids Res..

[20] Alipour M., Moghanibashi M., Naeimi S., Mohamadynejad P. (2025). LINC00894, YEATS2-AS1, and SUGP2 genes as novel biomarkers for N0 status of lung adenocarcinoma. Sci. Rep..

[21] Chen Y., McCarthy D., Robinson M., Smyth G.K. (2014). edgeR: Differential expression analysis of digital gene expression data User’s Guide. Bioconductor User’s Guide.

[22] Pereira M.B., Wallroth M., Jonsson V., Kristiansson E. (2018). Comparison of normalization methods for the analysis of metagenomic gene abundance data. BMC Genom..

[23] Khashei Varnamkhasti K., Moghanibashi M., Naeimi S. (2024). Implications of ZNF334 gene in lymph node metastasis of lung SCC: Potential bypassing of cellular senescence. J. Transl. Med..

[24] Oliveros J.C., VENNY (2007). An Interactive Tool for Comparing Lists with Venn Diagrams. http://bioinfogp.cnb.csic.es/tools/venny/index.html.

[25] Doncheva N.T., Morris J.H., Gorodkin J., Jensen L.J. (2018). Cytoscape StringApp: Network analysis and visualization of proteomics data. J. Proteome Res..

[26] Kang H. (2021). Sample size determination and power analysis using the G* Power software. J. Educ. Eval. Health Prof..

[27] Zhang C., Shen Q., Gao M., Li J., Pang B. (2024). The role of Cyclin Dependent Kinase Inhibitor 3 (CDKN3) in promoting human tumors: Literature review and pan-cancer analysis. Heliyon.

[28] Petralia F., Ma W., Yaron T.M., Caruso F.P., Tignor N., Wang J.M., Charytonowicz D., Johnson J.L., Huntsman E.M., Marino G.B. (2024). Pan-cancer proteogenomics characterization of tumor immunity. Cell.

[29] Kaviani E., Hosseini A., Mahmoudi Maymand E., Ramzi M., Ghaderi A., Ramezani A. (2022). Triggering of lymphocytes by CD28, 4-1BB, and PD-1 checkpoints to enhance the immune response capacities. PLoS ONE.

[30] Dai L., Huang Z., Li W. (2021). Analysis of the PD-1 ligands among gastrointestinal cancer patients: Focus on cancer immunity. Front. Oncol..

[31] Dhakras P., Uboha N., Horner V., Reinig E., Matkowskyj K.A. (2020). Gastrointestinal cancers: Current biomarkers in esophageal and gastric adenocarcinoma. Transl. Gastroenterol. Hepatol..

[32] Li Z., Chen S., Feng W., Luo Y., Lai H., Li Q., Xiu B., Li Y., Li Y., Huang S. (2020). A pan-cancer analysis of HER2 index revealed transcriptional pattern for precise selection of HER2-targeted therapy. eBioMedicine.

[33] Westphalen C., Preinfalk A., Kruger S., Haas M., Renz B., Riener M.-O., Weber A., Kirchner T., Werner J., Heinemann V. (2019). Neurotrophic tropomyosin receptor kinase (NTRK) and nerve growth factor (NGF) are not expressed in Caucasian patients with biliary tract cancers: Pooled data from three independent cohorts. Clin. Transl. Oncol..

[34] Saleh R.R., Peinado P., Fuentes-Antrás J., Pérez-Segura P., Pandiella A., Amir E., Ocaña A. (2019). Prognostic value of lymphocyte-activation gene 3 (LAG3) in cancer: A meta-analysis. Front. Oncol..

[35] Wang Z., Yin N., Zhang Z., Zhang Y., Zhang G., Chen W. (2017). Upregulation of T-cell immunoglobulin and mucin-domain containing-3 (Tim-3) in monocytes/macrophages associates with gastric cancer progression. Immunol. Investig..

[36] Zhang J., Dong R., Shen L. (2020). Evaluation and reflection on claudin 18.2 targeting therapy in advanced gastric cancer. Chin. J. Cancer Res..

[37] Babina I.S., Turner N.C. (2017). Advances and challenges in targeting FGFR signalling in cancer. Nat. Rev. Cancer.

[38] Elsner J., Cashion D., Robinson D., Bahmanyar S., Tehrani L., Fultz K.E., Narla R.K., Peng X., Tran T., Apuy J. (2021). Structure-guided optimization provides a series of TTK protein inhibitors with Potent Antitumor Activity. J. Med. Chem..

[39] Chandler B.C., Moubadder L., Ritter C.L., Liu M., Cameron M., Wilder-Romans K., Zhang A., Pesch A.M., Michmerhuizen A.R., Hirsh N. (2020). TTK inhibition radiosensitizes basal-like breast cancer through impaired homologous recombination. J. Clin. Investig..

[40] Chan C.Y.-K., Chiu D.K.-C., Yuen V.W.-H., Law C.-T., Wong B.P.-Y., Thu K.L., Cescon D.W., Soria-Bretones I., Cheu J.W.-S., Lee D. (2022). CFI-402257, a TTK inhibitor, effectively suppresses hepatocellular carcinoma. Proc. Natl. Acad. Sci. USA.

[41] Maia A.R.R., Linder S., Song J.-Y., Vaarting C., Boon U., Pritchard C.E., Velds A., Huijbers I.J., Van Tellingen O., Jonkers J. (2018). Mps1 inhibitors synergise with low doses of taxanes in promoting tumour cell death by enhancement of errors in cell division. Br. J. Cancer.

[42] Du H., Zhang L., Chen J., Chen X., Qiang R., Ding X., Wang Y., Yang X. (2024). Upregulation of TTK expression is associated with poor prognosis and immune infiltration in endometrial cancer patients. Cancer Cell Int..

[43] Xie Y., Wang A., Lin J., Wu L., Zhang H., Yang X., Wan X., Miao R., Sang X., Zhao H. (2017). Mps1/TTK: A novel target and biomarker for cancer. J. Drug Target..

[44] Zhang L., Jiang B., Zhu N., Tao M., Jun Y., Chen X., Wang Q., Luo C. (2020). Mitotic checkpoint kinase Mps1/TTK predicts prognosis of colon cancer patients and regulates tumor proliferation and differentiation via PKCα/ERK1/2 and PI3K/Akt pathway. Med. Oncol..

[45] Kaistha B., Honstein T., Müller V., Bielak S., Sauer M., Kreider R., Fassan M., Scarpa A., Schmees C., Volkmer H. (2014). Key role of dual specificity kinase TTK in proliferation and survival of pancreatic cancer cells. Br. J. Cancer.

[46] Tsai Y.-M., Wu K.-L., Chang Y.-Y., Hung J.-Y., Chang W.-A., Chang C.-Y., Jian S.-F., Tsai P.-H., Huang Y.-C., Chong I.-W. (2020). Upregulation of Thr/Tyr kinase increases the cancer progression by neurotensin and dihydropyrimidinase-like 3 in lung cancer. Int. J. Mol. Sci..

[47] Basanta C.D.L.A.C., Bazzi M., Hijazi M., Bessant C., Cutillas P.R. (2023). Community detection in empirical kinase networks identifies new potential members of signalling pathways. PLoS Comput. Biol..

[48] Chen X., Yu C., Gao J., Zhu H., Cui B., Zhang T., Zhou Y., Liu Q., He H., Xiao R. (2018). A novel USP9X substrate TTK contributes to tumorigenesis in non-small-cell lung cancer. Theranostics.

[49] Miao R., Wu Y., Zhang H., Zhou H., Sun X., Csizmadia E., He L., Zhao Y., Jiang C., Miksad R.A. (2016). Utility of the dual-specificity protein kinase TTK as a therapeutic target for intrahepatic spread of liver cancer. Sci. Rep..

[50] Chen S., Wang J., Wang L., Peng H., Xiao L., Li C., Lin D., Yang K. (2019). Silencing TTK expression inhibits the proliferation and progression of prostate cancer. Exp. Cell Res..

[51] Jeffery J., Sinha D., Srihari S., Kalimutho M., Khanna K. (2016). Beyond cytokinesis: The emerging roles of CEP55 in tumorigenesis. Oncogene.

[52] Wang G., Chen B., Su Y., Qu N., Zhou D., Zhou W. (2023). CEP55 as a Promising Immune Intervention Marker to Regulate Tumor Progression: A Pan-Cancer Analysis with Experimental Verification. Cells.

[53] Lin K., Zhu X., Luo C., Bu F., Zhu J., Zhu Z. (2021). Data mining combined with experiments to validate CEP55 as a prognostic biomarker in colorectal cancer. Immun. Inflamm. Dis..

[54] Zaki M.S.A., Eldeen M.A., Abdulsahib W.K., Shati A.A., Alqahtani Y.A., Al-Qahtani S.M., Otifi H.M., Asiri A., Hassan H.M., Emam Mohammed Ahmed H. (2023). A comprehensive pan-cancer analysis identifies CEP55 as a potential oncogene and novel therapeutic target. Diagnostics.

[55] Li G.-S., Zhang W., Huang W.-Y., He R.-Q., Huang Z.-G., Gan X.-Y., Yang Z., Dang Y.-W., Kong J.-L., Zhou H.-F. (2023). CEP55: An immune-related predictive and prognostic molecular biomarker for multiple cancers. BMC Pulm. Med..

[56] Xie X., Liang H., Jiangting W., Wang Y., Ma X., Tan Z., Cheng L., Luo Z., Wang T. (2023). Cancer-testis antigen CEP55 serves as a prognostic biomarker and is correlated with immune infiltration and immunotherapy efficacy in pan-cancer. Front. Mol. Biosci..

[57] Kumar S., Sharma A.R., Sharma G., Chakraborty C., Kim J. (2016). PLK-1: Angel or devil for cell cycle progression. Biochim. Biophys. Acta (BBA)-Rev. Cancer.

[58] Milletti G., Colicchia V., Cecconi F. (2023). Cycl ers’ kinases in cell division: From molecules to cancer therapy. Cell Death Differ..

[59] Frosk P., Arts H.H., Philippe J., Gunn C.S., Brown E.L., Chodirker B., Simard L., Majewski J., Fahiminiya S., Russell C. (2017). A truncating mutation in CEP55 is the likely cause of MARCH, a novel syndrome affecting neuronal mitosis. J. Med. Genet..

[60] Bondeson M.L., Ericson K., Gudmundsson S., Ameur A., Pontén F., Wesström J., Frykholm C., Wilbe M. (2017). A nonsense mutation in CEP55 defines a new locus for a Meckel-like syndrome, an autosomal recessive lethal fetal ciliopathy. Clin. Genet..

[61] Sinha D., Nag P., Nanayakkara D., Duijf P.H., Burgess A., Raninga P., Smits V.A., Bain A.L., Subramanian G., Wall M. (2020). Cep55 overexpression promotes genomic instability and tumorigenesis in mice. Commun. Biol..

[62] Kalimutho M., Sinha D., Jeffery J., Nones K., Srihari S., Fernando W.C., Duijf P.H., Vennin C., Raninga P., Nanayakkara D. (2018). CEP 55 is a determinant of cell fate during perturbed mitosis in breast cancer. EMBO Mol. Med..

[63] Jiang C., Zhang Y., Li Y., Lu J., Huang Q., Xu R., Feng Y., Yan S. (2018). High CEP55 expression is associated with poor prognosis in non-small-cell lung cancer. OncoTargets Ther..

[64] Xu L., Xia C., Sheng F., Sun Q., Xiong J., Wang S. (2018). CEP55 promotes the proliferation and invasion of tumour cells via the AKT signalling pathway in osteosarcoma. Carcinogenesis.

[65] Chen C., Lu P., Chen Y., Fu S., Wu K., Tsou A., Lee Y., Lin T., Hsu S., Lin W. (2007). FLJ10540-elicited cell transformation is through the activation of PI3-kinase/AKT pathway. Oncogene.

[66] Yang C., Yang Y., Wang W., Zhou W., Zhang X., Xiao Y., Zhang H. (2022). CEP55 3’-UTR promotes epithelial–mesenchymal transition and enhances tumorigenicity of bladder cancer cells by acting as a ceRNA regulating miR-497-5p. Cell. Oncol..

[67] Zhang X., Xu Q., Li E., Shi T., Chen H. (2023). CEP55 predicts the poor prognosis and promotes tumorigenesis in endometrial cancer by regulating the Foxo1 signaling. Mol. Cell. Biochem..

[68] Yan S.-M., Liu L., Gu W.-Y., Huang L.-Y., Yang Y., Huang Y.-H., Luo R.-Z. (2021). CEP55 positively affects tumorigenesis of esophageal squamous cell carcinoma and is correlated with poor prognosis. J. Oncol..

[69] Wu S., Wu D., Pan Y., Liu H., Shao Z., Wang M. (2019). Correlation between EZH2 and CEP55 and lung adenocarcinoma prognosis. Pathol. -Res. Pract..

[70] Zhou X., Wang P., Zhao H. (2018). The association between AURKA gene rs2273535 polymorphism and gastric cancer risk in a Chinese population. Front. Physiol..

[71] Qi J., Gao X., Zhong X., Zhang N., Wang R., Zhang H., Pan T., Liu X., Yao Y., Wu Q. (2019). Selective inhibition of Aurora A and B kinases effectively induces cell cycle arrest in t (8; 21) acute myeloid leukemia. Biomed. Pharmacother..

[72] Hu X., Zhou Y., Hill C., Chen K., Cheng C., Liu X., Duan P., Gu Y., Wu Y., Ewing R.M. (2024). Identification of MYCN non-amplified neuroblastoma subgroups points towards molecular signatures for precision prognosis and therapy stratification. Br. J. Cancer.

[73] Goldenson B., Crispino J.D. (2015). The aurora kinases in cell cycle and leukemia. Oncogene.

[74] Lu H., Gomaa A., Wang-Bishop L., Ballout F., Hu T., McDonald O., Washington M.K., Livingstone A.S., Wang T.C., Peng D. (2022). Unfolded protein response is activated by aurora kinase A in esophageal adenocarcinoma. Cancers.

[75] Mendiola M., Barriuso J., Mariño-Enríquez A., Redondo A., Domínguez-Cáceres A., Hernández-Cortés G., Pérez-Fernández E., Sánchez-Navarro I., Vara J.Á.F., Suárez A. (2009). Aurora kinases as prognostic biomarkers in ovarian carcinoma. Hum. Pathol..

[76] Dong B., Chai M., Chen H., Feng Q., Jin R., Hu S. (2020). Screening and verifying key genes with poor prognosis in colon cancer through bioinformatics analysis. Transl. Cancer Res..

[77] Chuang T.-P., Wang J.-Y., Jao S.-W., Wu C.-C., Chen J.-H., Hsiao K.-H., Lin C.-Y., Chen S.-H., Su S.-Y., Chen Y.-J. (2016). Over-expression of AURKA, SKA3 and DSN1 contributes to colorectal adenoma to carcinoma progression. Oncotarget.

[78] Panagopoulos A., Taraviras S., Nishitani H., Lygerou Z. (2020). CRL4Cdt2: Coupling genome stability to ubiquitination. Trends Cell Biol..

[79] Kiran S., Dar A., Singh S.K., Lee K.Y., Dutta A. (2018). The deubiquitinase USP46 is essential for proliferation and tumor growth of HPV-transformed cancers. Mol. Cell.

[80] Luo Y., He Z., Liu W., Zhou F., Liu T., Wang G. (2022). DTL is a prognostic biomarker and promotes bladder cancer progression through regulating the AKT/mTOR axis. Oxidative Med. Cell. Longev..

[81] Li Z., Wang R., Qiu C., Cao C., Zhang J., Ge J., Shi Y. (2022). Role of DTL in hepatocellular carcinoma and its impact on the tumor microenvironment. Front. Immunol..

[82] Lu J.-J., Chen F.-J., Li Y., Xu X., Peng C., Yu N., Su L.-N., Tang L. (2022). DTL promotes melanoma progression through rewiring cell glucose metabolism. Ann. Transl. Med..

[83] Chang X., Jian L. (2022). LncRNA ZFPM2-AS1 drives the progression of nasopharyngeal carcinoma via modulating the downstream miR-3612/DTL signaling. Anti-Cancer Drugs.

[84] Cui H., Wang Q., Lei Z., Feng M., Zhao Z., Wang Y., Wei G. (2019). DTL promotes cancer progression by PDCD4 ubiquitin-dependent degradation. J. Exp. Clin. Cancer Res..

[85] Tang Y., Lei Y., Gao P., Jia J., Du H., Wang Q., Yan Z., Zhang C., Liang G., Wang Y. (2023). Pan-cancer analysis and experimental validation of DTL as a potential diagnosis, prognosis and immunotherapy biomarker. BMC Cancer.

[86] Wu C., Tan J., Wang X., Qin C., Long W., Pan Y., Li Y., Liu Q. (2023). Pan-cancer analyses reveal molecular and clinical characteristics of cuproptosis regulators. Imeta.

