# Supplementary material for: Identification of Novel Molecular Panel as Potential Biomarkers of PAN-Gastrointestinal Cancer Screening: Bioinformatics and Experimental Analysis
Source: Biology (Basel). 2025 Jul 2;14(7):803. doi: 10.3390/biology14070803 (PMC12292149; doi:10.3390/biology14070803)

Suplementary Figure S1.

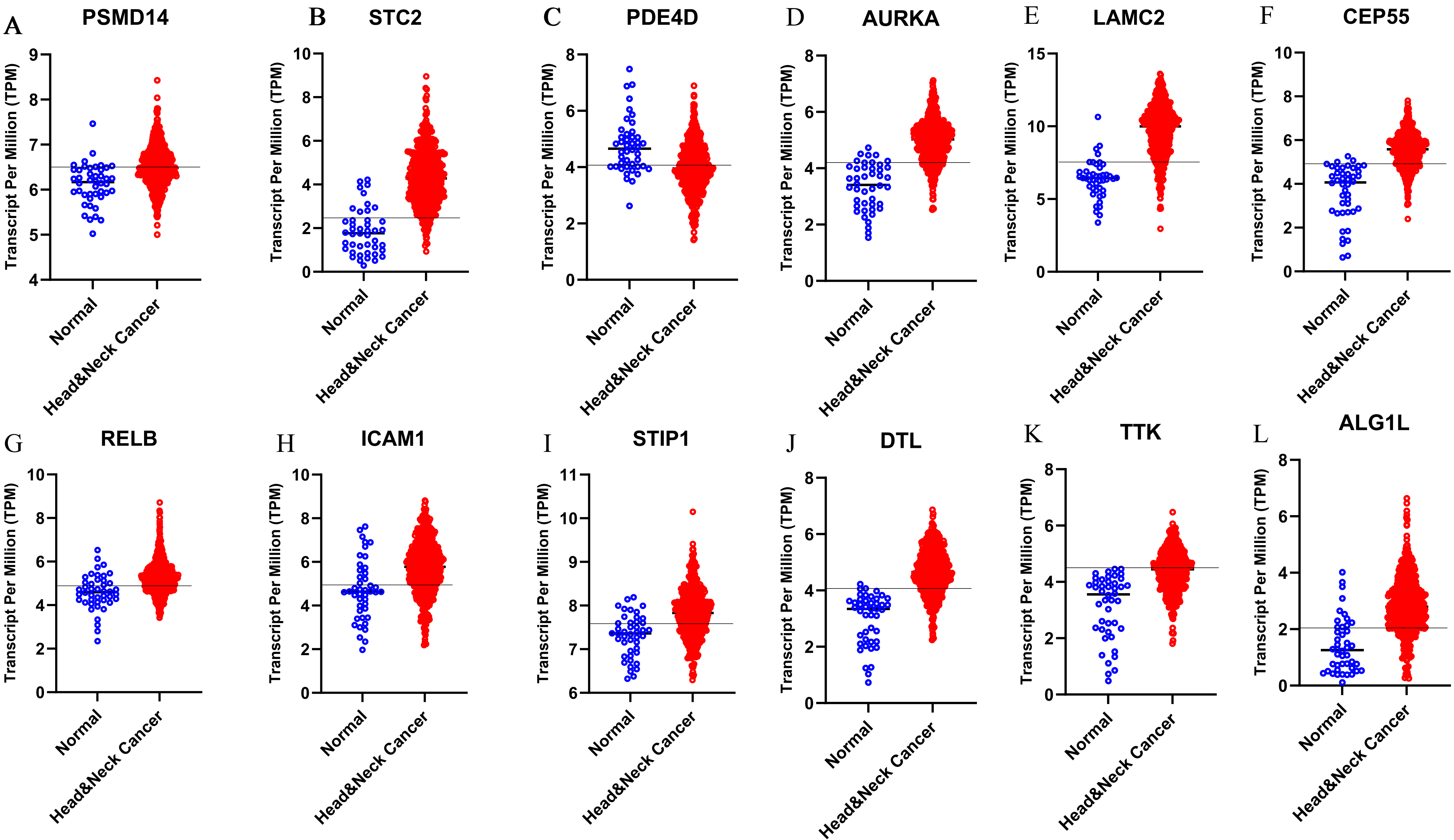

Suplementary Figure S1.

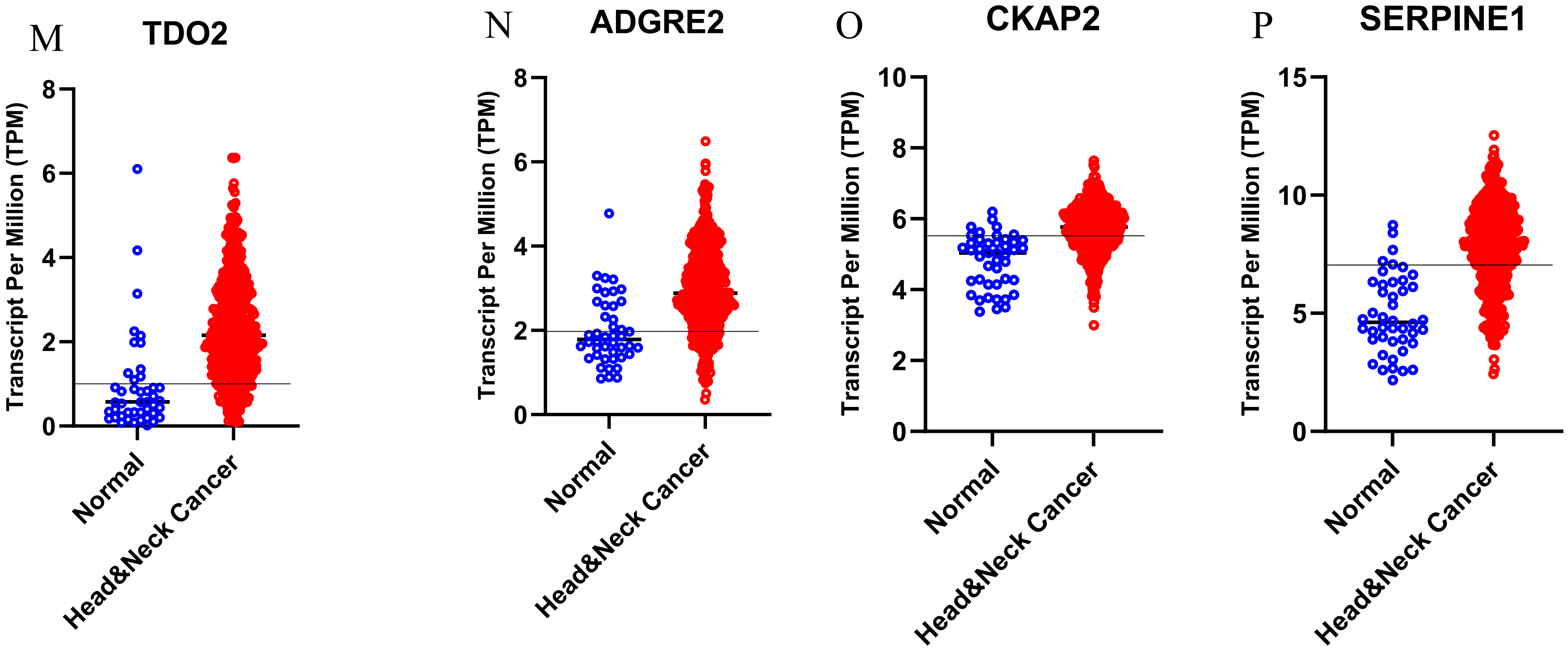

Suplementary Figure S2.

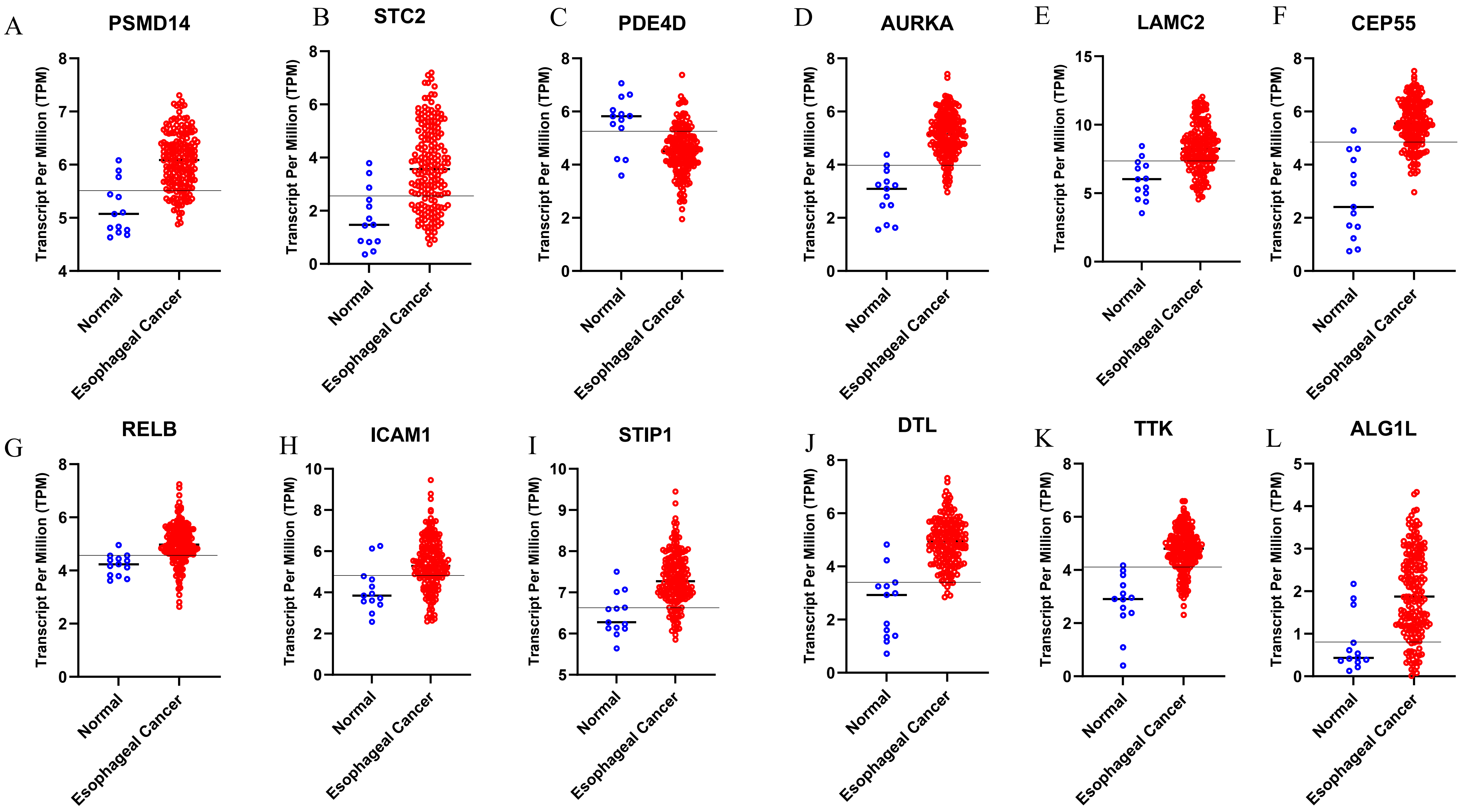

Suplementary Figure S2.

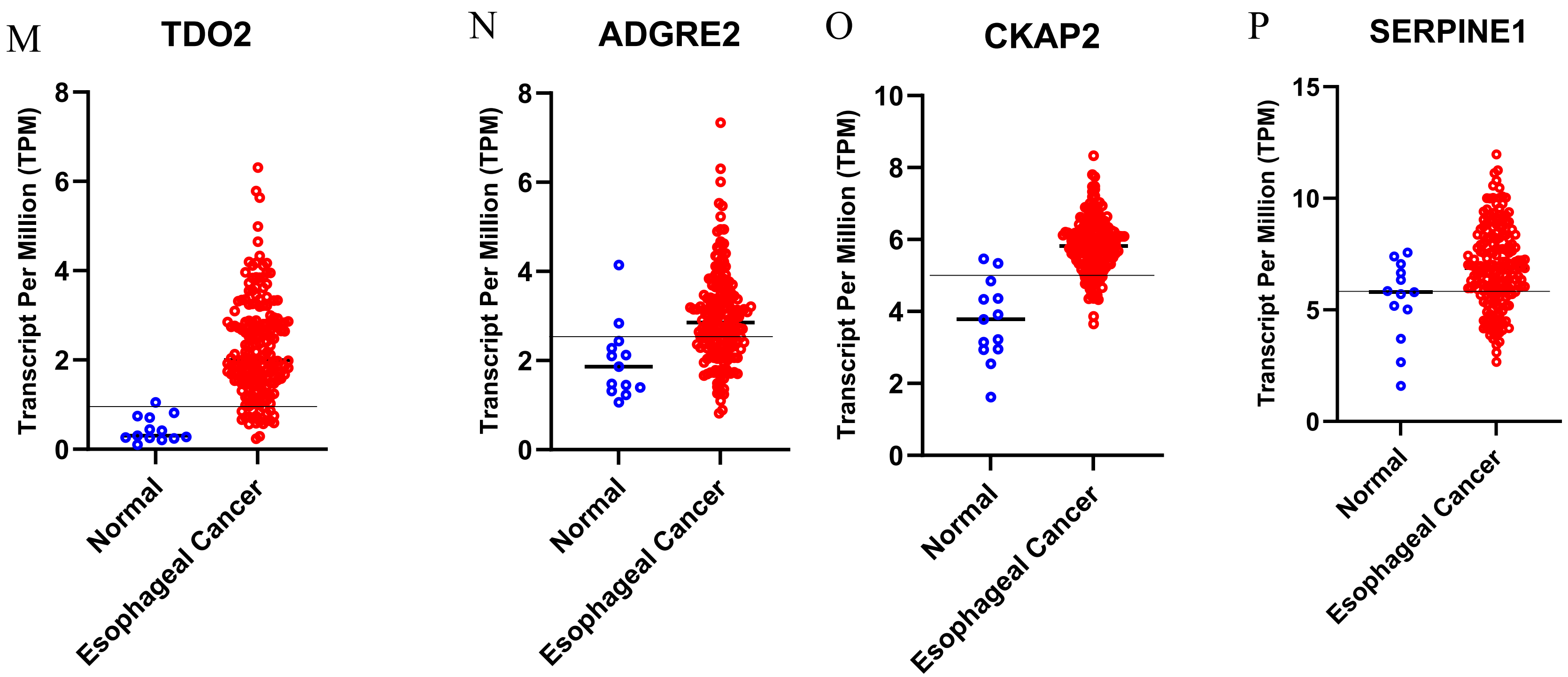

Suplementary Figure S3.

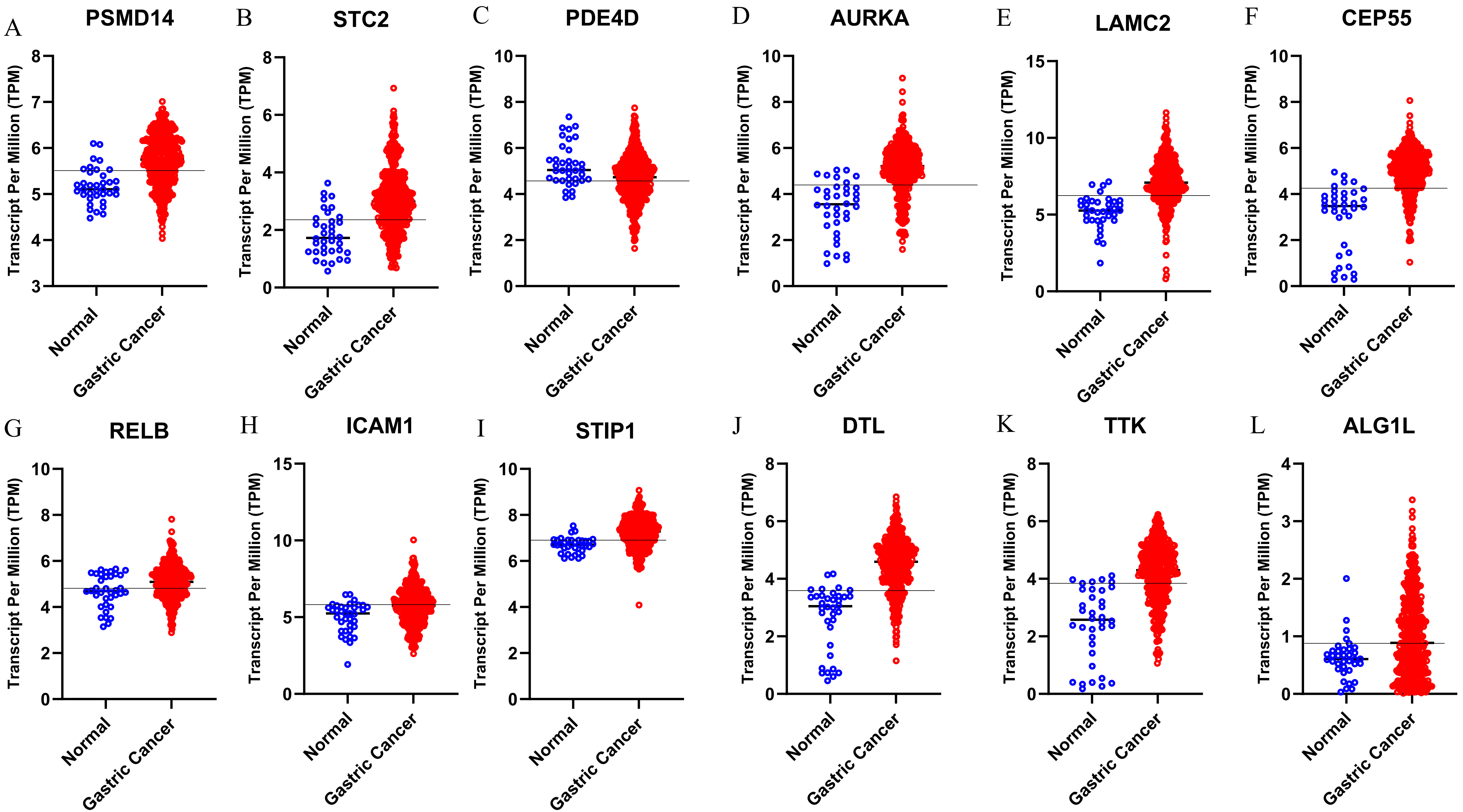

Suplementary Figure S3.

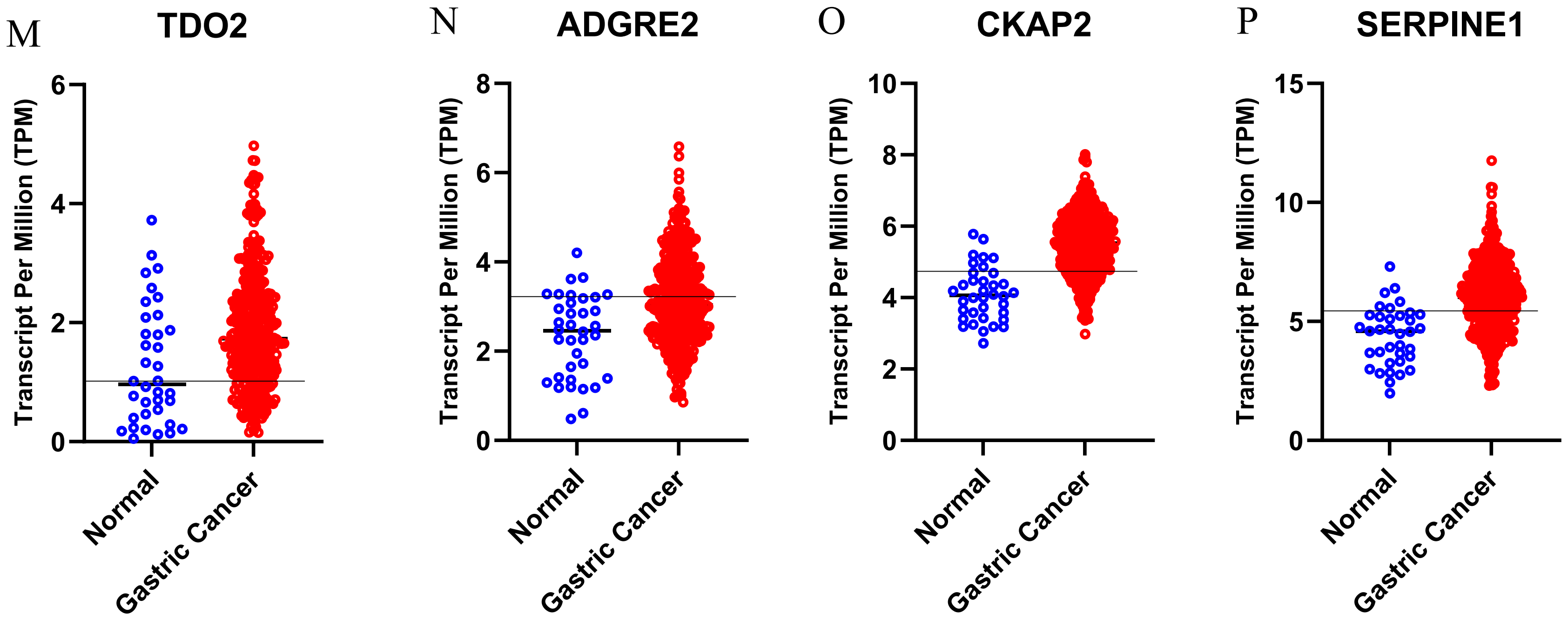

Suplementary Figure S4.

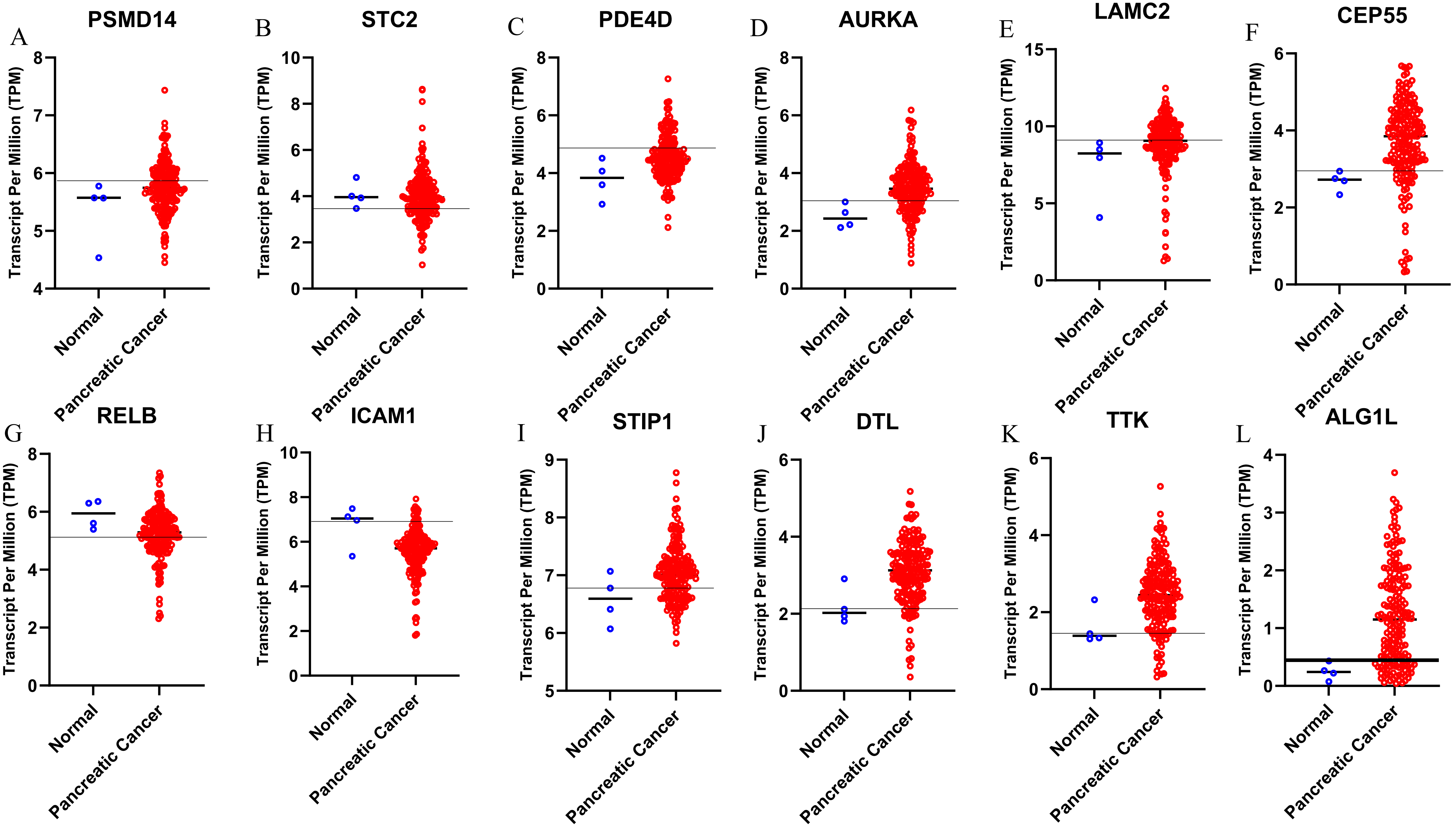

Suplementary Figure S4.

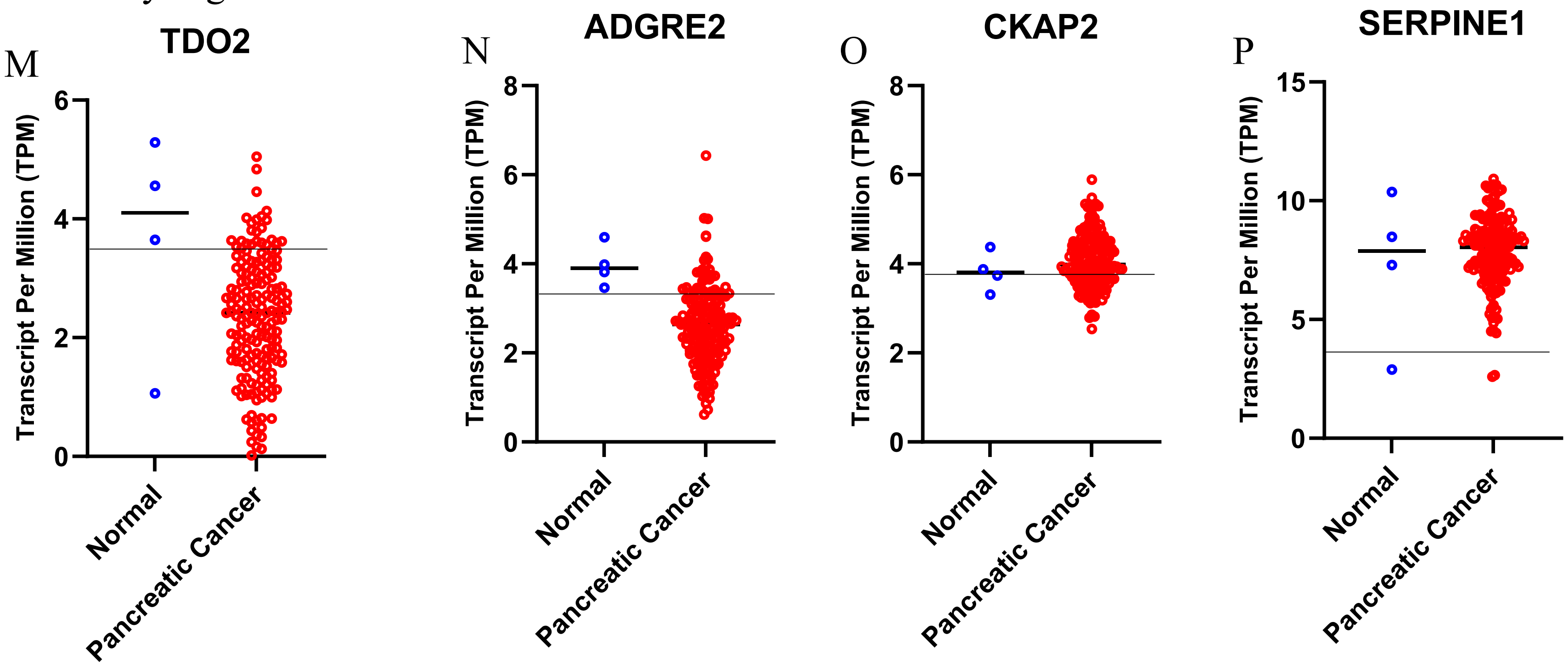

Suplementary Figure S5.

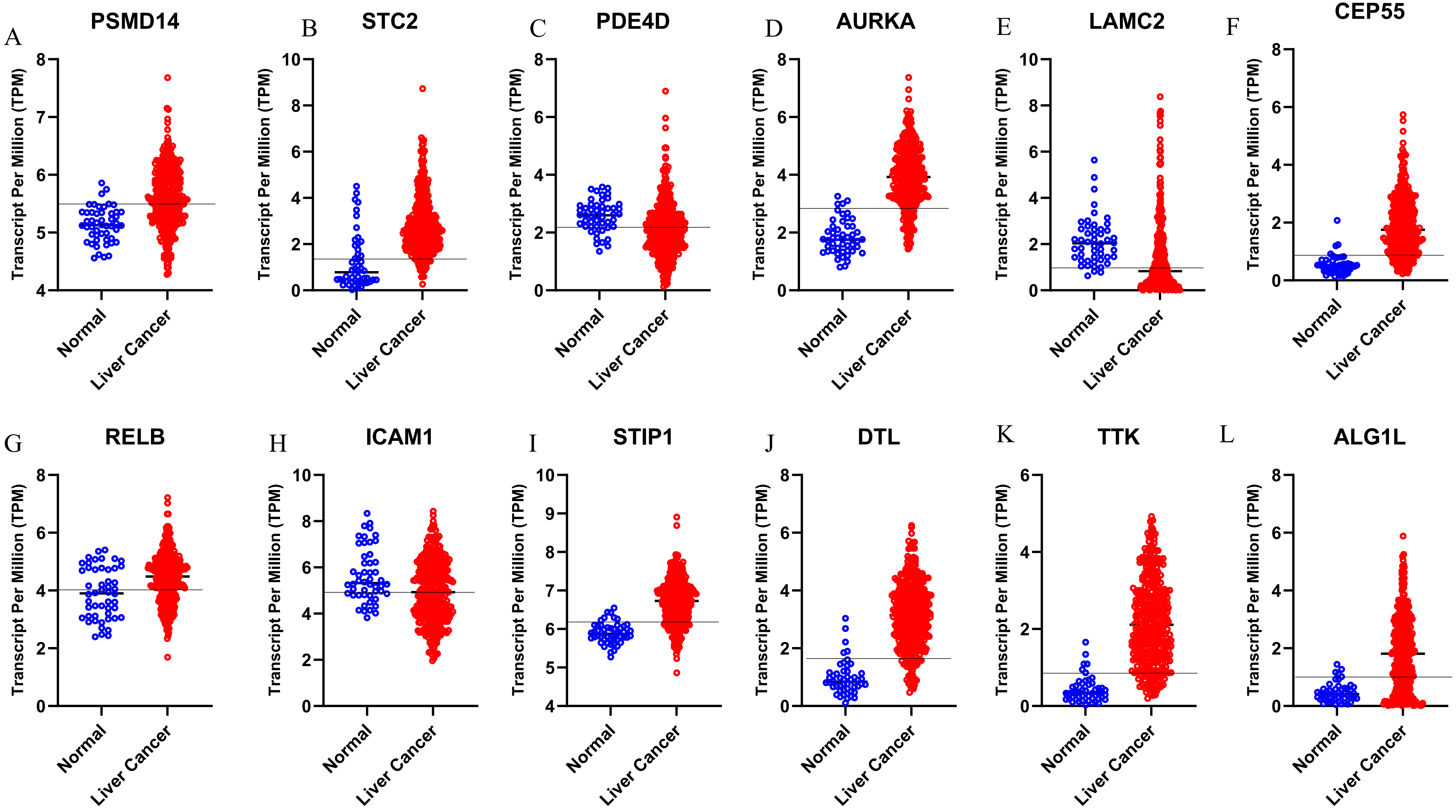

Suplementary Figure S5.

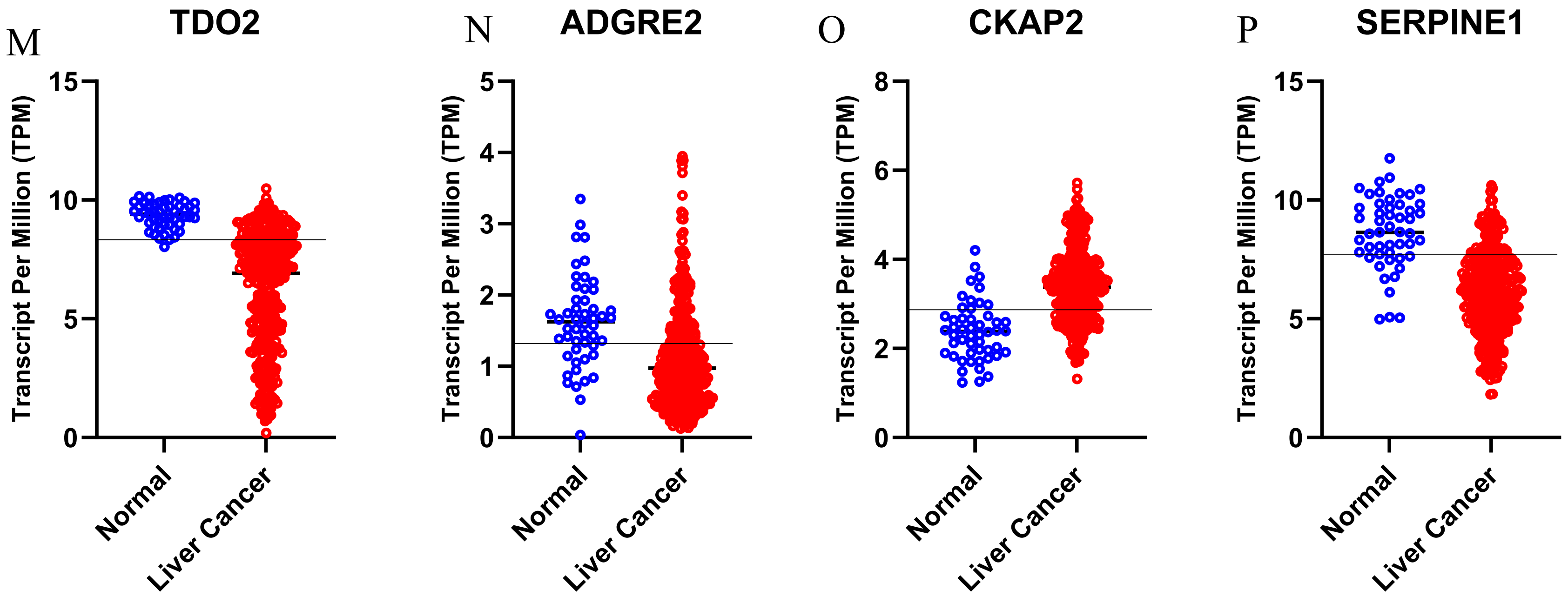

Suplementary Figure S6.

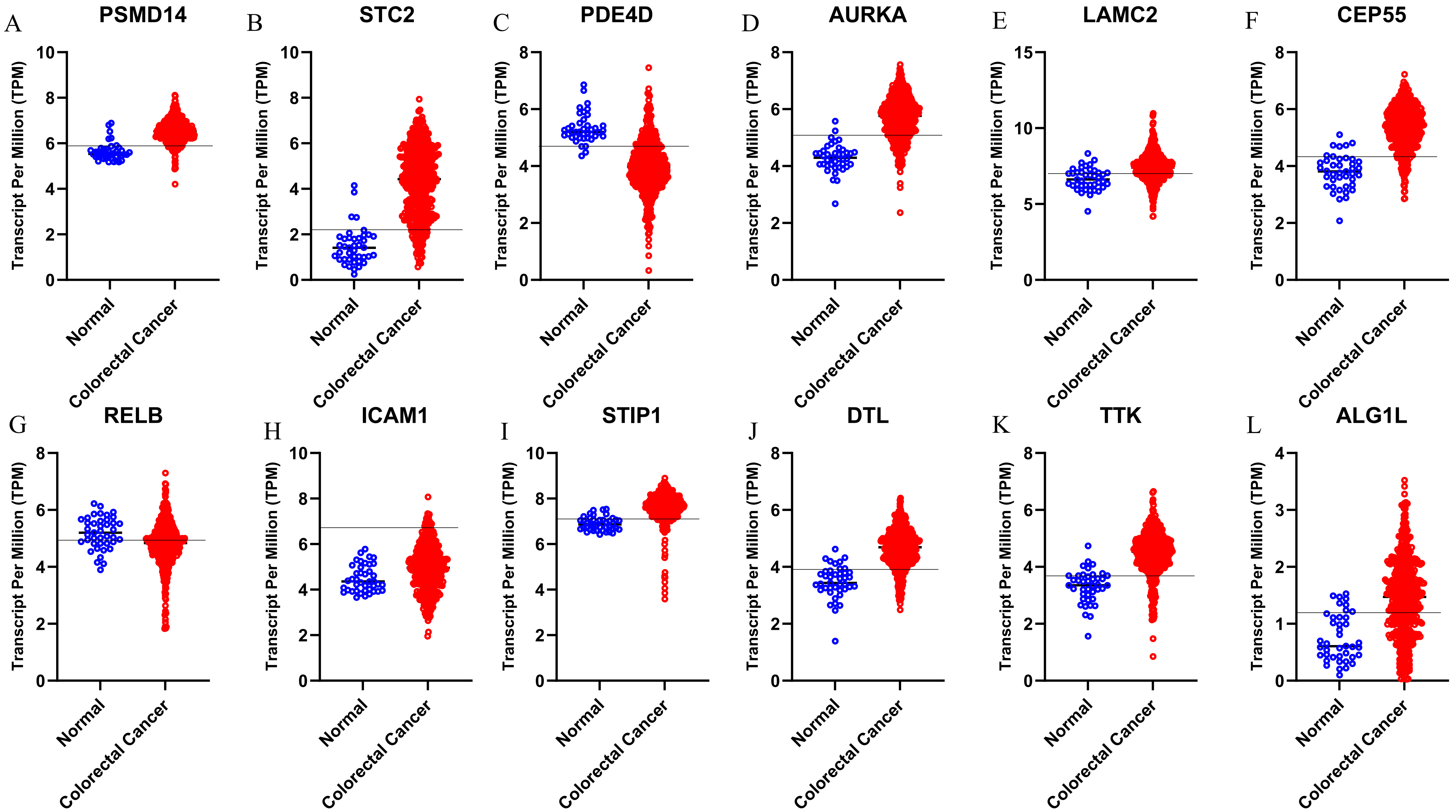

Suplementary Figure S6.

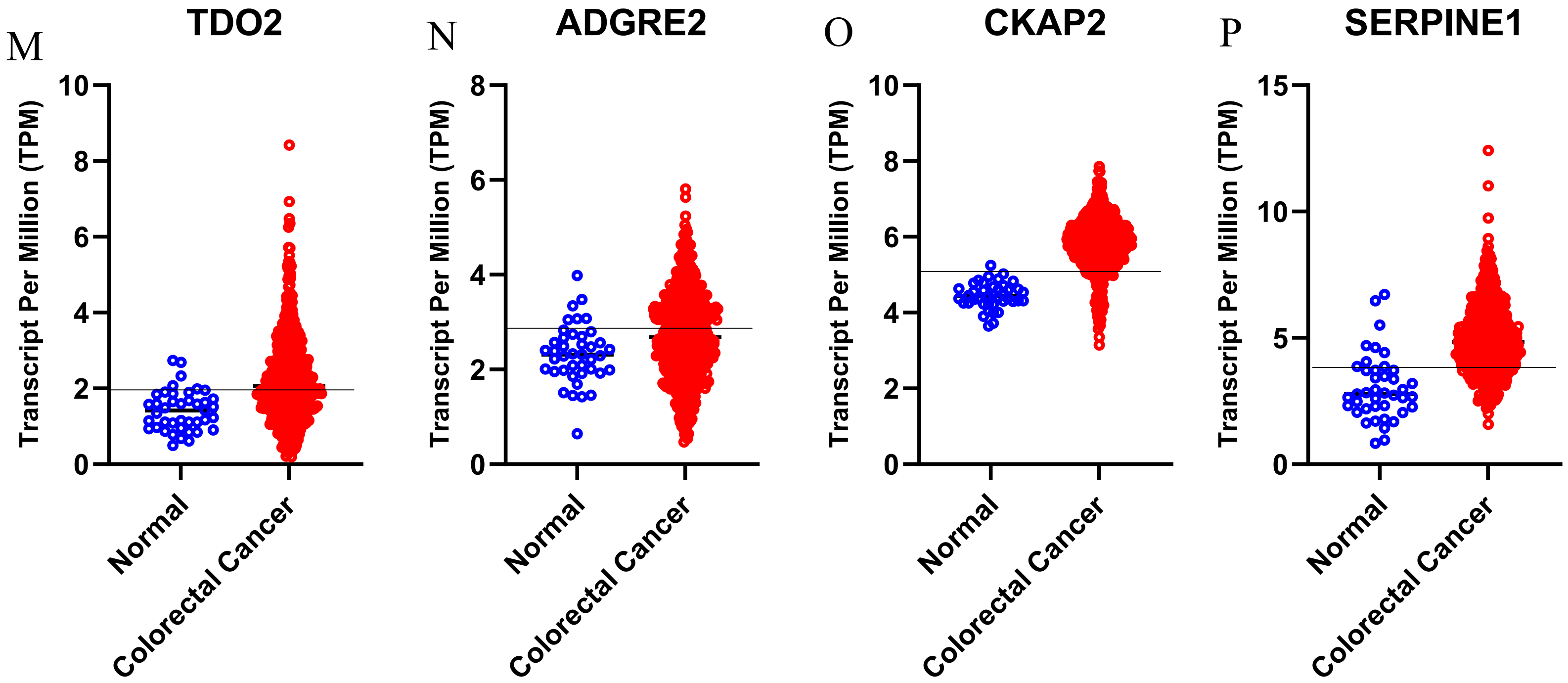

Suplementary Figure S7.

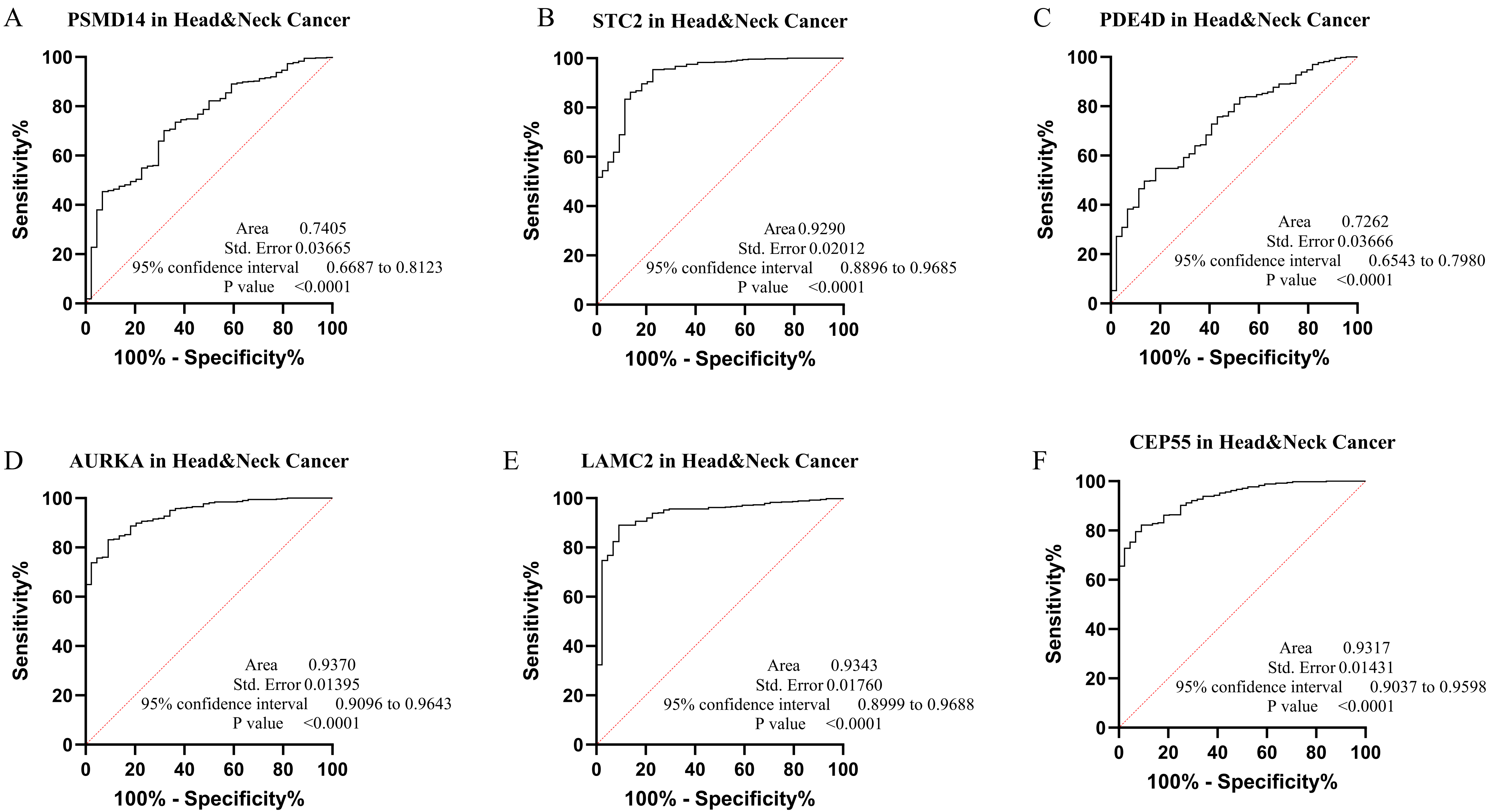

Suplementary Figure S7.

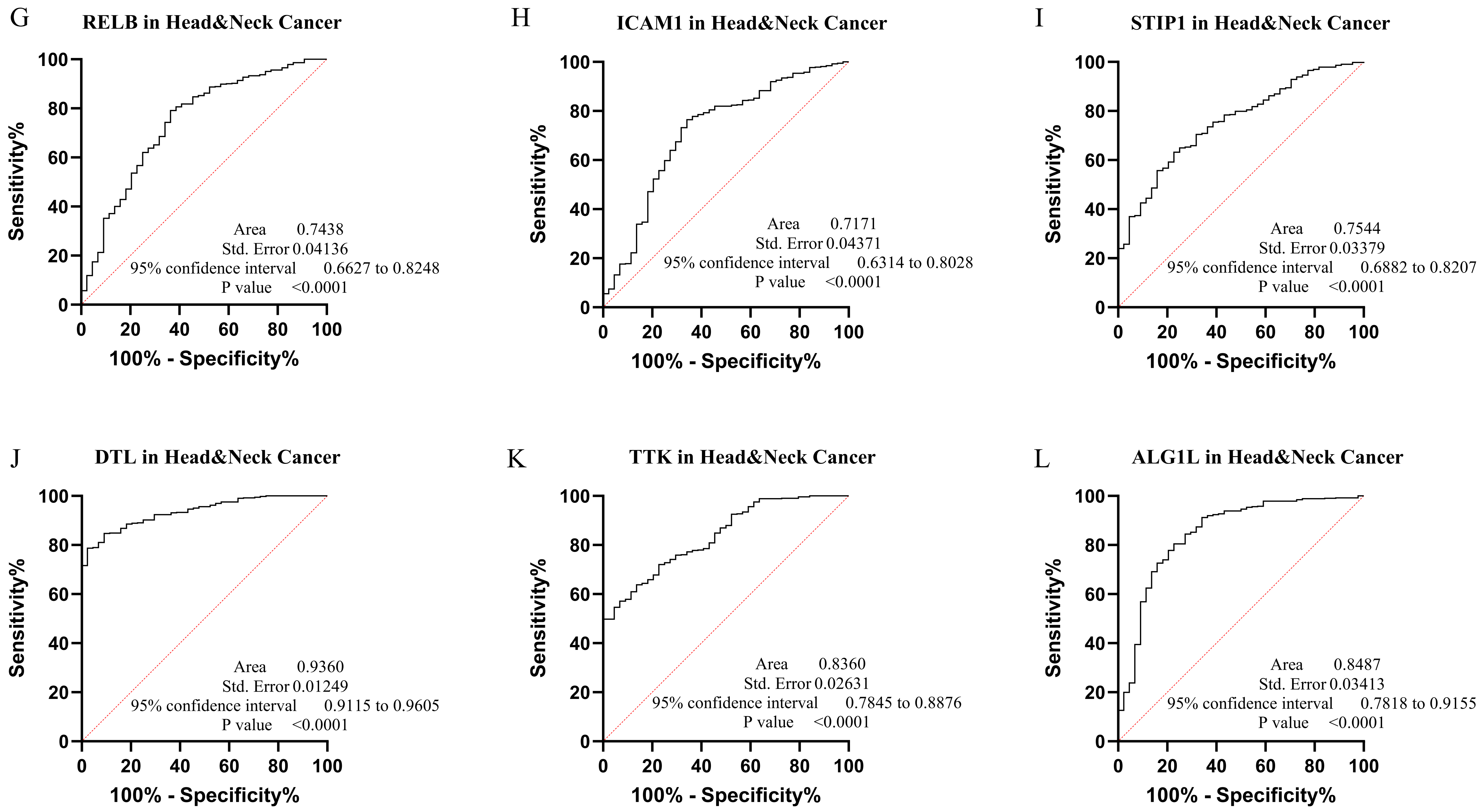

Suplementary Figure S7.

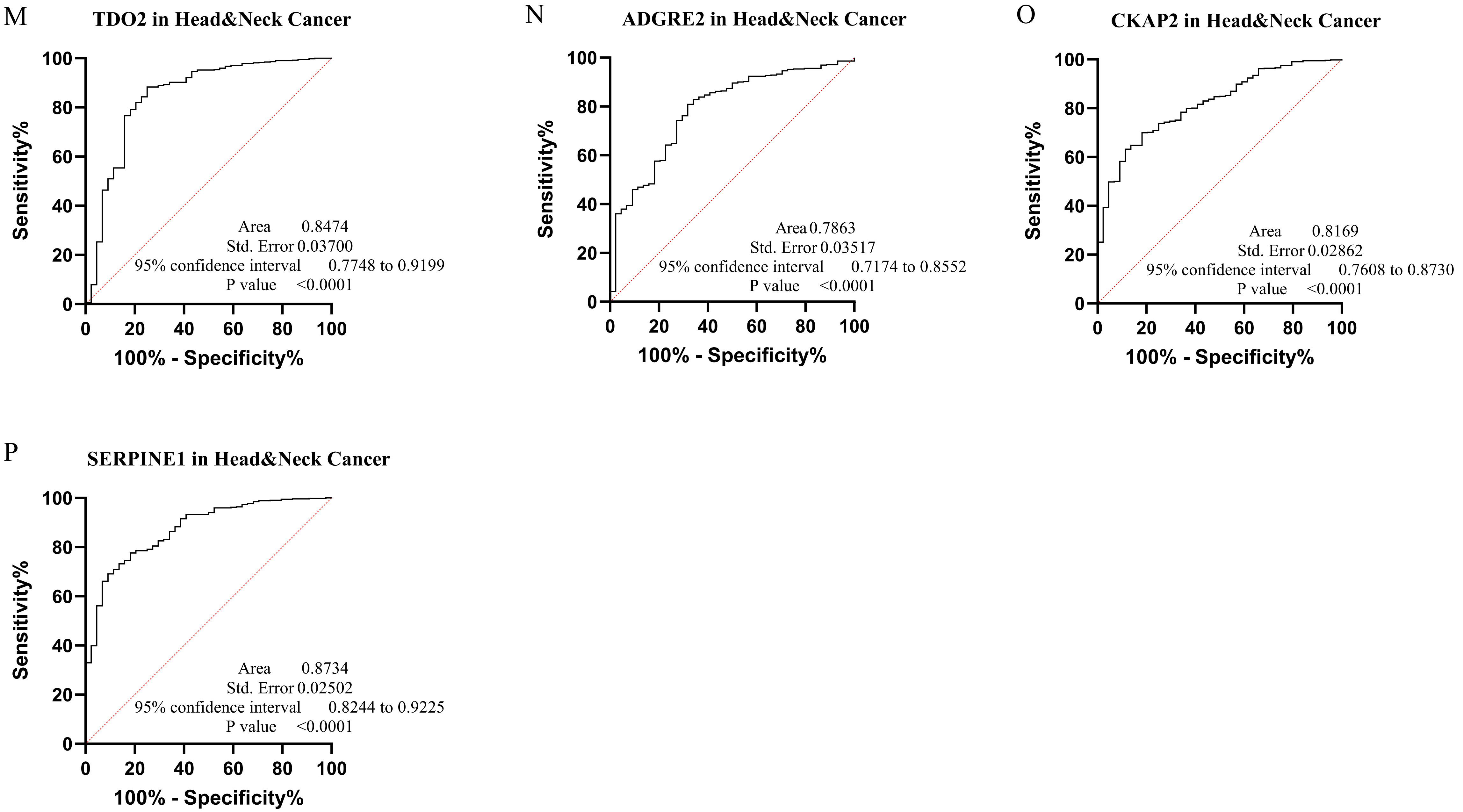

Suplementary Figure S8.

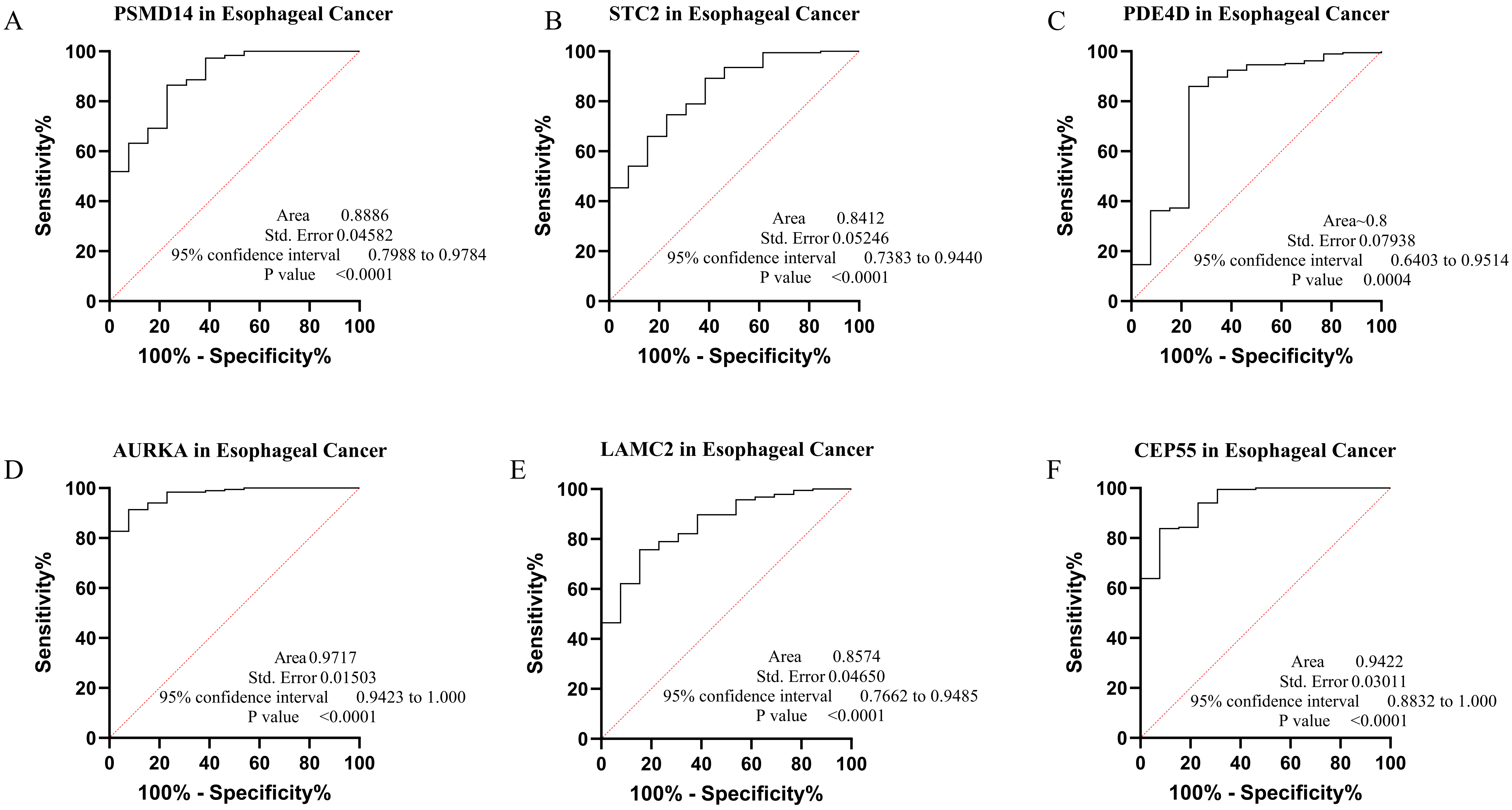

Suplementary Figure S8.

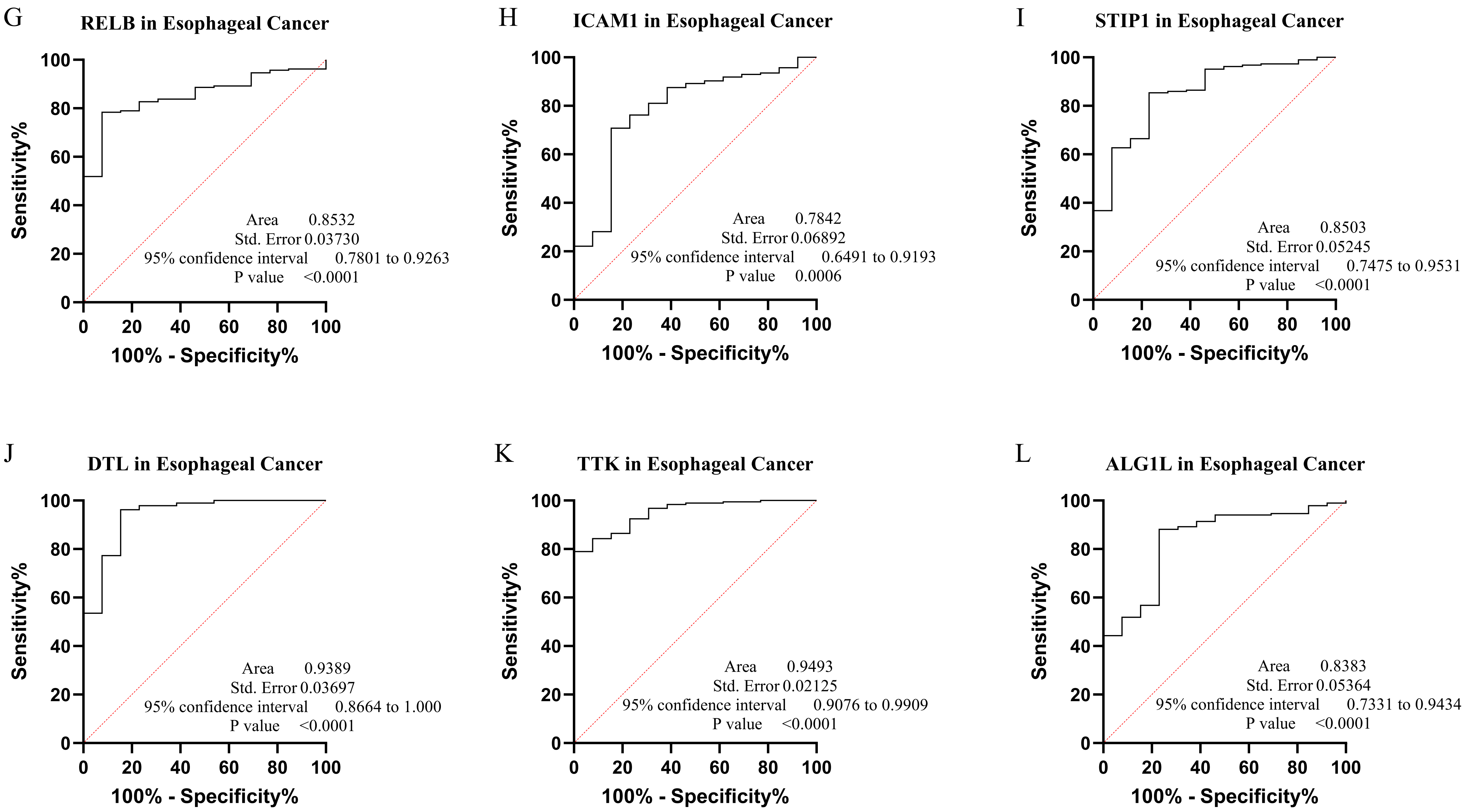

Suplementary Figure S8.

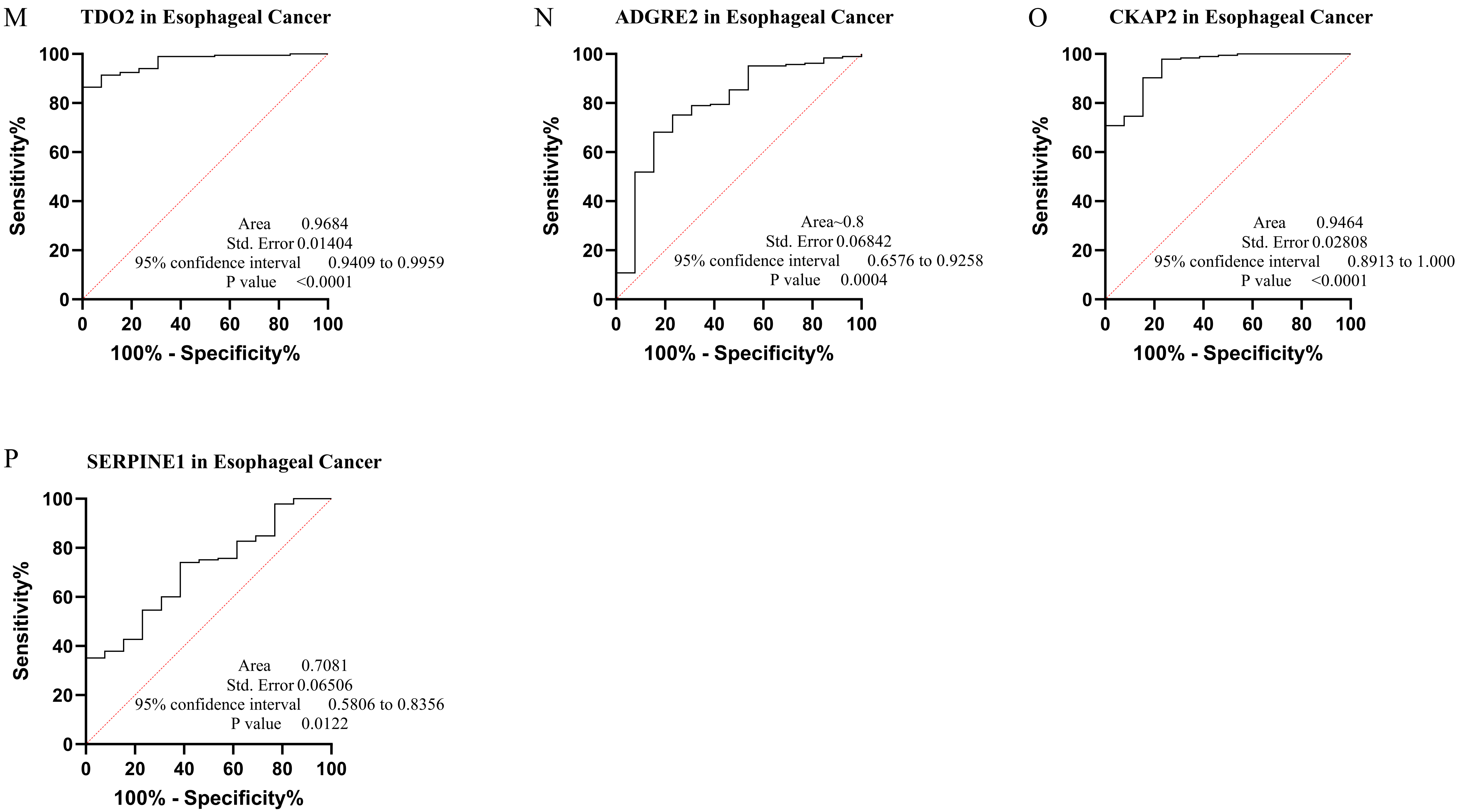

Suplementary Figure S9.

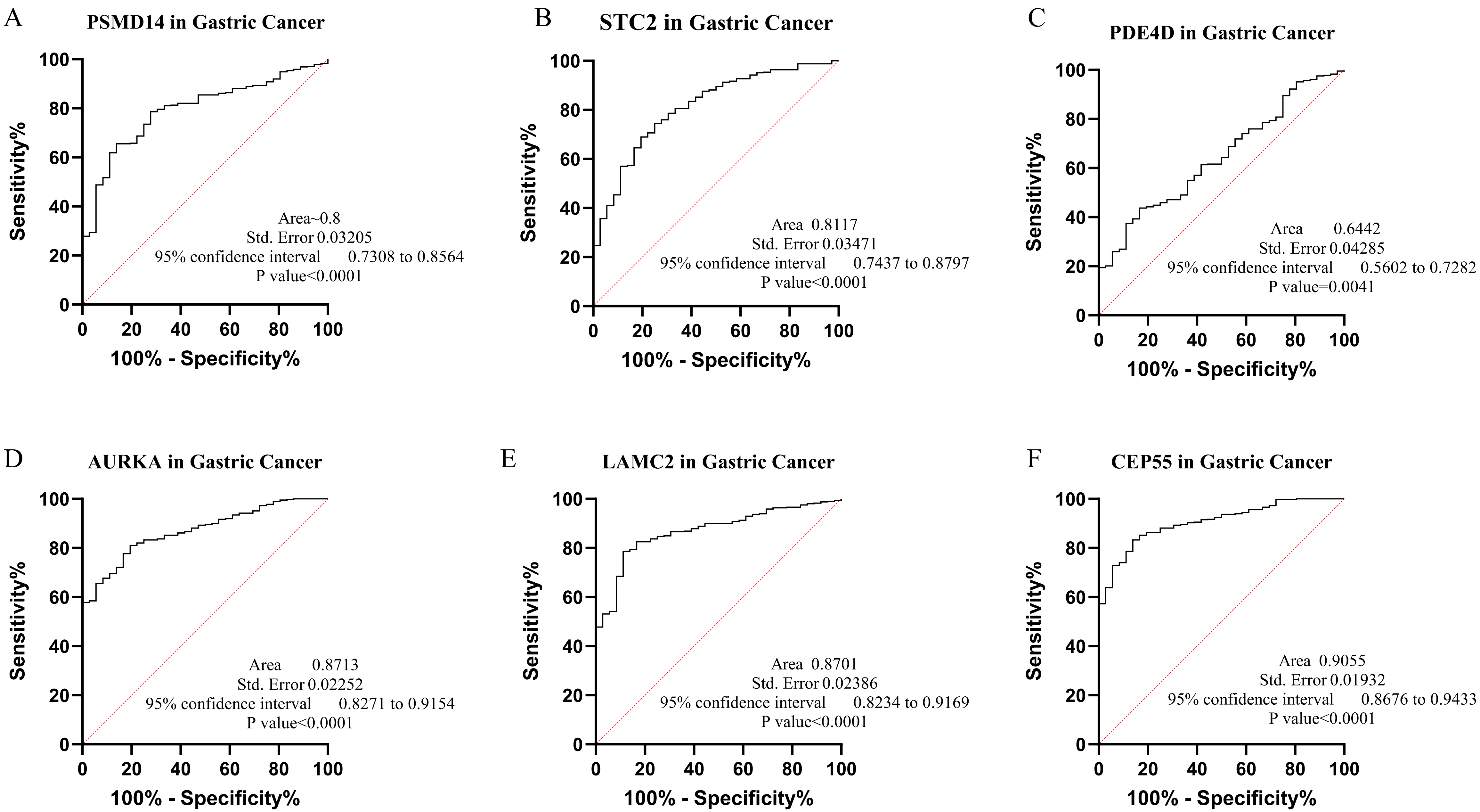

Suplementary Figure S9.

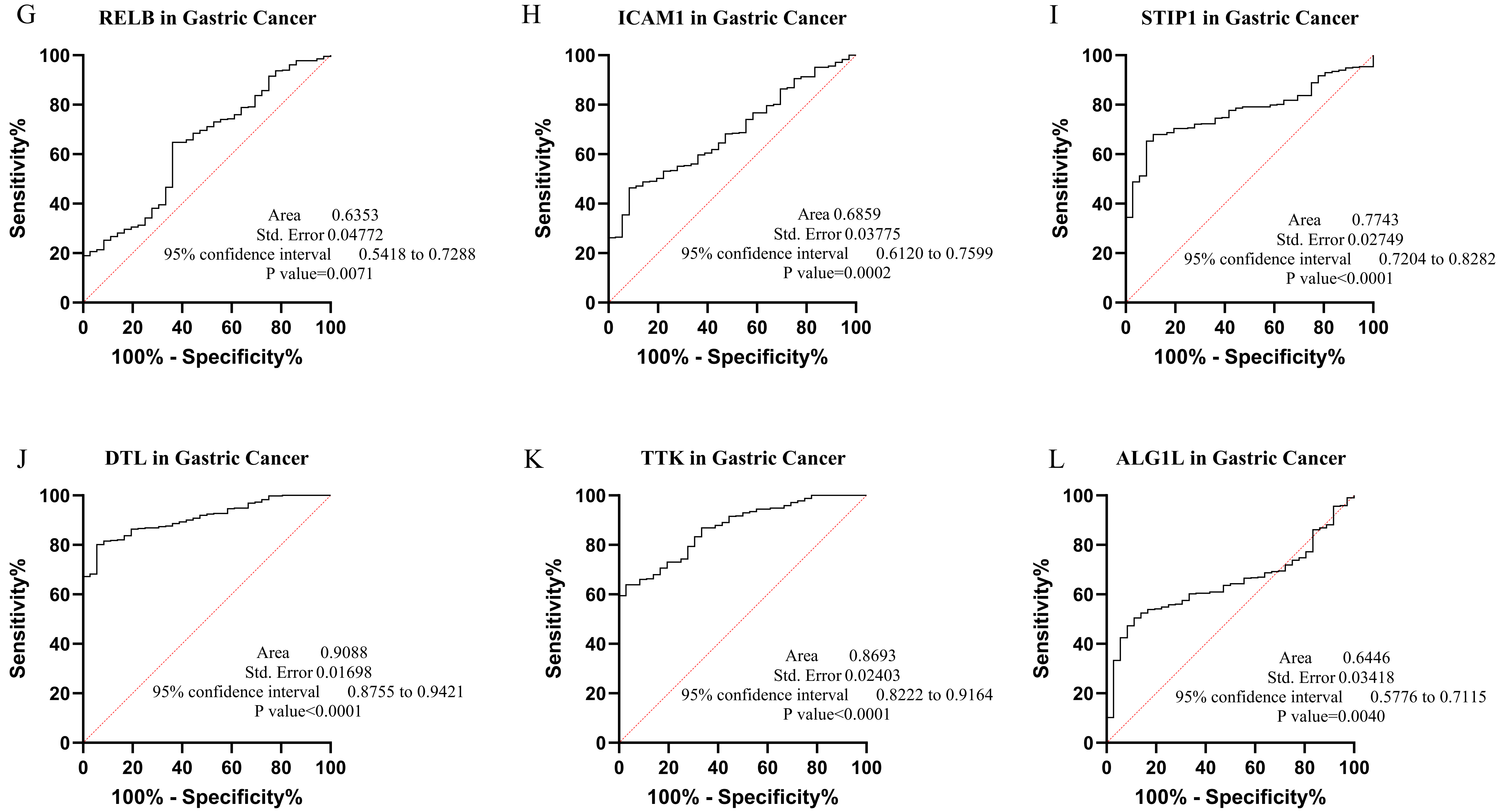

Suplementary Figure S9.

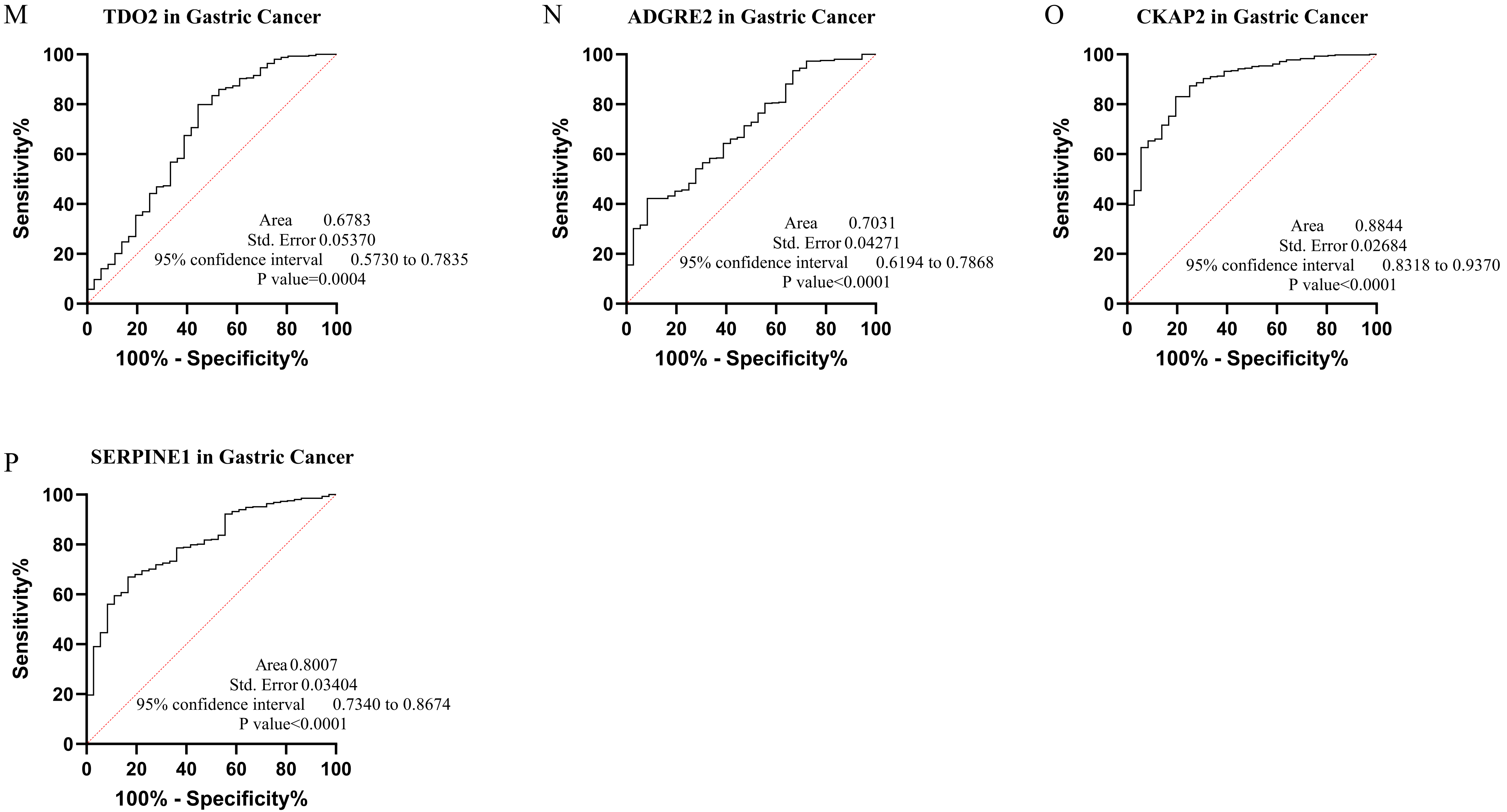

Suplementary Figure S10.

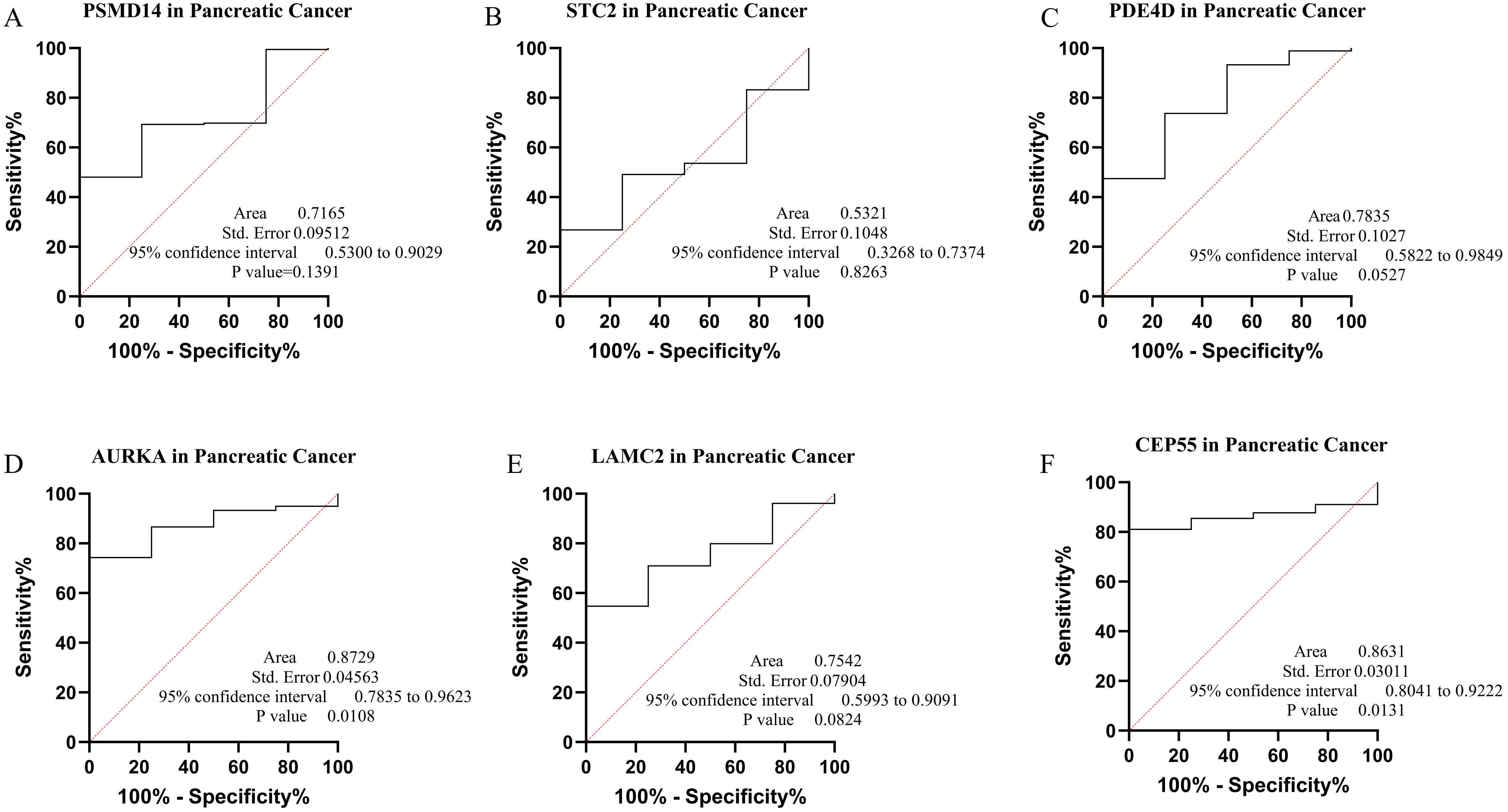

Suplementary Figure S10.

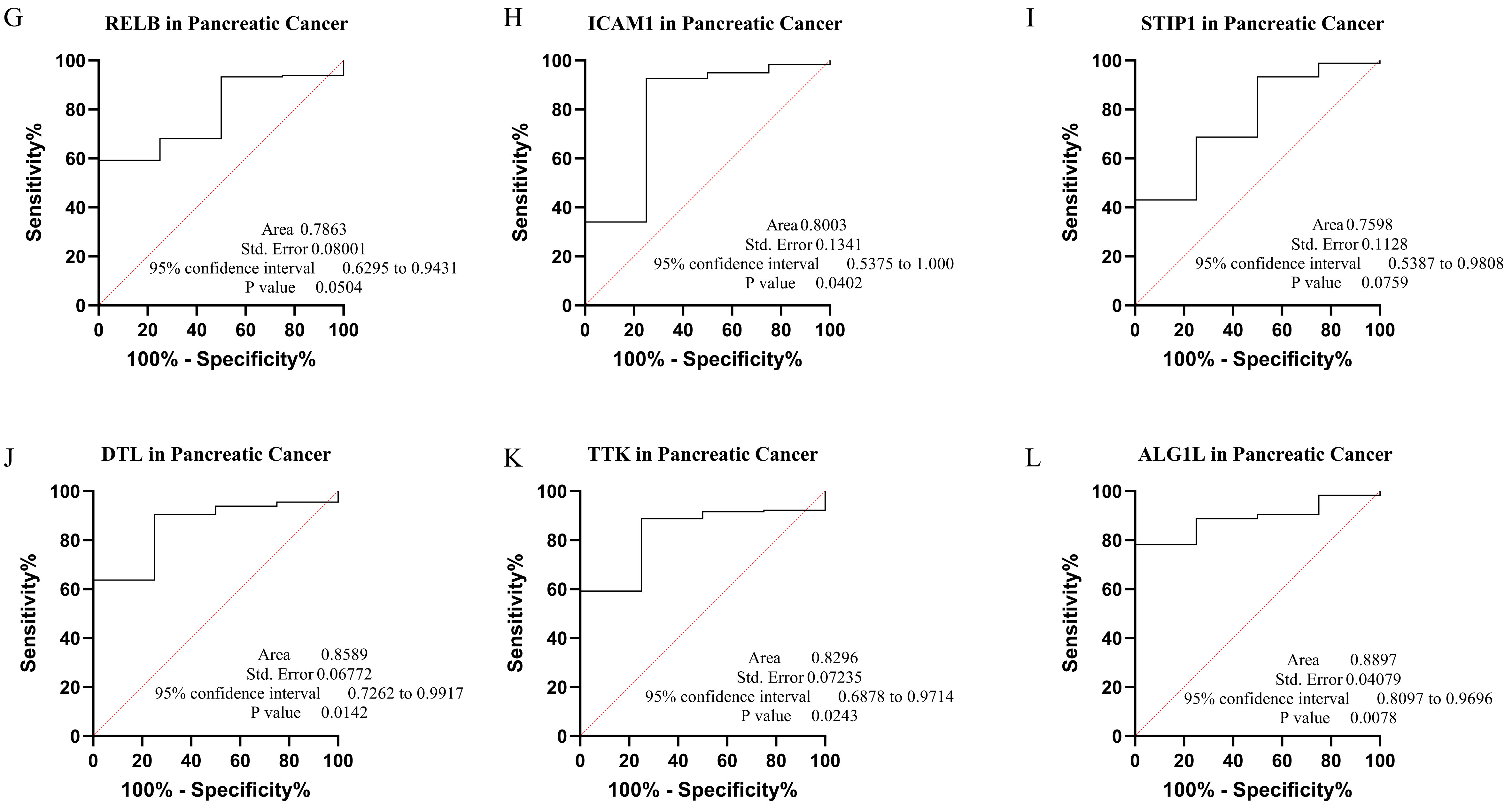

Suplementary Figure S10.

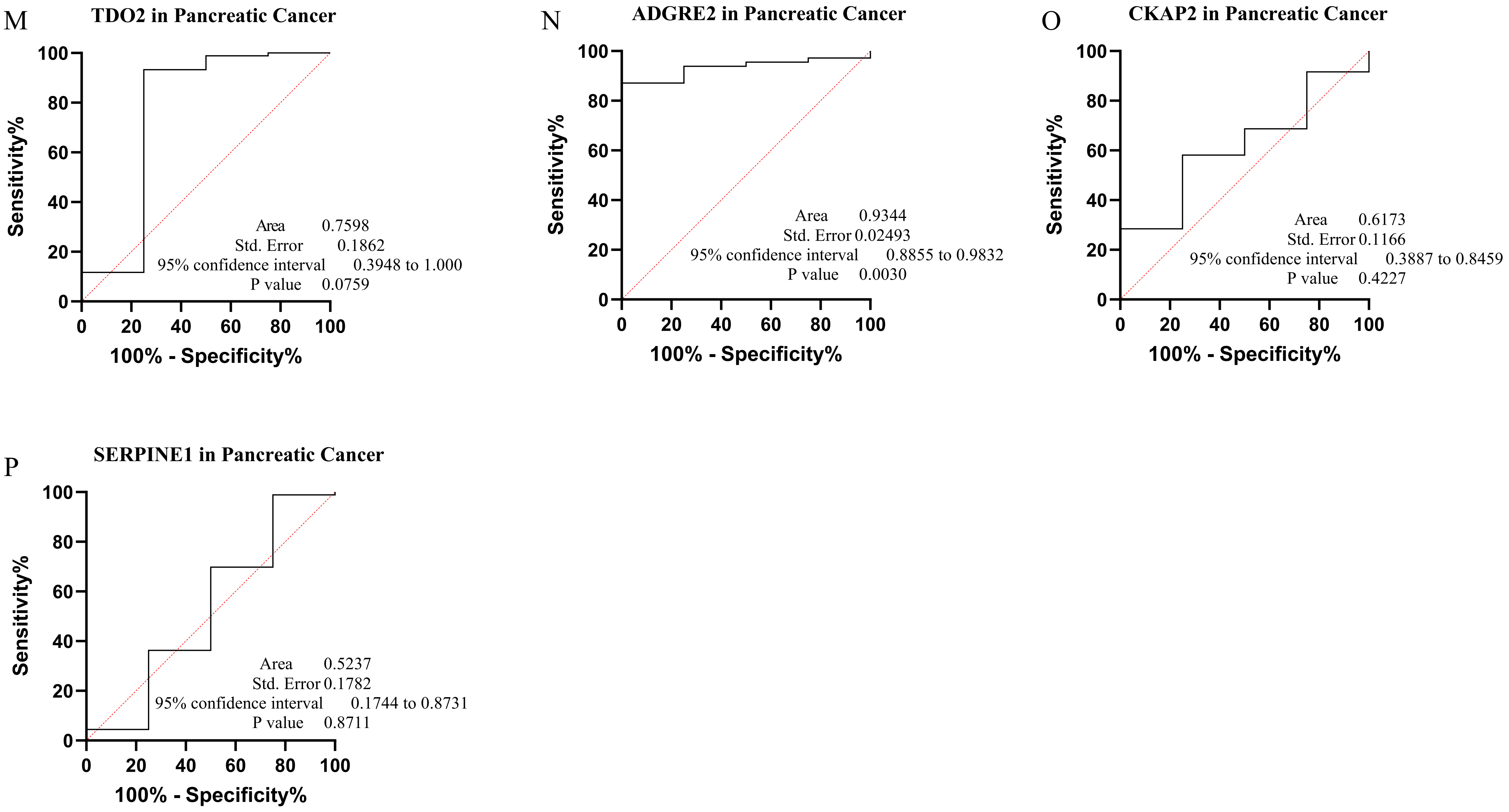

Suplementary Figure S11.

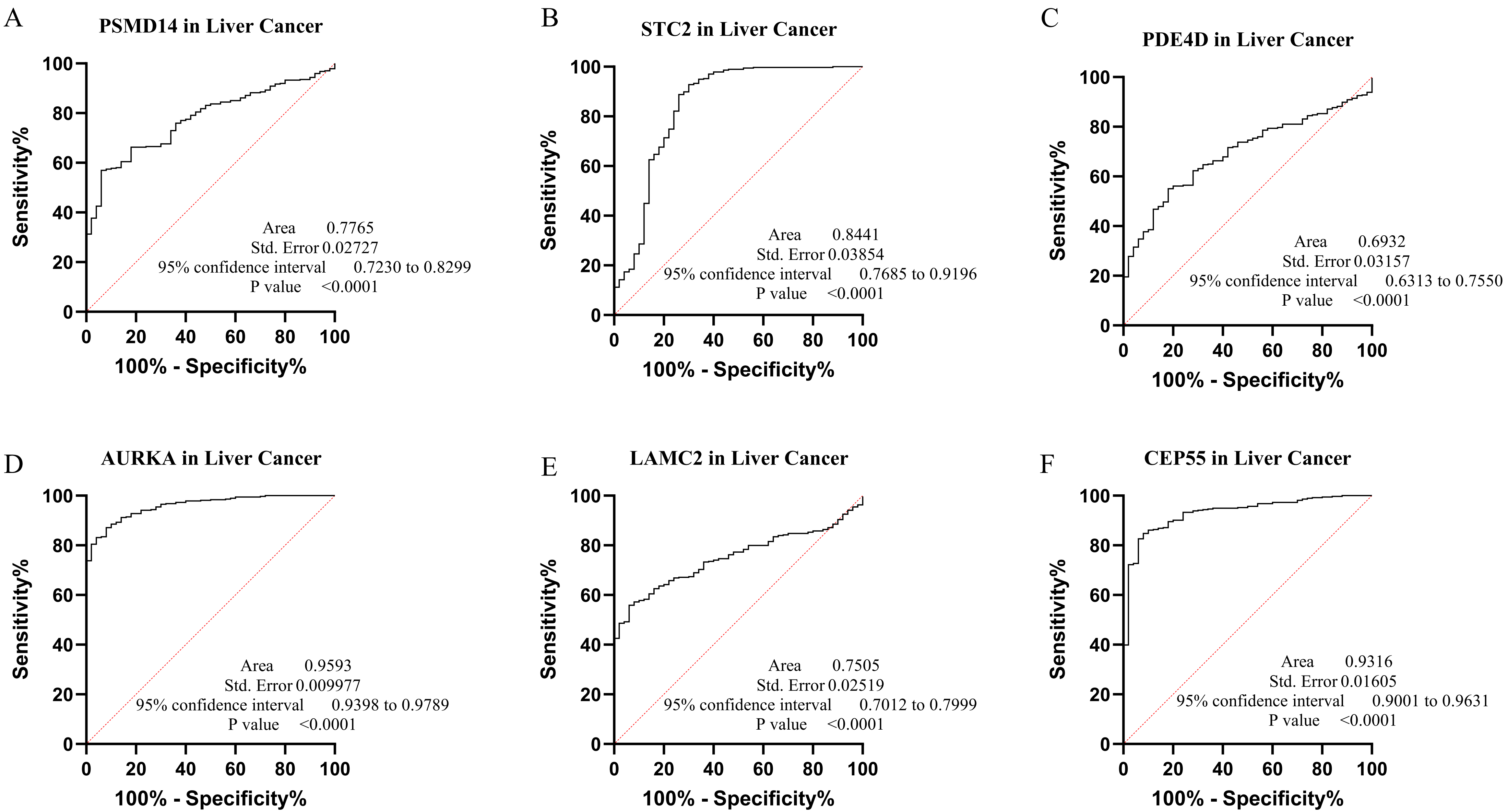

Suplementary Figure S11.

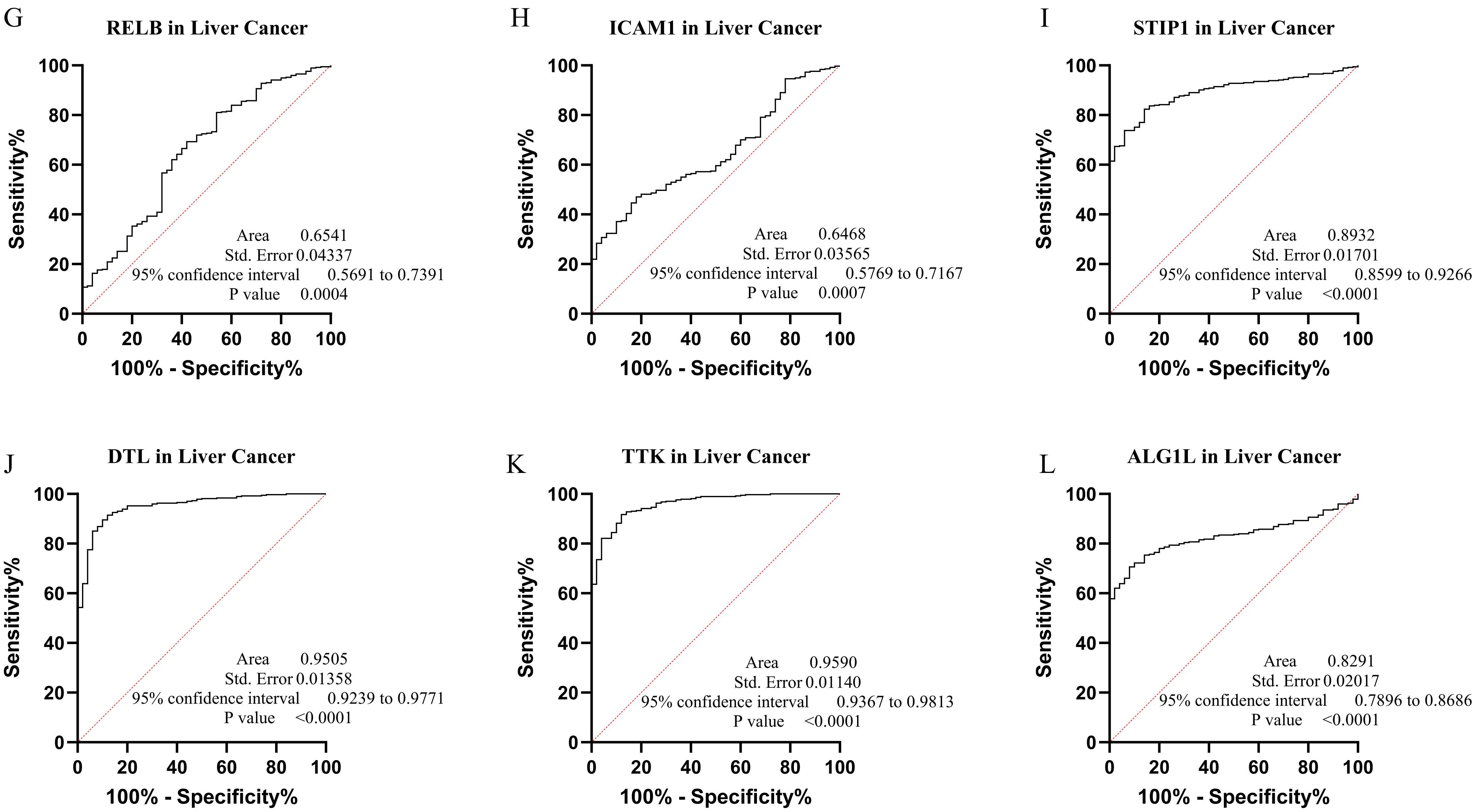

Suplementary Figure S11.

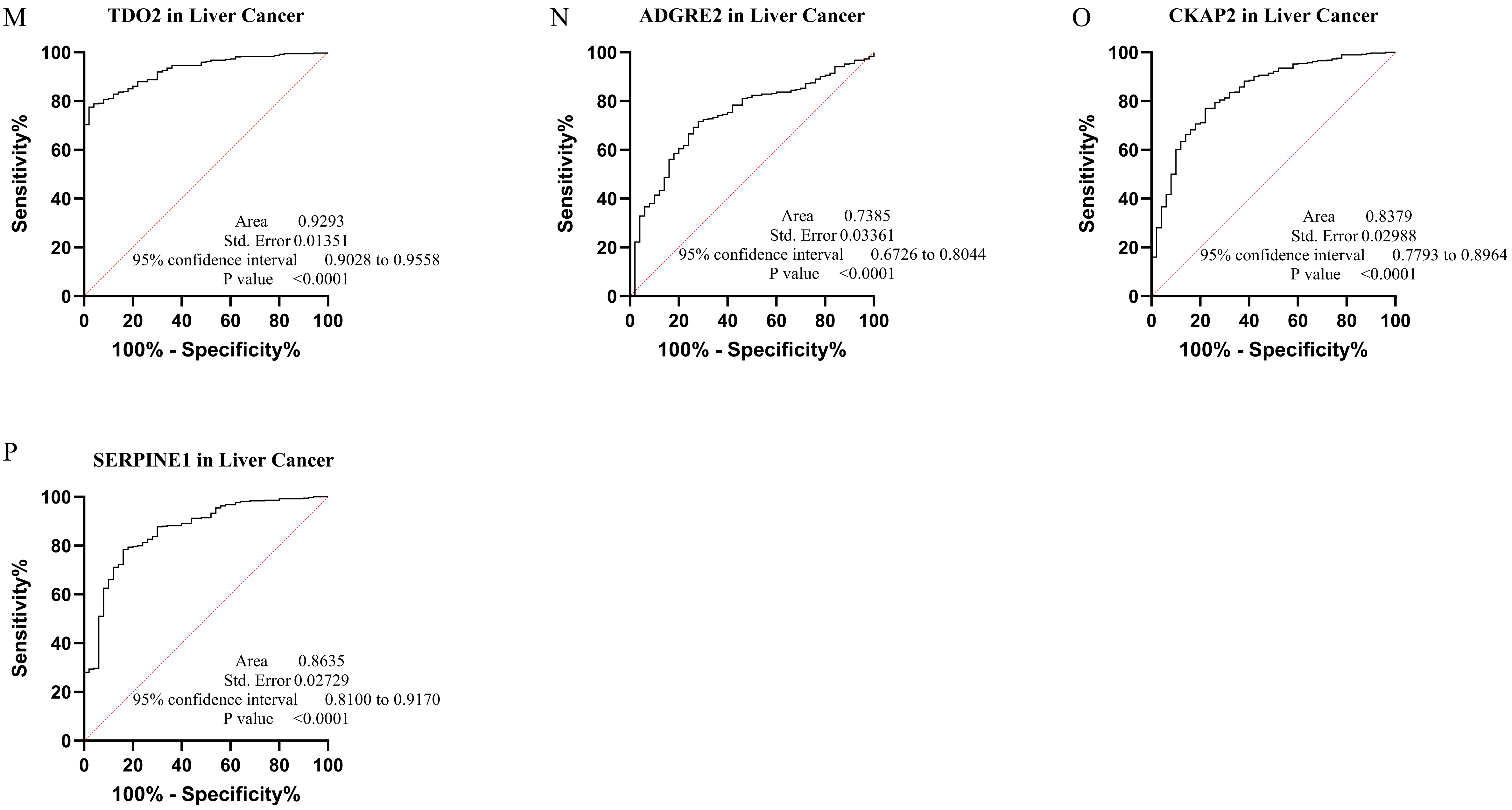

Suplementary Figure S12.

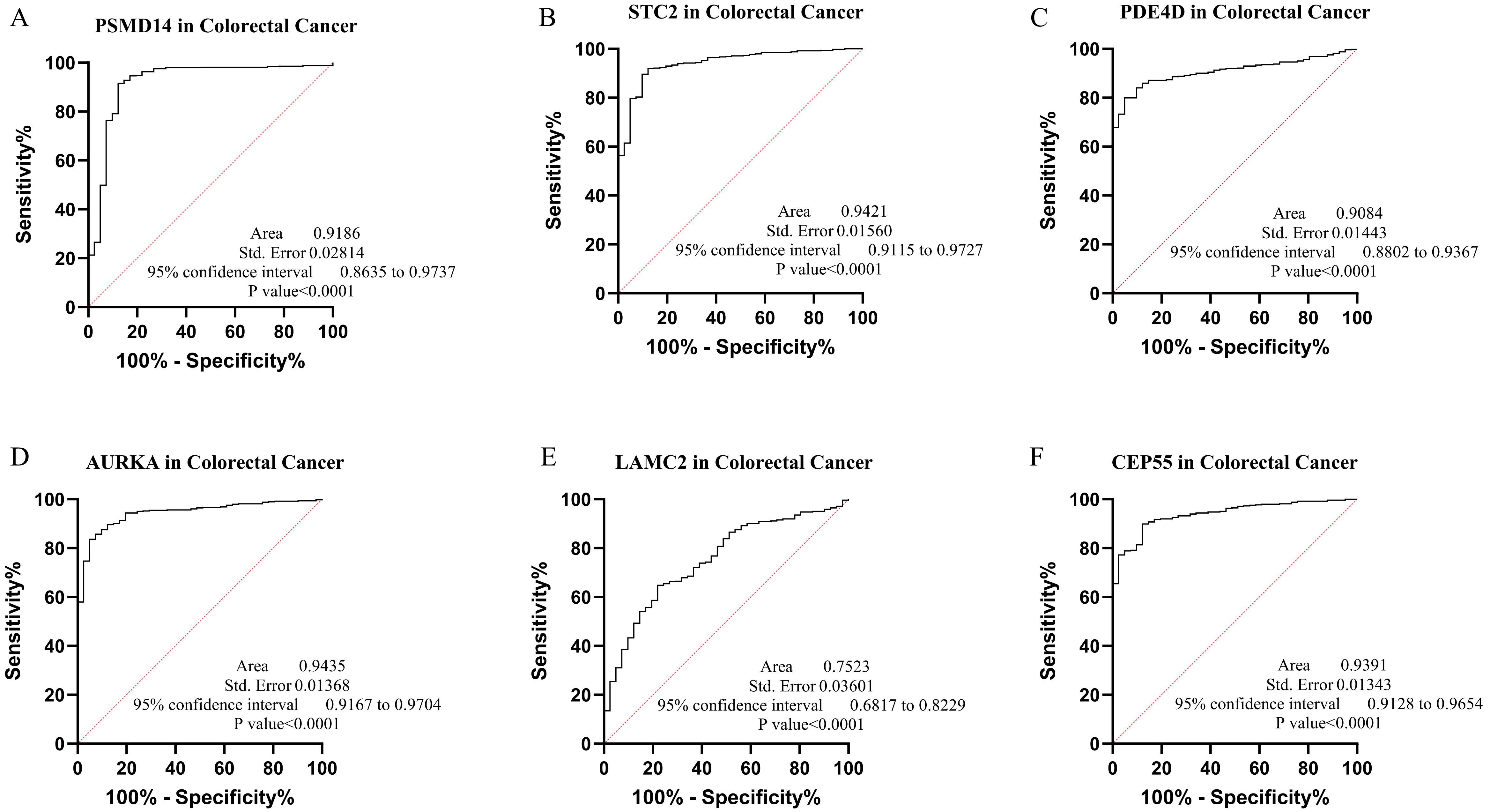

Suplementary Figure S12.

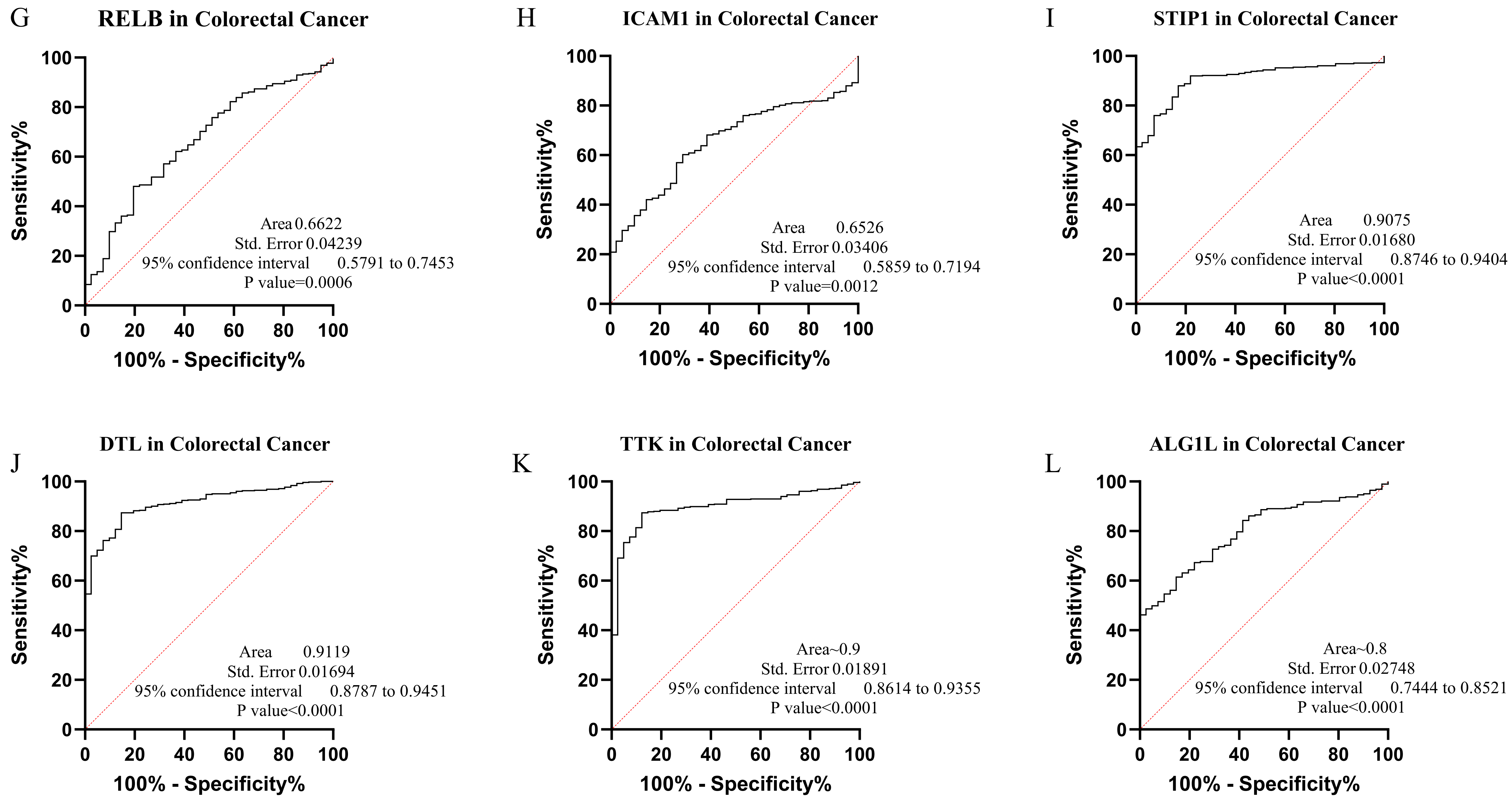

Suplementary Figure S12.

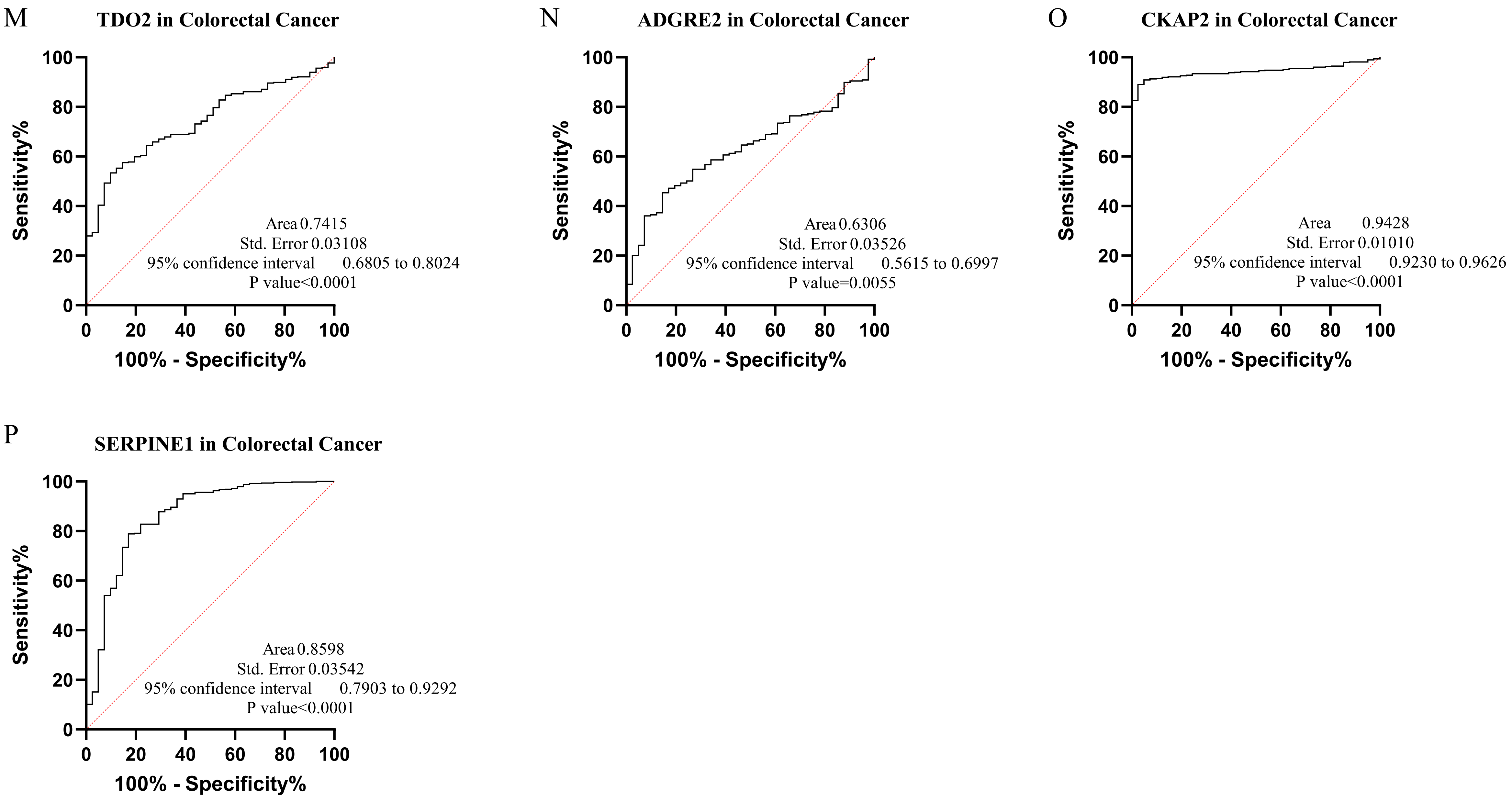

Supplement: Supplementary file 1 [file biology-14-00803-s001.zip › biology-3620101-supplementary.pdf]
